# Inhibiting multiple forms of cell death optimizes ganglion cells survival after retinal ischemia reperfusion injury

**DOI:** 10.1038/s41419-022-04911-9

**Published:** 2022-05-30

**Authors:** Qiyu Qin, Naiji Yu, Yuxiang Gu, Weishaer Ke, Qi Zhang, Xin Liu, Kaijun Wang, Min Chen

**Affiliations:** 1 Eye Center, the Second Affiliated Hospital, Medical College of Zhejiang University, Hangzhou, Zhejiang Province China; 2 Zhejiang Provincial Key Lab of Ophthalmology, Hangzhou, Zhejiang Province China

**Keywords:** Cell death, Neurodegeneration, Cell death in the nervous system

## Abstract

Progressive retinal ganglion cells (RGCs) death that triggered by retinal ischemia reperfusion (IR), leads to irreversible visual impairment and blindness, but our knowledge of post-IR neuronal death and related mechanisms is limited. In this study, we first demonstrated that apart from necroptosis, which occurs before apoptosis, ferroptosis, which is characterized by iron deposition and lipid peroxidation, is involved in the whole course of retinal IR in mice. Correspondingly, all three types of RGCs death were found in retina samples from human glaucoma donors. Further, inhibitors of apoptosis, necroptosis, and ferroptosis (z-VAD-FMK, Necrostatin-1, and Ferrostatin-1, respectively) all exhibited marked RGC protection against IR both in mice and primary cultured RGCs, with Ferrostatin-1 conferring the best therapeutic effect, suggesting ferroptosis plays a more prominent role in the process of RGC death. We also found that activated microglia, Müller cells, immune responses, and intracellular reactive oxygen species accumulation following IR were significantly mitigated after each inhibitor treatment, albeit to varying degrees. Moreover, Ferrostatin-1 in combination with z-VAD-FMK and Necrostatin-1 prevented IR-induced RGC death better than any inhibitor alone. These findings stand to advance our knowledge of the post-IR RGC death cascade and guide future therapy for RGC protection.

## Graphical abstract

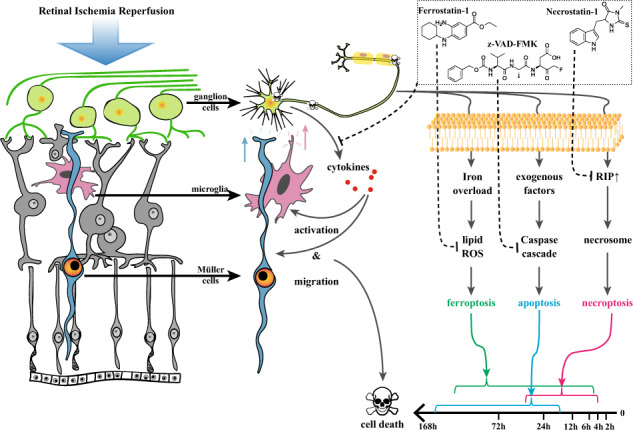

## Introduction

Characterized by the progressive degeneration of retinal ganglion cells (RGCs), retinal ischemia reperfusion (IR) represents a common denominator in various vision-threatening diseases, such as retinal vein occlusion and especially glaucoma [1]. In glaucoma, the rapid occlusion of retinal blood flow caused by transient intraocular hypertension triggers a self-reinforcing destructive cascade involving neuronal depolarization, calcium influx, and blood-retina barrier breach. Upon cessation of the ischemia, blood reperfusion brings oxygen and glucose, but also leads to severe free radical burst and excessive activated inflammatory responses. This then overwhelms normal cellular antioxidant defense mechanisms, ultimately ending with cell death [2].

Historically, the predominant mechanism of neuronal death was thought to be apoptosis. However, focusing on apoptosis alone has so far proved disappointing, with research indicating that RGCs saved by Caspase inhibition may not be able to recover, but eventually go on to die due to mitochondrial dysfunction [3]. After that, surge of interest has shifted to an inflammatory form of programmed cell death, necroptosis [4]. Early events in necroptosis include the activation of RIP1, which then binds to RIP3. Phosphorylated RIP3 recruits and then phosphorylates MLKL, forming a complex termed necrosome and resulting in irreversible cell death [5]. Direct evidence for necroptosis in regulating the retinal IR machinery was proposed based on reports that Necrostatin-1 (Nec-1), a special RIP1 inhibitor, could delay disease progression in a mouse retinal IR model [6].

Ferroptosis is a novel type of cell death that was first reported in 2012 [7]. Mechanistically, lipid peroxidation, GSH depletion, and ferrous iron accumulation are major hallmarks of ferroptotic cell death progression [8]. Currently, ferroptosis has been demonstrated to be inextricably associated with multiple types of neurological disorders, such as Parkinson’s and Alzheimer’s diseases [9]. In contrast, evidence of the role of ferroptosis in the pathogenesis of RGC death is limited. Only one study has reported that deferoxamine, an iron chelator, is protective against RGC and optic nerve fiber loss in cases of chronic ocular hypertension [10]. However, direct evidence of ferroptotic RGC and related iron-dependent mechanisms in acute retinal IR remain to be explored.

Given that the aetiology of neurological diseases is complex, where one cell death mechanism often in conjunction with the other drive pathology, in this study, we not only elucidated the role of ferroptosis in RGC loss and highlighted different importance of different cell death forms during IR, but also delineated the interacting molecular aspects between apoptosis, necroptosis, and ferroptosis and evaluated the effects of combined cell death inhibitors on neuronal rescue after retinal IR. The insights gained from this study will advance our knowledge of RGC death signaling and will be essential for planning future neuroprotective methods.

## Results

### Neuronal death after retinal IR shares features of apoptosis, necroptosis, and ferroptosis

**Fig. 1:**
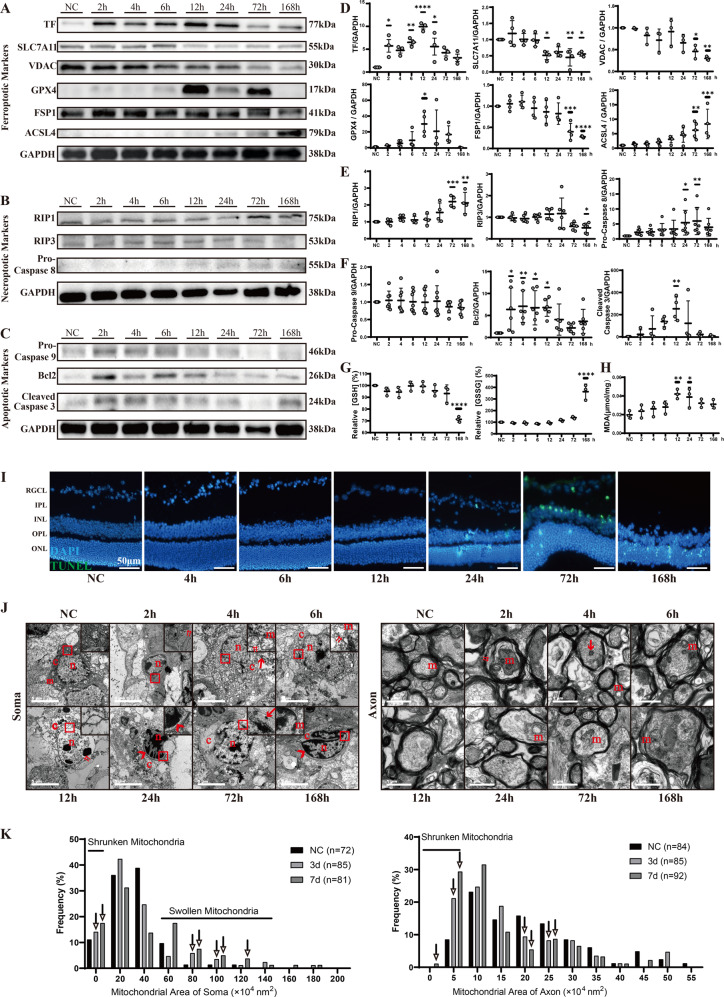
Ferroptosis, necroptosis and apoptosis are all involved in retinal IR-caused RGCs loss. **A**, **B**, **C** Western blot bands of the indicated proteins (**A** ferroptosis, **B** necroptosis, and **C** apoptosis) in total retina at different times (0–168 h) after IR injury. **D**, **E**, **F** Quantitative analysis of the protein expressions of ferroptotic markers (**D**: TF, SLC7A11, VDAC, GPX4, FSP1, ACSL4), necroptotic markers (**E**: RIP1, RIP3, pro-Caspase 8), apoptotic markers (**F**: pro-Caspase 9, Bcl2, cleaved Caspase 3), and GAPDH (*n* = 3–7). **G** Relative retina GSH and GSSG contents after IR injury were determined by relative assay kits (*n* = 3). **H** Malondialdehyde (MDA) concentration in the retina at different times after IR injury (*n* = 3). Data in (**D**–**H**) are represented as mean ± SD; *p < 0.05, **p < 0.01, ***p < 0.001, ****p < 0.0001 versus NC; one-way ANOVA with Bonferroni post hoc analysis. **I** Representative images of TUNEL + cells (green) in the retina at different times after IR injury. Nucleus was marked with DAPI (blue). Scale bar = 50 μm. **J** Ultrastructural change of RGC somas (left) and axons (right) following IR (*n* = 3). n nuclei, c cytoplasm, m mitochondria, red arrows, shrunken mitochondria; red double arrows, organelle incompleteness or nuclear membrane rupture; red arrowhead, chromatin condensation; red square, the area of each inset. Scale bar = 5 μm (left) or 1 μm (right). **K** Mitochondrial area frequency in somas (left) and axons (right). Arrows indicate different frequencies of shrunken or swollen mitochondria in IR groups (number of soma mitochondria, NC: *n* = 72; IR 3 d: *n* = 85; IR 7 d: *n* = 81; number of axon mitochondria, NC: *n* = 84; IR 3 d: *n* = 85; IR 7 d: *n* = 92). NC normal control, IR ischemia reperfusion, RGCL retinal ganglion cell layer, IPL inner plexiform layer, INL inner nuclear layer, OPL outer plexiform layer, ONL outer nuclear layer.

The expression of key proteins relating to apoptosis, ferroptosis and necroptosis was examined to investigate the regulated cell death scenarios in retinal IR (Table 1). Notably, only 2 h after IR, TF showed remarkable elevation and peaked at 12 h, then gradually decreased. In addition, membrane protein SLC7A11 was highly retained in the early stage and then exhibited a half descent (Fig. 1A, D). Meanwhile, parallel with the change in GSH level (Fig. 1G), VDAC and enzymes that protect lipids from peroxidation, such as GPX4 and FSP1, were finally downregulated (Fig. 1A, D). Further, ACSL4 enhanced progressively over time (Fig. 1A, D). The production of lipid peroxidation, Malondialdehyde (MDA), also showed a gradual increase after IR injury (Fig. 1H). RIP1 expression elevated at late stage (72 and 168 h post-IR), while RIP3 expression simultaneously decreased (Fig. 1B, E). The co-staining of GPX4 or RIP1 with RGCs showed a similar trend with that of Western blot (Fig. S1). Along with RIP1, the level of pro-Caspase 8 also significantly elevated at a late stage (72 h; Fig. 1B, E). However, pro-Caspase 9 remained continually steady, and Bcl2 only elevated in the initial 12 h (Fig. 1C, F). This evidence indicates that necroptosis may occur at an early stage but not intensely, as RIP3 still remained before a sudden drop at 72 h. Apoptosis may happen rigorously a little later, resulting in an increased level of cleaved Caspase 3 peaking at 12 h and TUNEL-positive RGCs starting at 24 h (Fig. 1C, F, I). In turn, ferroptosis may cover the whole period, which was early potentiated by abnormal iron-metabolic mechanisms and later prevailed by obvious lipid metabolic disorder.

**Table 1:** Targets and markers of inhibitors and their different mechanisms involved in cell death pathways.

Inhibitors	Targets	Pathway	Markers	Associated death mechanisms
Fer-1	Lipid peroxidation	Ferroptosis	TF	Transports iron to sites of storage and utilization [48]
			SLC7A11	Functions to import cystine for glutathione biosynthesis and antioxidant defense [49]
			VDAC	Channels through the mitochondrial outer membrane that governs global mitochondrial metabolism [50]
			ACSL4	An important isozyme for polyunsaturated fatty acids metabolism that dictates ferroptosis sensitivity [33]
			GPX4^1^	Converts lipid hydroperoxides to lipid alcohols and prevents the iron-dependent formation of toxic lipid ROS [51]
			FSP1^1^	A CoQ oxidoreductase that traps lipid peroxides and confers protection against ferroptosis elicited by GPX4 deletion [19]
zVAD	Pan Caspase	Intrinsic (endogenous) apoptosis	Pro-Caspase 9	Forms a complex with cytochrome c and Apaf-1, leading to the activation of the protease, which cleaves and activates Caspase 3 [52]
			Cleaved Caspase 3	Activates cascade of Caspases to execute apoptosis
			Bcl2^1^	Inhibits Caspase activity
		Extrinsic (exogenous) apoptosis	Pro-Caspase 8	Interacts with FADD to control TNF-mediated apoptosis, necroptosis, and inflammatory pathways [53]
			Cleaved Caspase 3	Ditto
			Bcl2^1^	Ditto
Nec-1	RIP1	Necroptosis	Pro-Caspase 8	Ditto
			RIP1	Triggered by death receptors to form a RIP1-RIP3-MLKL complex
			RIP3	Phosphorylates MLKL that leads to necroptosis via disruption of plasma membrane and cell lysis [54]

^1^ Indicates protective genes in the pathways.

*Fer-1* Ferrostatin-1, *zVAD* z-VAD-FMK, *Nec-1* Necrostatin-1, *TF* transferrin, *SLC7A11* solute carrier family 7 member 11, *VDAC* voltage-dependent anion channel, *ACSL4* acyl-CoA synthetase long chain family member 4, *GPX4* glutathione peroxidase 4, *FSP1* ferroptosis suppressor protein 1, *Bcl2* B-cell leukemia 2, *ROS* reactive oxygen species.

To further elucidate the chronological order of apoptosis, necroptosis, and ferroptosis following IR, we captured the ultrastructural images of RGCs via transmission electron microscopy (TEM). There was general swelling since 4 h after IR, and irreversible membrane rupture was captured since 6 h after IR (Fig. 1J, left). At 12 h, chromatin condensed into small irregular pieces, which was supposed to represent necroptosis until 24 h after IR. Apoptosis was detected from 24 h to 7 d as classic dense masses of chromatin distributed against the nuclear envelope. During the whole stage, general areas of plasma underwent a density decrease. Additionally, shrunken mitochondria with dense membrane and breaking crista were widely detected after 4 h (Fig. 1J, left). These results signaled that ferroptosis arose from 4 h to very late stage after IR. In addition to somas, we examined the axons of RGCs, in which destruction and demyelination were gradually exacerbated (Fig. 1J, right). The mitochondria in the axons showed similar lesions to that in the somas and fusion of mitochondria was discovered (Fig. 1J, right). The quantitative data showed that the frequency of smaller mitochondrial areas increased in both the cytoplasm and axons, while swollen mitochondrial areas mainly increased in the cytoplasm (Fig. 1K, left and right).

### Inhibition of ferroptosis, apoptosis and necroptosis could improve RGCs survival respectively and collectively both in vitro and in vivo

**Fig. 2:**
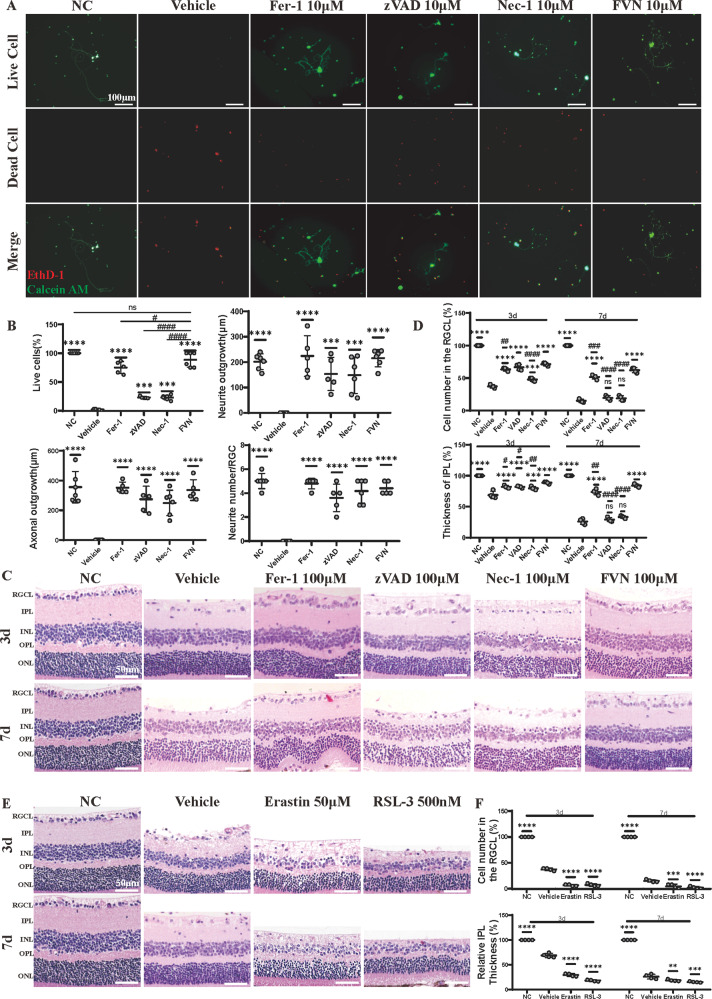
Fer-1, zVAD and Nec-1 improve RGC survival respectively and collectively both in vitro and in vivo. **A** Live/Dead assay staining for mouse primary RGCs that underwent OGD/R with vehicle treatment (DMSO) or 10 μM Fer-1, zVAD, Nec-1, or FVN (green for live cells, red for dead cells) and (**B**) statistical analysis of percentage of live cells, neurite outgrowth, axonal outgrowth, and average neurite number (*n* = 5). Scale bar = 100 μm. **C** HE staining of retinal tissue in mice that underwent sham or IR injury at 3 and 7 d after intravitreal injection of vehicle (DMSO) or 100 μM Fer-1, zVAD, Nec-1, or FVN and (**D**) analysis of RGC number and IPL thickness (*n* = 4). Scale bar = 50. **E** HE staining of retinal tissue in mice that underwent sham or IR injury at 3 and 7 d after intravitreal injection of vehicle (DMSO) or 50 μM Erastin and 500 nM RSL-3 and (**F**) analysis of RGC numbers and IPL thickness (*n* = 4). Scale bar = 50 μm. Data in (**B**, **D**, **F**) are represented as mean ± SD; *p < 0.05, **p < 0.01, ***p < 0.001, ****p < 0.0001 versus vehicle; ^#^*p* < 0.05, ^##^*p* < 0.01, ^###^*p* < 0.001, ^####^*p* < 0.0001 versus FVN; one-way ANOVA with Bonferroni post hoc analysis. NC normal control, OGD/R oxygen glucose deprivation/reoxygenation, IR ischemia reperfusion, RGCL retinal ganglion cell layer, IPL inner plexiform layer, INL inner nuclear layer, OPL outer plexiform layer, ONL outer nuclear layer, FVN combination of Fer-1, zVAD, and Nec-1.

Primary RGC death after oxygen glucose deprivation/reoxygenation (OGD/R) damage was remarkably alleviated after treatment with different dosages of Fer-1 (Ferrostatin-1), zVAD (z-VAD-FMK), and Nec-1, with the most effective dose to be 10 μM in all groups (Figs. 2A, S2 and S3). Except for survival rate, morphological changes of RGCs including average length of neurite outgrowth, axonal outgrowth, and average neurite number were also analyzed and both showed consistent good effects as living RGCs percent in each treatment group (Fig. 2B). Continuing in vivo observations also supported the protective role of Fer-1, zVAD, and Nec-1 in rescuing RGCs loss 3 d after IR, as measured by inner plexiform layer (IPL) thickness and cell number of RGC layer (RGCL) after hematoxylin-eosin (HE) staining (Fig. 2C upper row, 2D and S4). However, unlike Fer-1, which could still protect about 50% of RGCs from death 7 d after IR, no significant improvement in RGC survival was observed in the zVAD and Nec-1 groups by the end of the observation period (Fig. 2C, lower row and 2D). Furthermore, the combination treatment of cell death inhibitors (FVN: the combination of Fer-1, zVAD, and Nec-1) showed better RGC protective effects than either single inhibitor alone both in vitro and in vivo (Fig. 2A–D). Generally, Nec-1, zVAD, Fer-1, and their combination saved RGCs to a moderate to strong extent in turn. The retinal immunofluorescent staining and the protein expression level of NeuN also paralleled the findings of the histological examination (Fig. S5). In particular, we verified that RGC injury after IR could be aggravated in vivo by Erastin, an activator of ferroptosis, and RSL-3, an inhibitor of GPX4, which establishes a link between retinal IR and ferroptosis more sufficiently (Fig. 2E, F).

### Fer-1, zVAD and Nec-1 work through their targets to reduce OGD/R-induced primary RGCs damage

**Fig. 3:**
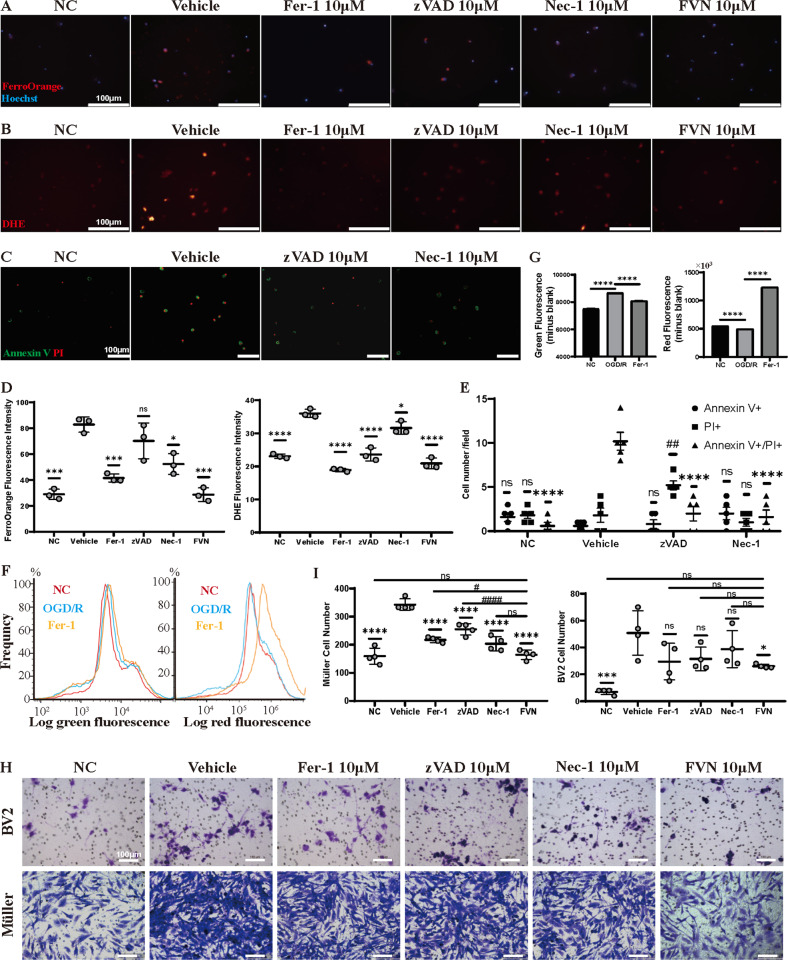
Fer-1, zVAD, and Nec-1 work through their targets to reduce OGD/R-induced primary RGC damage. **A** FerroOrange probe (red) staining for intracellular Fe^2+^ in mouse primary RGCs that underwent OGD/R with vehicle treatment or 10 μM Fer-1, zVAD, Nec-1, or FVN. Scale bar = 100 μm. **B** Dihydroethidium (DHE) staining for ROS (red) in mouse primary RGCs that underwent OGD/R with the same FerroOrange treatment. Scale bar = 100 μm. **C** Annexin V and propidium iodide (PI) staining for apoptosis and necrosis in mouse primary RGCs that underwent OGD/R with vehicle treatment or 10 μM zVAD or Nec-1 (green for Annexin V+, red for PI+). Scale bar = 100 μm. **D** Statistical analysis of fluorescence intensity for FerroOrange and DHE (*n* = 3). **E** Statistical analysis of the number of RGCs with Annexin V+, PI+, or Annexin V+/PI+ (*n* = 5). Data in (**E**) are represented as mean ± SD; ****p < 0.0001 versus vehicle in Annexin V+/PI+ cells; ^##^*p* < 0.01 versus vehicle in PI+ cells; one-way ANOVA with Bonferroni post hoc analysis. **F** Histogram showing green and red fluorescence changes after staining with C11 BODIPY in mouse RGCs that underwent OGD/R with vehicle treatment or 10 μM Fer-1 and (**G**) statistical analysis of fluorescence intensity. **H** Chemotaxis of the supernatants from cultured RGCs in each group toward Müller or BV2 microglial cell lines was tested by 24 h Transwell assay and following crystal violet staining and (**I**) statistical analysis of cell number (*n* = 4). Scale bar = 100 μm. Data in (**D**, **G**, **I**) are represented as mean ± SD; *p < 0.05, **p < 0.01, ***p < 0.001, ****p < 0.0001 versus vehicle or OGD/R; ^#^*p* < 0.05, ^##^*p* < 0.01, ^###^*p* < 0.001, ^####^*p* < 0.0001 versus FVN; one^-^way ANOVA with Bonferroni post hoc analysis. NC normal control, OGD/R oxygen glucose deprivation/reoxygenation, FVN combination of Fer-1, zVAD, and Nec-1.

It is widely accepted that the excessive accumulation of lipid peroxides, which are contingent upon the availability of iron and reactive oxygen species (ROS), contributes to final cell ferroptosis [11]. The results of Fe^2+^ probing showed that primary RGCs exposed to OGD/R exhibited considerably increased intracellular Fe^2+^ compared with the control group. However, Fer-1, Nec-1, or FVN treatment significantly blocked excessive iron generation (Fig. 3A, D). ROS detection showed that increased ROS levels were mostly weakened by Fer-1 and partly by zVAD and Nec-1 (Fig. 3B, D). The results of the C11 BODIPY probe showed that cultured RGCs treated with Fer-1 exhibited alleviation of the increased lipid ROS after OGD/R injury to some extent (Fig. 3F, G). zVAD exhibited a great effect in protecting RGCs from OGD/R-induced apoptosis, but promoted the occurrence of cell necroptosis, as shown by decreased Annexin V positive cells and increased single Propidium iodide (PI) positive cells. After Nec-1 treatment, however, both Annexin V + /PI + and PI + RGCs were significantly reduced (Fig. 3C, E).

RGCs undergoing demise can be engulfed by neighboring phagocytes, such as Müller glia and microglial cells [12, 13]. Here, we observed that supernatants from RGCs with OGD/R damage recruited more Müller and microglial cells. However, the cells’ migration behavior was significantly inhibited when the supernatants were isolated from RGCs treated with either Fer-1, zVAD, Nec-1, or FVN (Fig. 3H, I). Further, since Müller cells migration varied significantly in different groups, to get rid of the direct effect of the inhibitors on cell migration, we applied each single inhibitor (10 μM) to Müller cells after OGD/R and found inhibitors at this concentration had no impact on cell survival and chemotaxis (Fig. S6).

### In situ administration of Fer-1, zVAD, Nec-1 and their combination alters the expression of death pathway- and neurogenesis-related proteins

**Fig. 4:**
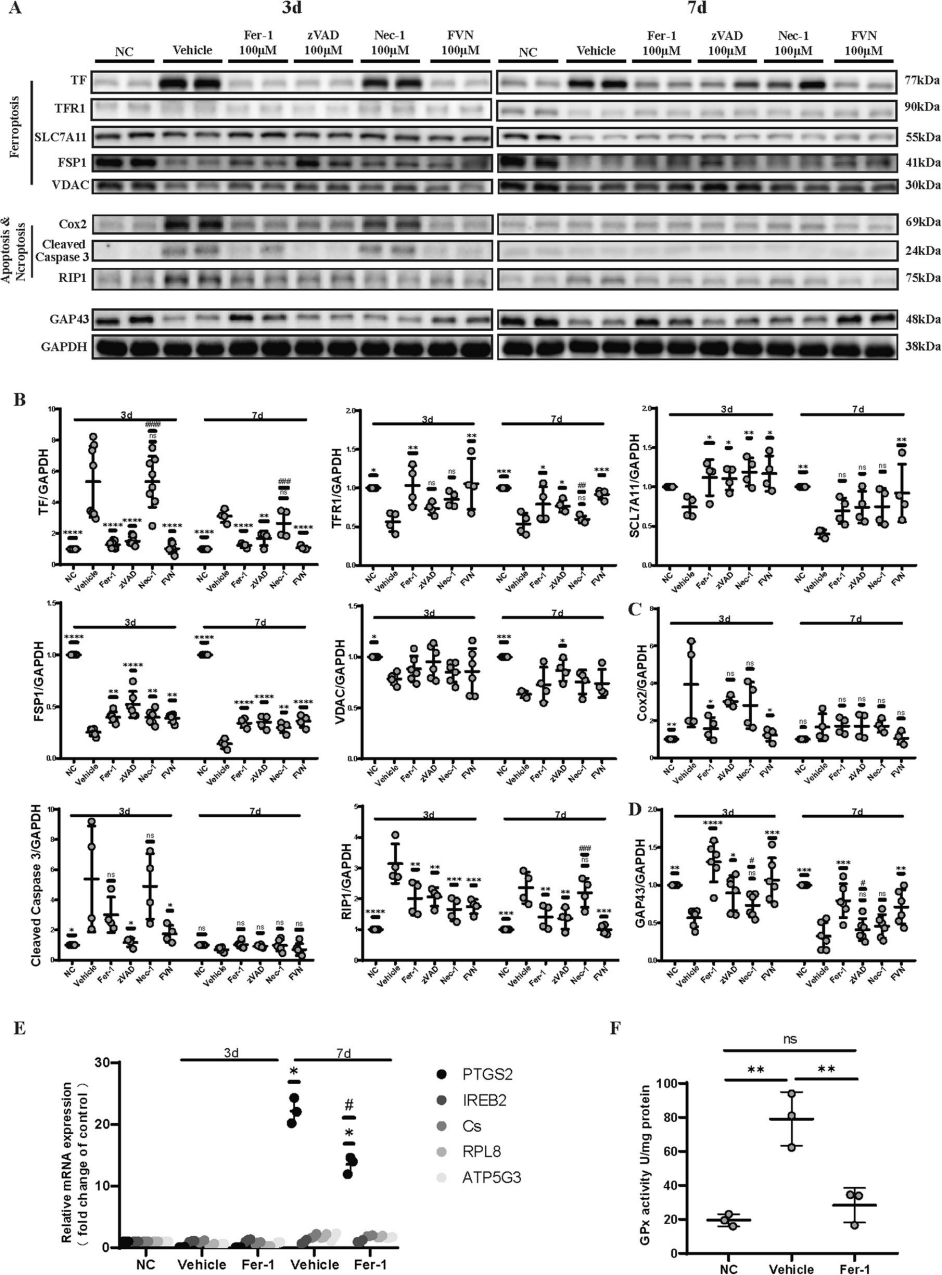
In situ administration of Fer-1, zVAD, Nec-1, and their combination alters the expression of death pathway- and neurogenesis-related proteins. **A** Western blot bands of the indicated proteins in sham or IR-injured retina 3 and 7 d after each treatment and (**B**, **C**, **D**) quantitative analysis of the protein expression levels of ferroptotic markers (**B**: TF, TFR1, SLC7A11, FSP1, VDAC), apoptotic and necroptotic markers (**C**: Cox2, cleaved Caspase 3, RIP1), neurogenesis-related marker (**D**: GAP43), and GAPDH (*n* = 4–6). **E** Expression of the genes involved in ferroptosis was examined in sham or IR-injured retina by qRT-PCR at 3 and 7 d post-vehicle (DMSO) or post-Fer-1 treatment (*n* = 3). Data in E are represented as mean ± SD; *p < 0.05 versus NC; ^#^*p* < 0.05 versus vehicle; one-way ANOVA with Bonferroni post hoc analysis. **F** GPx activity of sham or IR-injured retina with vehicle (DMSO) or Fer-1 treatment was measured with a GPx assay kit at 3 d post-IR (*n* = 3). Data in (**B**–**D**, **F**) are represented as mean ± SD; *p < 0.05, **p < 0.01, ***p < 0.001, ****p < 0.0001 versus vehicle; ^#^*p* < 0.05, ^##^*p* < 0.01, ^###^*p* < 0.001, ^####^*p* < 0.0001 versus FVN; one-way ANOVA with Bonferroni post hoc analysis. NC normal control, IR ischemia reperfusion, FVN combination of Fer-1, zVAD, and Nec-1.

TF levels, which elevated most significantly in the retina samples, showed remarkable reduction after Fer-1, zVAD, and FVN treatment at both 3 and 7 d post-IR (Fig. 4A, B). Correspondingly, TFR1 levels, which almost halved after IR damage, rebounded significantly after treatment with Fer-1, zVAD, and especially FVN. Additionally, SLC7A11 levels were suppressed after IR injury and nearly came back to normal in all groups, especially at 3 d after treatment. Some other proteins in ferroptosis, such as VDAC and especially FSP1, underwent similar expression changes. Furthermore, Cox2, which is usually regarded as an anti-apoptotic guard [14] and upregulated during ferroptosis in a recent study [15], was inhibited by Fer-1 (Fig. 4A, C). The elevation in cleaved Caspase 3 levels was nearly completely inhibited by zVAD but not influenced by Nec-1, even if the target of Nec-1, RIP1, can potentially induce apoptosis [16]. Certainly, RIP1 was inhibited in the zVAD group since it usually works when combined with pro-Caspase 8 and involved in apoptosis (Fig. 4A, C). Additionally, GAP43 and p-S6, which mark protein synthesis and neurogenesis and express at high level only when neurons survive with good viability and energy supply, although dampened following retinal IR, were significantly improved after each treatment (Figs. 4A, D and S7).

Finally, we assessed ferroptosis-related genes, including ATP5G3, RPL8, CS, and IREB2, as well as PTGS2 by qRT-PCR and found no changes in the expression of ATP5G3, RPL8, CS and IREB2 in retinas after IR injury, indicating that blood elements or longtime ischemia may be responsible for the upregulation of these genes in vivo [17]. However, PTGS2 mRNA levels markedly increased and Fer-1 alleviated this effect at 7 d post-IR (Fig. 4E). We also assessed GPx activity which elevated significantly at 3 d post-IR, while Fer-1 led them to near normal levels (Fig. 4F).

### In situ administration of Fer-1, zVAD, Nec-1 and their combination mitigates activated microglial, Müller cells and inflammatory responses, but not neovascularization

**Fig. 5:**
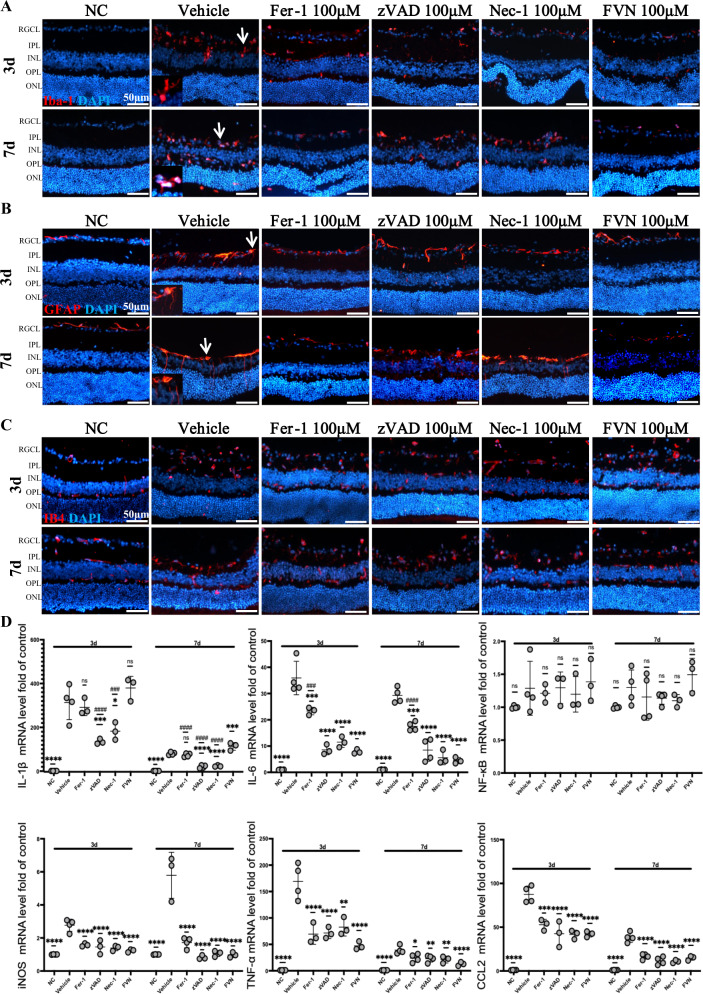
In situ administration of Fer-1, zVAD, Nec-1, and their combination mitigates activated microglia, Müller cells, and inflammatory responses, but not neovascularization. **A**, **B**, **C** Representative images of microglia, Müller cells, and neovascularization in sham or IR-injured retina 3 and 7 d after each treatment. Microglia and Müller cells were marked with Iba-1 and GFAP, respectively (red). An inset with an enlarged magnification of the area pointed out with a white arrow was added to the original picture in the bottom-left to exhibit the typical morphological character of activated microglia and Müller cells in the IR-vehicle group. Neovascularization was marked with IB4 (red). Nucleus was marked with DAPI (blue). Scale bar = 50 μm. **D** Expression of genes coding for inflammation markers was measured in sham and injured retina by qRT-PCR at 3 and 7 d after each treatment (*n* = 4). Data in (**D**) are represented as mean ± SD; *p < 0.05, **p < 0.01, ***p < 0.001, ****p < 0.0001 versus vehicle; ^#^*p* < 0.05, ^##^*p* < 0.01, ^###^*p* < 0.001, ^####^*p* < 0.0001 versus FVN; one-way ANOVA with Bonferroni post hoc analysis. NC normal control, IR ischemia reperfusion, RGCL retinal ganglion cell layer, IPL inner plexiform layer, INL inner nuclear layer, OPL outer plexiform layer, ONL outer nuclear layer, FVN combination of Fer-1, zVAD, and Nec-1.

Both 3 and 7 d after initial ischemia in mice, we observed obvious activation of microglial and Müller cells, as reflected in typical morphologic alterations and the promoted expression of Iba-1 and GFAP (Figs. 5A, B and S8). Meanwhile, inflammatory factors, such as IL-1β, IL-6, iNOS, TNF-α, and CCL2, which could contribute to further injury to neurons and tissues, were also released to defend against external invasions, as assessed by qRT-PCR, but the gene expression of NF-κB did not change. By treatment with either zVAD, Nec-1, Fer-1, or FVN, Iba-1 and GFAP were greatly downregulated both in shape and quantity (Figs. 5A, B and S8), especially at 3 d post-IR. Inflammatory cytokines also decreased, with zVAD and Nec-1 showing a more robust inhibition of IL-1β and IL-6 than Fer-1 (Fig. 5D). However, the effect of zVAD, Nec-1, Fer-1, and FVN on retina neovascularization after IR was imperceptible, as measured by both fluorescent staining and Western blots (Figs. 5C and S8).

### RGCs in retina samples from human end-stage glaucoma donors undergo apoptosis, necroptosis and ferroptosis

**Fig. 6:**
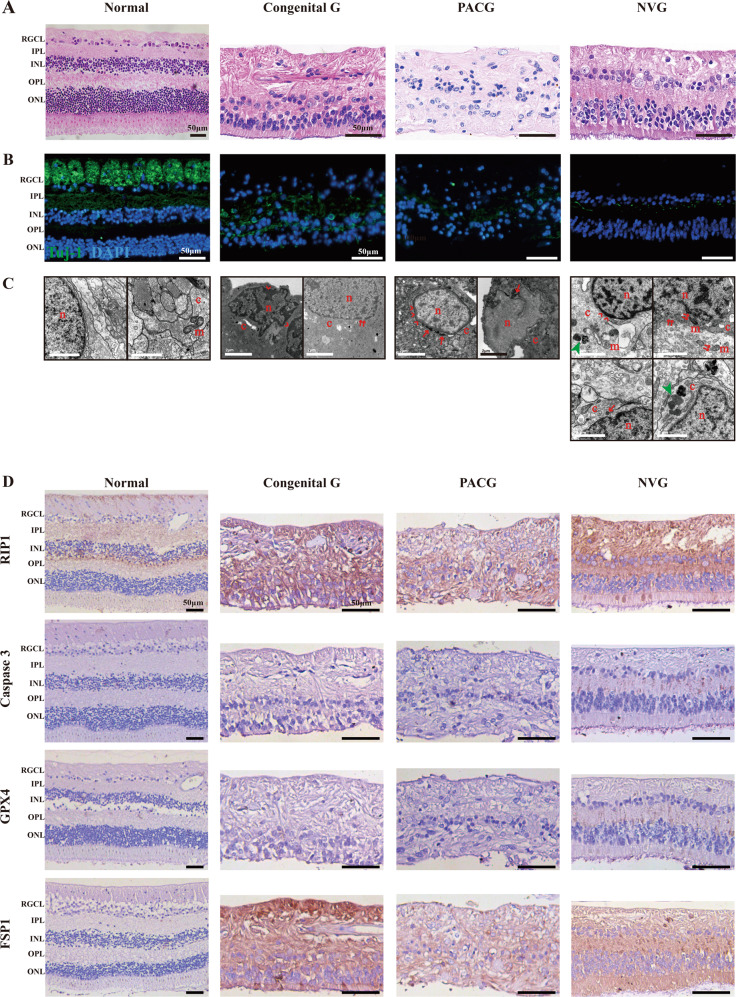
RGCs in retina samples from end-stage glaucoma patients undergo apoptosis, necroptosis and ferroptosis. **A** HE staining from a normal human donor and patients with congenital glaucoma (Congenital G), primary angle-closure glaucoma (PACG) and neovascular glaucoma (NVG). Scale bar = 50 μm. **B** Representative images of neuronal marker in retinal tissue from human eyes corresponding to HE staining. RGCs were marked with Tuj-1 (green) and nucleus with DAPI (blue). Scale bar = 50 μm. **C** Ultrastructural change of RGC somas from human eyes corresponding to HE staining. n nuclei, c cytoplasm, m mitochondria, red arrows, shrunken mitochondria; red double arrows, organelle incompleteness or nuclear membrane rupture; red arrowhead, chromatin condensation; green arrow head, autophagolysosome. Scale bar = 2 μm. **D** Immunohistochemistry of RIP1, Caspase 3, GPX4, and FSP1 expression in retinal tissue from human eyes corresponding to HE staining. Scale bar = 50 μm. RGCL retinal ganglion cell layer, IPL inner plexiform layer, INL inner nuclear layer, OPL outer plexiform layer, ONL outer nuclear layer.

To date, no reports have proved the existence of necroptosis and ferroptosis in retina samples from human glaucoma donors. In this study, HE and immunofluorescent staining demonstrated that the RGCs in end-stage glaucoma donors were almost completely lost and the retinal structure significantly scrambled (Fig. 6A, B). The residual RGCs in congenital, primary angle-closure and neovascular glaucoma patients showed morphological abnormalities indicative of apoptosis, necroptosis, ferroptosis, and autophagy (Fig. 6C).

The results of immunohistochemistry showed that RIP1 was expressed not only in RGCs but in the whole retina, even in the normal one, and was significantly upregulated in glaucoma patients (Fig. 6D). The Caspase 3-positive RGCs slightly appeared in the glaucoma retinas but were not very obvious. The phosphatidylserine exposed on the surface of apoptotic cells, which was regarded as an “eat me” signal, could make the apoptotic RGCs easier to be cleared by the glia via efferocytosis. Thus, less Caspase evidence in the final tissue sections is possible [18]. GPX4 was expressed in a low level in both normal and glaucoma retinas, but FSP1 was significantly elevated in the RGCL of human glaucoma retinas. Although the expression of FSP1 is usually parallel with GPX4, as we showed in the mouse model, FSP1 is independent of GPX4. FSP1 needs NAD(P)H to achieve its function while the respiratory chain works diversely under perfusion and non-perfusion situations [19]. Actually, unlike the acute IR mouse model in which the retina was finally perfused with oxygen and glucose, these end-stage glaucoma patients were more likely to suffer from chronic sustained intraocular hypertension injury, as the latest intraocular pressure before eye enucleation of these patients was still 2-3 folds higher than the upper limit of normal (Table S1). The chronic deficiency of the hydrogen donor NAD(P)H in these patients may account for the compensatory elevation of FSP1.

## Discussion

The newly proposed concept of PAN-optosis, which was first raised in 2019 [20], suggests the involvement of different yet highly interconnected cell death processes in a particular disease. Here, our work first found that during retinal IR injury, necroptosis in RGCs occurs first, followed by apoptosis after some delay, whereas ferroptosis is involved in the whole course.

It is widely accepted that necroptosis is a “fail-safe” mechanism to ensure that cell death can still occur when apoptosis stalls, as its onset is generally associated with the inhibition of Caspases, particularly Caspase 8. Actually, Caspase 8 favors apoptosis over necroptosis, so it prevents the formation of necrosomes to some extent [21]. As Caspase 8 activity is partially dependent on ATP level [22, 23], a shortage in ATP production in the acute stage of IR may explain why the increase of Caspase 8 and apoptosis did not appear immediately after the initial IR attack. Our results that applying single zVAD or Nec-1 had relatively poor benefits on RGC survival and the application of zVAD in primary RGCs underwent OGD/R could promote cell necroptosis also fit the mainstream view of a competitive relation between apoptosis and necroptosis [24, 25].

**Fig. 7:**
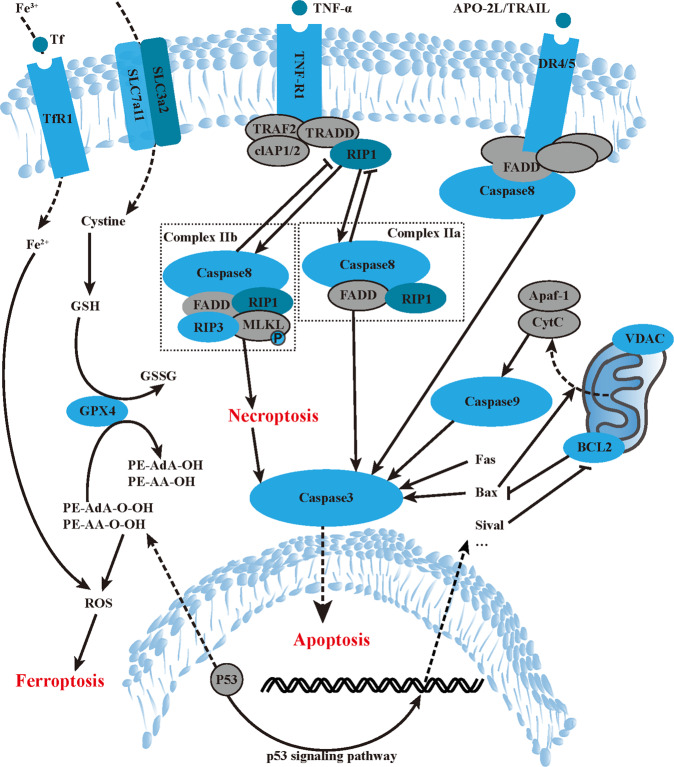
Diagram of apoptosis, necroptosis, and ferroptosis pathway. During ischemia reperfusion injury, apoptosis, necroptosis and ferroptosis pathway are activated. Transferrin upregulation brings intracellular iron accumulation while cystine-transportation and GPX4 dysfunction brings phospholipids peroxidation. Finally lipid ROS increases and activates ferroptosis. TNF-α activates its receptor, resulting in formation of Complex IIa or Complex IIb, and leads to necroptosis or apoptosis respectively. Apoptosis can also originate from some other extrinsic or intrinsic signals, such as Apo2 Ligand or cytochrome c. The crosstalks among these pathways are complicated and need study more.

Iron is an indispensable component for ferroptosis and plays the role of electron donor to oxygen for ROS formation. The TF/TFR1 is the main regulator of iron uptake in cells. During ferroptosis, the ferric iron Fe^3+^, which is carried by TF, binds to TFR1 and is internalized by cells [26]. After that, the STEAP3 enzyme reduces Fe^3+^ to ferrous iron Fe^2+^, and then the divalent metal transporter 1 releases it to the cytoplasm iron pool required for ferroptosis [27]. It was demonstrated that the acidic environment in brain tissue after cerebral ischemia can inhibit the pH-dependent affinity of TF for iron, resulting in final intracellular iron accumulation [28]. Similarly, we speculate that the sharply changed pH value, oxidation level and ion metabolism disturbance after IR may account for the early emergence of iron transport disruption and the occurrence of ferroptosis. As the overloaded Fe^2+^ facilitates the production of ROS via Fenton reaction and the generation of lipid hydroperoxides, which impair cellular structures such as membranes [29], it is conceivable that the immediate increase of iron metabolism after retinal IR is the initiation factor for ferroptosis, and the following lipid dysbolism secondary to iron accumulation may further enlarge the death area and prolong the death course (Fig. 7).

Crosstalks between ferroptosis and apoptosis/necroptosis are more elusive. Research has demonstrated that Fer-1 treatment can compensate for p53 dysfunction [30], and p53 is known for its apoptosis-inducing function. Thus, Fer-1 may enhance apoptosis. Meanwhile, GSH depletion results in ROS accumulation in ferroptosis. And the low GSH levels in the cytoplasm can cause low GSH uptake in the mitochondria, which can induce the downregulation of Bcl2 and the upregulation of Bax to initiate Caspase activation [31]. Notably, a recent study stated that dysfunction of the key ferroptosis-surveilling systems could hypersensitize mice to tubular necrosis during acute kidney injury, and the simultaneous inhibition of necroptosis and ferroptosis could significantly protect mice against bilateral renal IR injury [32]. The results of our study also revealed a complex interplay among these death forms. The obviously changed TF/TFR1 levels after IR injury could be rescued not only by the medication of Fer-1, but also the zVAD. And the significantly suppressed SLC7A11 levels after IR can nearly come back to normal in all groups, which suggests that apoptosis, necroptosis, and ferroptosis are all involved in dysregulated cysteine transportation and GSH synthesis during ischemic and hypoxic injury. Recently, it is coming into focus that ROS may act as a rheostat allowing different cell deaths to engage and crosstalk with each other [27]. Using primary RGCs, our study also showed that inhibitors of three different cell fates can both alleviate the increased intracellular ROS levels caused by OGD/R. However, the regulatory features of these crosstalks and how this ROS rheostat is controlled warrant further exploration. As these death forms seem alternative in that resistance to one pathway sensitizes cells to death via the other pathway, we believe that inhibiting multiple forms of cell death could optimize damaged cell rescue in a more comprehensive way.

Additionally, we explored how neurons, Müller cells, and microglia interacted and the mutual effects among them during IR. Injury like IR could trigger the activation of retinal microglia and Müller cells, resulting in the continuous secretion of inflammatory mediators, such as TNF-α and IL-1β, and aggravated excitatory neural toxicity [33–35]. Using a coculture system, Tezel and Wax showed that glia cells secreted TNF-α when exposed to simulated ischemia, which promoted the direct death of RGCs [36]. However, we are inclined to regard this process as bilateral, for the results of our Transwell assay showed the supernatants from primary RGCs undergoing more demise could attract more microglial and Müller cells. We propose that the cytokines released from injured RGCs promote the aggregation of microglial and Müller cells, which in turn aggravates inflammatory responses and RGC death. As either treatment of Fer-1, zVAD, Nec-1 or their combination was able to restrain the activation of phagocytes and reduce the elevation of inflammation mediators, all while attenuating the chemotaxis of phagocytes toward RGCs, we believe that the suppression of cell death pathways could interrupt this “doom loop” and thus facilitate the rescue of RGCs loss subsequent to retinal ischemia.

In this study, we only tested the protective effect of a single agent dose. As mice may benefit more from multiple doses [37], further research is needed to report the dose-response data and identify an ideal dosage. Additionally, given the difficulties with purifying and culturing the primary RGCs, as well as their fragile and low yielding property [38], we can only carry out some experiments in animal level, such as Western blots and TEM, the results of which may be biased by various other cellular populations present in the retina. Finally, we only examined early outcomes in the first 7 d after retinal IR and the neurologic function evaluation is lacking. The long-term effects of treatments and functional assessment still need to be addressed.

Altogether, our study of primary RGCs undergoing OGD/R and mice undergoing retinal IR injury demonstrated for the first time that the mechanistically distinct and independent modes of regulated cell death (apoptosis, necroptosis, and ferroptosis) coexist, interconnect, and weigh differently in their contribution to RGC loss. Crucially, the combined inhibition of multiple forms of cell death is more effective in promoting RGC survival after retinal IR injury. The elaborate uncovering of the molecular circadian mechanism relating to RGC death will be of vital importance to identify the optimal therapeutic methods for IR-induced neuron loss.

## Material and methods

### Animals and treatments

Male C57BL/6 mice (6–8 weeks old, body weight 19–24 g) were purchased from Charles River Laboratories (Beijing, China) and housed in a pathogen-free facility with free access to sterile acidified water and irradiated food. The retinal IR model was performed by acute elevation of intraocular pressure (IOP) via anterior chamber perfusion, during which a 33-gauge needle penetrated the cornea into the anterior chamber and its attached saline reservoir was elevated to obtain an IOP of 110 mmHg for 90 min. Removal of the needle allowed the release of pressure and natural reperfusion. Then, a topical antibiotic and steroid ointment (TobraDex; Alcon Laboratories, Inc., Texas, USA) was applied to the conjunctival sac. Eyes with cannulation-induced cataracts, iris/retinal bleeding, or anterior chamber leakage (leaks can be easily detected as dripping and wetting) were excluded [39, 40].

The mice were randomly allocated into six groups: (i) Normal Control group (NC); (ii) IR-Vehicle group (IR-Vehicle) mice were treated by anterior chamber perfusion and intravitreally injected with 2 μl 1% DMSO in normal saline on top of the IR insult; (iii) IR-Fer-1 group (IR-Fer-1); (iv) IR-zVAD group (IR-zVAD); (v) IR-Nec-1 group (IR-Nec-1), mice underwent similar surgical procedures and were injected with 2 μl 100 μM Fer-1 or zVAD or Nec-1 (MCE, New Jersey, USA) dissolved in normal saline with 1% DMSO, respectively; and vi) IR-Fer-1-zVAD-Nec-1 group (IR-FVN), after anterior chamber perfusion, the mice were intravitreally administered with a dose of 100 μM Fer-1, 100 μM zVAD, and 100 μM Nec-1 dissolved in 2 μl normal saline containing 1% DMSO collectively. Each drug dose (100 μM) was optimized by pilot experiments. Fer-1 (formula: C_15_H_22_N_2_O_2_), zVAD (formula: C_21_H_28_FN_3_O_7_), Nec-1 (formula: C_13_H_13_N_3_OS), RSL-3 (formula: C_23_H_21_ClN_2_O_5_), and Erastin (formula: C_30_H_31_ClN_4_O_4_) were all brought from MedChemExpress LLC, New Jersey, USA. They were originally dissolved in DMSO and then diluted to the target concentration with normal saline (Table S2).

All studies were conducted according to the NIH guidelines (Guide for the Care and Use of Laboratory Animals, 8th Edition, 2011) and approved by the Institutional Animal Care and Use Committee at the Second Affiliated Hospital of Zhejiang University, School of Medicine (No. AIRB-2021-499).

### Culture of primary RGCs and an OGD/R model

Primary RGCs were isolated from the retinas of postnatal day 5 (P5) mice. First, the retinas of P5 mice were dissected using a dissection microscope. Then, papain (16.5 Unit/mL) was used to dissociate the retina tissues into a single-cell suspension. Next, three-step panning was conducted for RGC purification [1]; a lectin-coated dish was used for negative panning, and the dish was shaken every 15 min (30 min in total) [2]; another lectin-coated dish was used for negative panning (10 min in total); and [3] mouse anti-mouse Thy1.2 antibody (Bio-Rad Laboratories, California, USA) was applied to the anti-mouse IgG + IgM (H + L) antibody (Jackson ImmunoResearch, Pennsylvania, USA)-coated dish for positive panning, and the dish was shaken every 15 min (45 min in total). After that, 30% fetal calf serum was pipetted directly to detach RGCs from the dish. Cells were then collected by centrifuging the tube at 1,000 rpm for 12 min at 25 °C, after which they were resuspended in a prewarmed RGC growth medium (neurobasal and equivalent high-glucose DMEM supplemented with NS21, Sato, L-glutamine, penicillin/streptomycin, N-acetyl-cysteine, insulin, sodium pyruvate, triiodothyronine (T3), forskolin, BDNF and CNTF). In total, about 20,000 cells/well were seeded in a 24-well plate containing coverslips coated with Poly-D-Lysine (Sigma-Aldrich, Missouri, USA) and mouse Laminin (R&D Systems, Minnesota, USA) for 2 h at 37 °C. The medium was changed every other day. RGC cultures were maintained in a humidified incubator at 37 °C and 10% CO_2_ for 5–7 d before treatment [41]. The cells were identified with Tuj-1 (Abcam, Cambridge, England). Control cells were incubated in DMEM with 4.5 g/l D-glucose (Thermo Fisher Scientific, Massachusetts, USA) at 37 °C in a humidified atmosphere of 5% CO_2_ and 95% air for 2.5 h. Experimental cells with DMEM (glucose-free) and 0.1% DMSO or 5, 10, or 25 μM Fer-1, zVAD, Nec-1, or FVN were placed in an airtight incubator that placed in a 37 °C incubator for 2.5 h, with a continuous flux of gas (95% N_2_/5% CO_2_). Cells were then re-oxygenated and placed into their complete medium with 0.1% DMSO or Fer-1, zVAD, Nec-1, or FVN for an additional 6 h before use.

### Western blot analysis

The control and experimental mice were anesthetized and sacrificed at different times over 7 d after IR. Retina tissues were collected and quantified by a Pierce™ BCA Protein Assay Kit (Thermo Fisher Scientific, Massachusetts, USA) after extraction using a Protein Extraction Kit (Solarbio, Beijing, China). Equal amounts of protein were electrophoresed on 10% SDS PAGE Gels (YaMei, Shanghai, China) and transferred to PVDF membranes (Millipore, New Jersey, USA). Membranes were then blocked with Protein Free Rapid Blocking Buffer (YaMei, Shanghai, China) and probed with primary antibodies (Table S2) at 4 °C overnight and secondary antibodies for 1 h at room temperature (RT). The blots were cut and separated into sections for different antibody probing. Protein expression was detected by Chemiluminescence imaging system (Bio-Rad, California, USA) and analyzed via densitometry with ImageLab software. To calculate the relative expression of a specific protein, the GAPDH served as a reference for the sample loading.

### Quantitative Real-time quantitative polymerase chain reaction (qRT-PCR)

qRT-PCR analysis was accomplished using gene-specific primers (Table S3). Specifically, total RNA was extracted from retinas using Trizol and reverse transcribed with the PrimeScript RT reagent Kit with gDNA Eraser (Takara, Kyoto, Japan) to synthesize cDNA. qRT-PCR was then performed in the Applied Biosystems™ 7500 (Thermo Fisher Scientific, Massachusetts, USA) using the SYBR PrimeScript Plus RT-PCR Kit (Takara, Kyoto, Japan). For each gene, relative expression was calculated by comparison with a standard curve, following normalization to the housekeeping gene GAPDH expression.

### Immunofluorescent and HE staining for histological examination

Fixed retinas were sectioned to a thickness of 8 μm (frozen for immunofluorescence staining) through the disc with a vibratome (Leica Microsystems, Exton, Panama). The frozen sections were permeabilized with 0.3% Triton X-100 in phosphate buffer saline (PBS) for 1 h, rinsed three times in PBS, blocked in buffer (5% goat serum, 2% BSA, and 0.1% Tween-20 in PBS) for 1 h, and incubated overnight with primary antibodies against NeuN, p-S6, GFAP, Iba-1, IB4 Isolectin and Tuj-1 antibodies (Table S2) at 4 °C overnight, followed by species-specific secondary fluorescent antibodies. After the three PBS washes, we applied coverslips to the sections with DAPI Fluoromount-G™ (Yeasen Biotech, Shanghai, China) before imaging with a Leica DMi8 microscope (Leica Microsystems, Exton, Panama).

5-μm-thick paraffin sections were cut through the optic nerve and stained with HE, and the retinal histoarchitecture was evaluated through light microscopy. For the quantification of RGC number, four standard areas (200 × 200 μm^2^) of each section at a point 1 mm from the optic disc were randomly chosen. Cells were counted by two observers blinded to the identity of the mice, and the average number of RGCs was calculated from at least four independent biological replicates [42, 43]. The IPL thickness was also measured in four adjacent areas within 1 mm of the optic disc. ImageJ software (National Institutes of Health, Bethesda, MD) was used to label the thickness [44].

### GPx activity, GSH/GSSG, and MDA assay

The GPx activity was determined by the Glutathione Peroxidase (GPx) Activity Assay Kit (Sangon Biotech, Shanghai, China), according to the manufacturer’s recommendations. The GSH and GSSG levels in the retina tissue were quantified by a GSH and GSSG Assay Kit (Beyotime, Jiangsu, China), according to the manufacturer’s protocols. The relative MDA concentration in the retina tissue lysates was assessed using a lipid peroxidation assay kit (Abcam, Cambridge, England) according to the manufacturer’s instructions.

### TUNEL assay

Apoptotic cells were detected by terminal deoxynucleotidyl transferase (TdT)-mediated dUTP nick end labeling (TUNEL). Frozen sections of 8 μm thickness were probed with One-Step TUNEL Apoptosis Assay Kit (Beyotime, Jiangsu, China). Finally, the sections were stained with DAPI and observed under a Leica DMi8 microscope, so TUNEL positive and DAPI positive cells could be easily observed.

### TEM

After fixing in 2.5% glutaraldehyde in a 0.1 M phosphate buffer for at least 2 h, mouse or human retinas were fixed with 1% OsO_4_ and washed, followed by dehydration with graded alcohol and embedding in acetone, acetone with 33% epoxy resin, acetone with 67% epoxy resin, and epoxy resin successively. Ultrathin sections were placed on copper grids, stained with uranyl acetate and lead citrate, and examined under a 120 kV electron microscope (Tecnai G2 Spirit, FEI, Massachusetts, USA). Frequency of the distribution of the mitochondrial area was calculated with ImageJ software.

### Detection of cell viability

Cells were stained with Calcein AM (2 μM) and Ethidium homodimer-1 (4 μM; Live & Dead™ Viability/Cytotoxicity Assay Kit for Animal Cells, US Everbright, Suzhou, China) for 15 min, and imaging was performed at random sites with a Leica DMi8 microscope. Average neurite outgrowth refers to the average length of all neurites that arose from the cell body, whereas axonal outgrowth is defined as the length of the longest neurite. The lengths of the axon and other neurites of the RGCs were measured semi-automatically through the Simple Neurite Tracer plugin in ImageJ software [45]. The investigators measuring the neurites were masked to the identity of the treatments in all experiments.

### Flow cytometry analysis of intracellular lipid peroxides

Primary RGCs undergoing OGD/R were incubated in dark with C11 BODIPY (Thermo Fisher Scientific, Massachusetts, USA) for at least 30 min during reoxygenation and were applied to flow cytometry immediately after digestion, centrifugation, and resuspension to 10^6^/mL. We used a 488-nm laser on the flow cytometer (BD Canto II, New Jersey, USA) and set up a dot plot to detect size (forward scatter) and granularity (side scatter) using a linear scale. We also set up dot plots to detect BODIPY (585 and 530 nm). The cytometric results were analyzed using FlowJo 7.6.1 software (FlowJo, LLC, Oregon, USA) according to standard procedures. Histograms of BODIPY in log scale were used to illustrate the data visually.

### Immunocytochemistry

We used 4% paraformaldehyde to fix the RGCs for 15 min at RT, PBS to wash the samples three times, blocking buffer with 5% normal goat serum, and 0.1% Triton X-100 to block unspecific targets for 30 min at RT. Anti-Tuj-1 antibodies were diluted in PBS with 1% normal goat serum and 0.1% Triton X-100 and incubated with the RGCs overnight at 4 °C. Secondary antibodies were diluted and applied to RGCs. After incubating for 1 h at RT in dark and washing three times by PBS, we applied coverslips to the glass slides with DAPI before imaging.

### Fe^2+^ probing, ROS detection, and analysis of cell apoptosis and necrosis in vitro

FerroOrange (Dojindo, Kyushu, Japan) and Dihydroethidium (Beyotime, Jiangsu, China) were used to detect intracellular Fe^2+^ and ROS levels according to the manufacturer’s protocols. For analysis of cell apoptosis and necrosis, primary RGCs were incubated in dark with Annexin V-FITC for 15 min and then with PI (Sangon, Shanghai, China). The stained cells were then analyzed under a Leica DMi8 microscope to differentiate viable (Annexin V−/PI−), early apoptotic (Annexin V+/PI−), late apoptotic (Annexin V+/PI+), and necrotic cells (Annexin V−/PI+) [46].

### Transwell migration assay

BV2 or Müller cells (1 × 10^4^) were seeded onto Transwell with a polyethylene terephthalate membrane pore size of 8 μm in 24-well plates [47]. For BV2, the mixture of DMEM (high glucose, 2% FCS) and supernatants from normal RGCs (1:1) was added to the inner Transwell, while the mixture of DMEM (high glucose, 5% FCS) and supernatants from RGCs in different experimental groups (1:1) was added to the outer Transwell. For the Müller cells, the mixture of DMEM (low glucose, 1.5% FCS) and supernatants from normal RGCs (1:1) was added to the inner Transwell, while the mixture of DMEM (low glucose, 3% FCS) and supernatants from RGCs in different experimental groups (1:1) was added to the outer Transwell. In particular, media for BV2 in the Transwell assay were added with dextrose for a final concentration up to 4.9 g/L to compensate the minor loss in the RGCs’ medium during the 6 h reoxygenation. After 24 h, the media within the Transwell inserts were carefully removed. The cells were fixed with 4% paraformaldehyde, washed with PBS, and stained with crystal violet (Beyotime, Jiangsu, China). Cells that did not migrate across the Transwell membrane were removed by gently wiping them away with a cotton swab. Migrated cells were viewed with a Leica DMi8 microscope under bright field. To exclude the direct effect from DMSO or the inhibitors mentioned above, we also seeded the Müller glia, which had undergone 2 h OGD and 6 h reoxygenation, onto the Transwell with 0.1% DMSO, Fer-1, zVAD, Nec-1, or FVN out of the inserts and inner/outer FCS gradient was 0.5%/1%. The cell lines were tested to be free of mycoplasma contamination and were authenticated via STR profiling.

### Immunohistochemistry

After deparaffinization by xylene and antigen retrieval by 10 mM citrate buffer (pH 6.0), the retina sections were immersed in 3% H_2_O_2_ for 10 min at RT and blocked with normal goat serum solution at RT for 1 h. Then, the primary antibodies (RIP1, Caspase 3, GPX4, and FSP1; Table S2) were applied and the sections incubated at 4 °C overnight. Subsequently, the sections were washed with PBS and incubated with horseradish peroxidase–conjugated goat anti-rabbit IgG polyclonal antibodies at RT for 30 min. The sections were stained with 3,3’-diaminobenzidine for 10 s and counterstained with hematoxylin for 20 s. Finally, after sealing with neutral gum, all slides were evaluated with a microscope (Leica DM2500).

### Human eye tissue

The human retinas were collected from end-stage glaucoma patients and a deceased healthy donor at the time of eye enucleation. Retinas were stored in a sterile balanced salt solution at 4 °C for later analysis of HE staining, immunofluorescent staining, TEM and immunohistochemistry. All human retina tissues were obtained from the Department of Ophthalmology, the Second Affiliated Hospital of Zhejiang University from February 2021 to September 2021. This work has been carried out in accordance with The Code of Ethics of the World Medical Association (Declaration of Helsinki) for experiments involving humans and obtained the approval of the Human Research Ethics Committee at the Second Affiliated Hospital of Zhejiang University, School of Medicine (No. 120211118). Signed informed consents were obtained from all participants or their clients.

### Statistics

All data were reported as mean ± standard deviation (SD) unless indicated otherwise and *p* < 0.05 was considered statistically significant. All experiments were carried out at least three times unless otherwise stated. The statistical details (such as statistical tests used) and repeated times of the experiments are given in the figure legends. Comparisons between multiple groups were assessed by GraphPad Prism 8.0 using one-way analysis of variance (ANOVA), followed by Bonferroni post hoc test.

## Supplementary information

supplemental materials

Original Data File

checklist

## Data Availability

All data generated or analyzed during this study are included in this published article and its Supplementary files and available from the corresponding authors on request.
