# Supplementary material for: Inhibiting multiple forms of cell death optimizes ganglion cells survival after retinal ischemia reperfusion injury
Source: Cell Death Dis. 2022 May 30;13(5):507. doi: 10.1038/s41419-022-04911-9 (PMC9151775; doi:10.1038/s41419-022-04911-9)
Supplement: Supplementary file 1 — supplemental materials [file 41419_2022_4911_MOESM1_ESM.docx]

**Supplementary information for**

**Inhibiting multiple forms of cell death optimizes [ganglion](javascript:;) [cell](javascript:;)s survival after retinal ischemia reperfusion injury**

**This file includes：**

Supplementary Figure S1 to S8

Supplementary Table S1 to S3

**
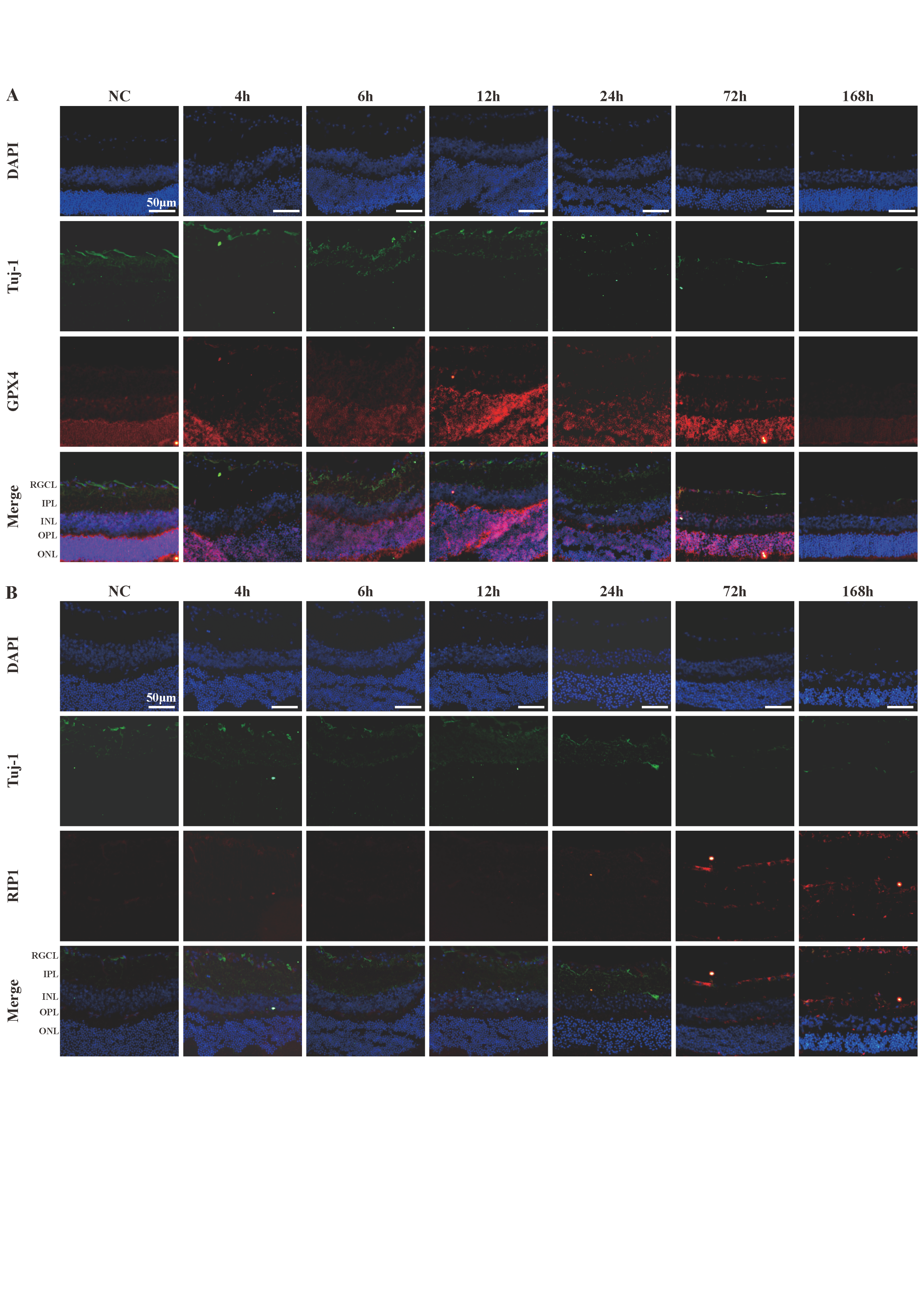
**

**Fig. S1 Expression change of GPX4 and RIP1 in RGCs from normal or IR-injured retinas.** (A) Representative images of Tuj-1 (green) and GPX4 (red) in normal or IR-injured retinas at indicated times after IR-injury. (B) Representative images of Tuj-1 (green) and RIP1 (red) in normal or IR-injured retinas at indicated times after IR injury. Nucleus was marked with DAPI (blue). Scale bar = 50 μm. NC: normal control; IR: ischemia reperfusion; RGCL: retinal ganglion cell layer; IPL: inner plexiform layer; INL: inner nuclear layer; OPL: outer plexiform layer; ONL: outer nuclear layer.

##
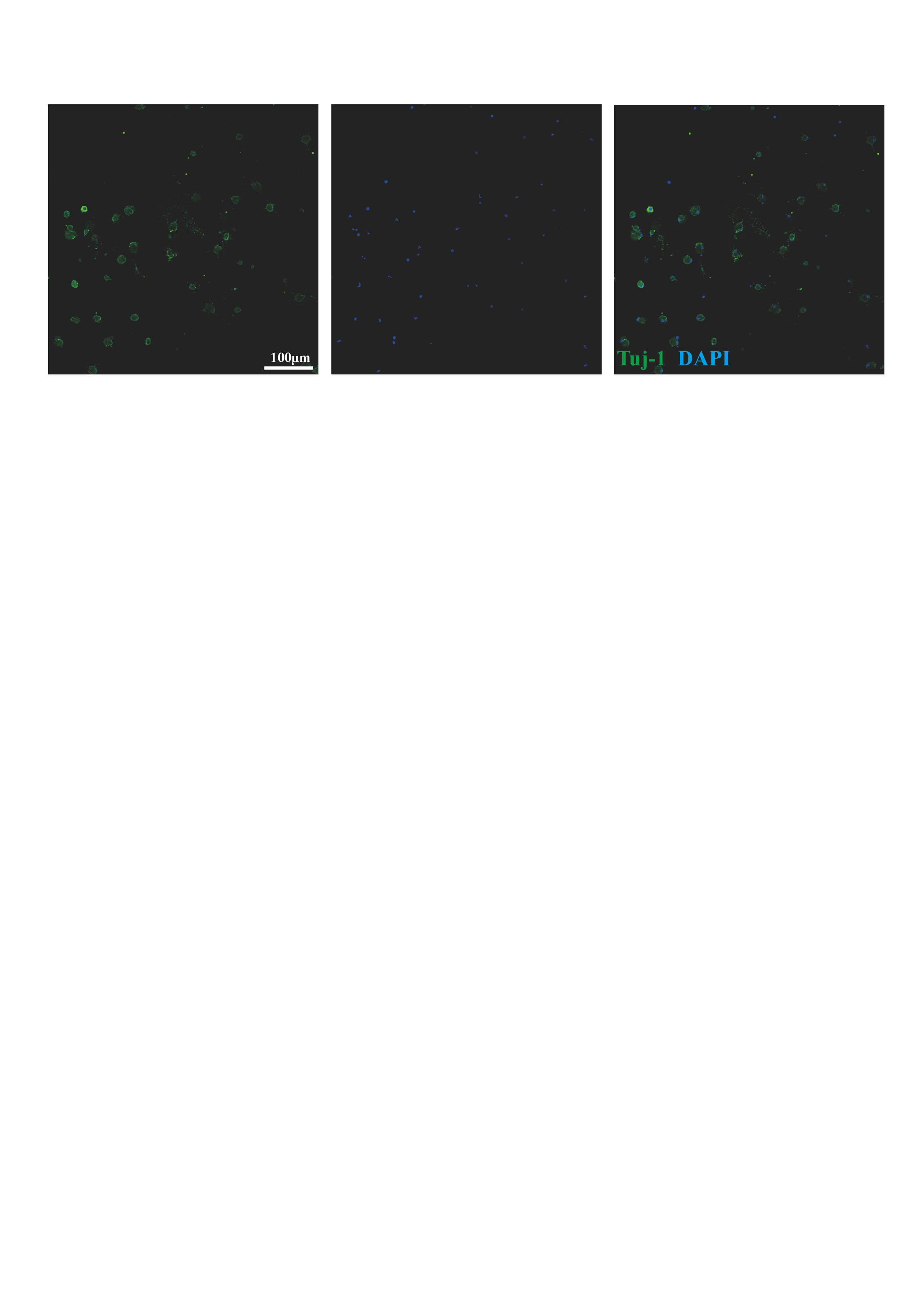


## Fig. S2 Identification of primary RGCs. Cultured primary RGCs were identified by immunofluorescent staining with anti-Tuj-1 antibody (green) and nucleus was marked with DAPI (blue) on the first day after isolation. Scale bar = 100 µm.


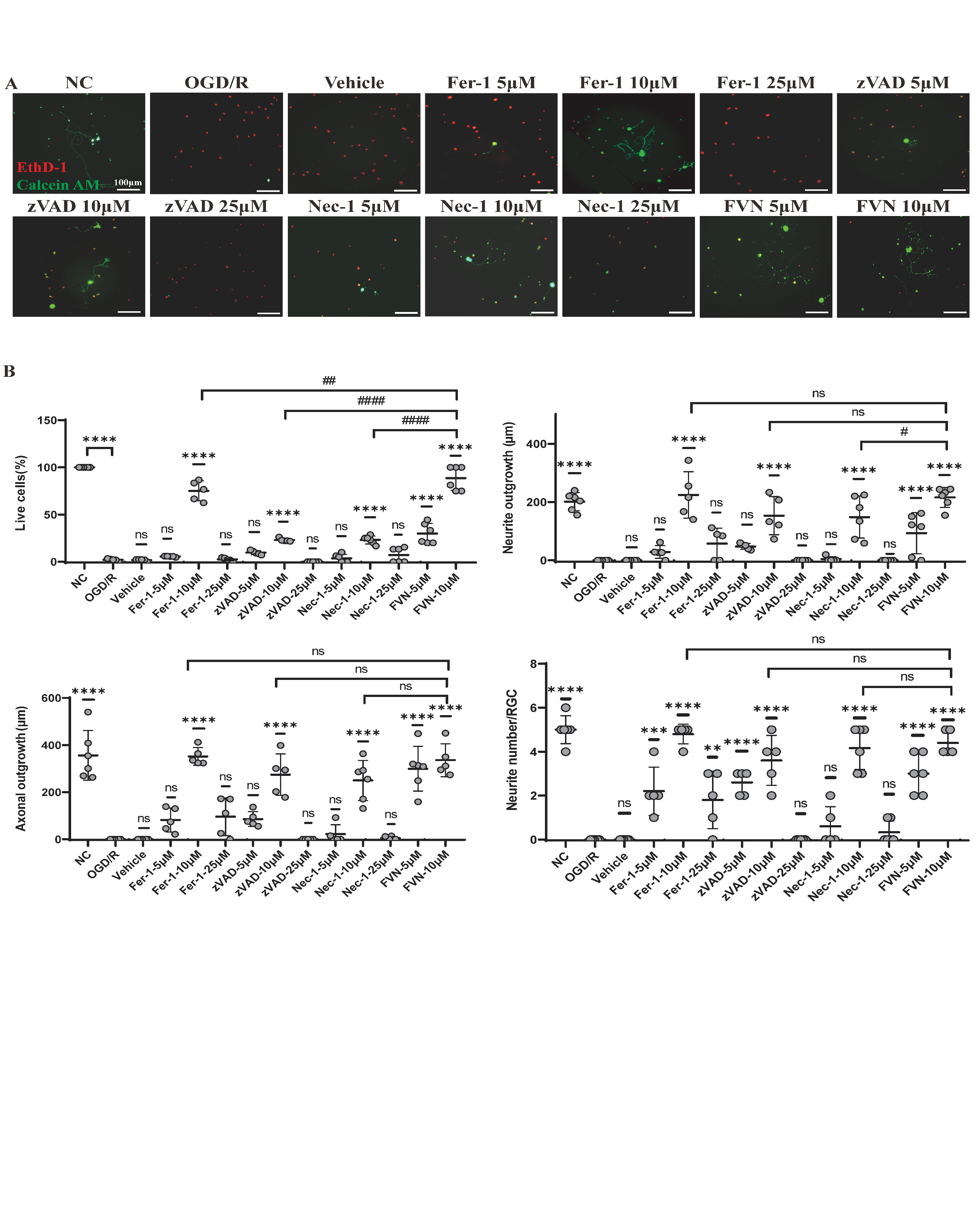


**Fig. S3 Effect of different concentrations of Fer-1, zVAD, Nec-1 and FVN on OGD/R-induced cell injury in** **primary RGCs.** (A) Live/Dead assay staining for mouse primary RGCs that underwent OGD/R with treatment of vehicle (DMSO) or 5, 10, 25 μM Fer-1, zVAD, Nec-1 and 5, 10 μM FVN (green for live cells/red for dead cells). Scale bar = 100 μm. (B) Statistical analysis on percentage of live cells, neurite outgrowth, axonal outgrowth and average neurite number (n = 6). Data are represented as the mean ± SD. **p* < 0.05, ***p* < 0.01, ****p* < 0.001, *****p* < 0.0001 versus vehicle; *^#^p* < 0.05, ^##^*p* < 0.01, ^###^*p* < 0.001, ^####^*p* < 0.0001 versus FVN; one-way ANOVA with Bonferroni post hoc analysis. NC: normal control; OGD/R: oxygen glucose deprivation/reoxygenation; FVN: the combination of Fer-1, zVAD, and Nec-1.

**
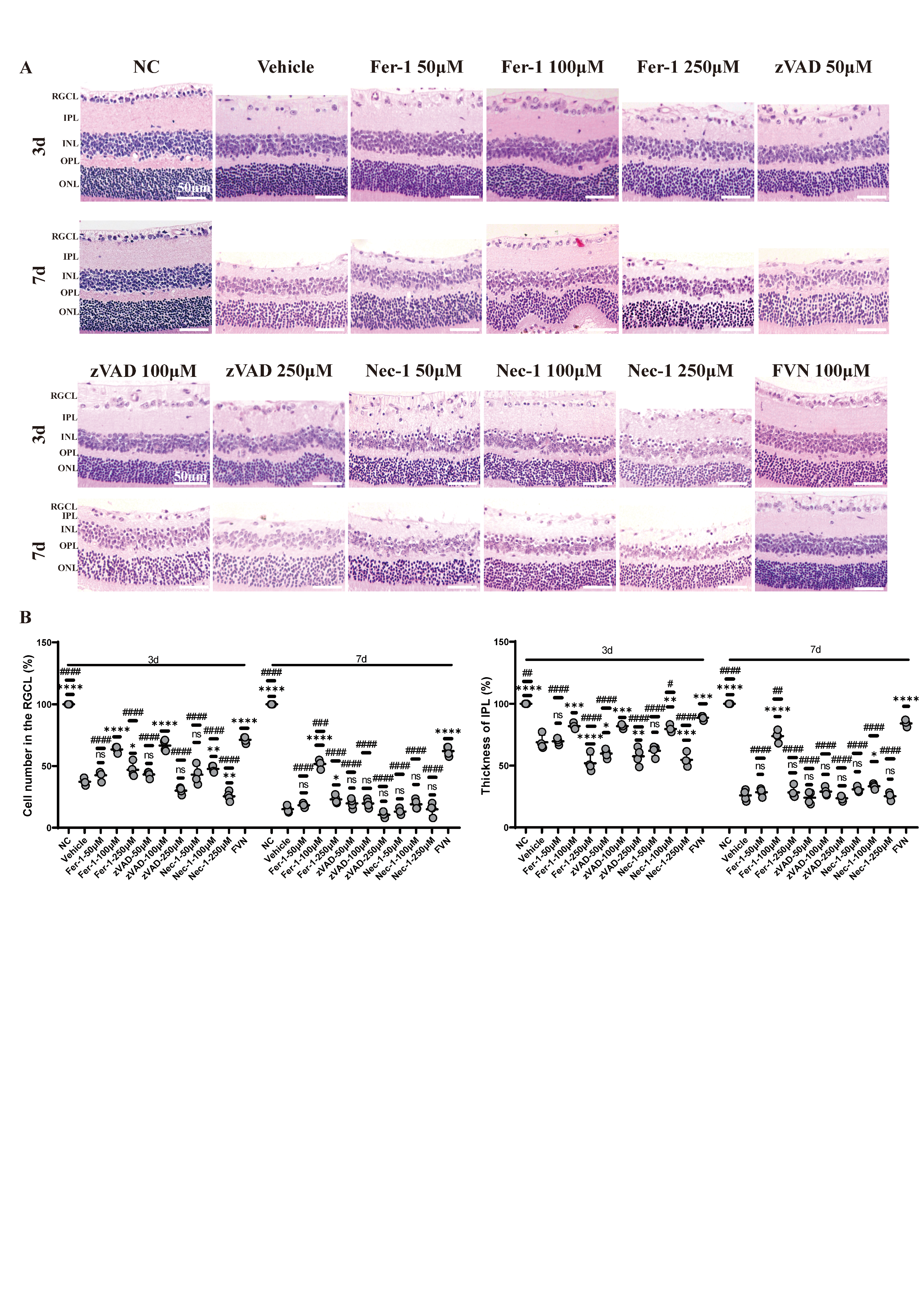
**

**Fig. S4 Effect of different concentrations of Fer-1, zVAD, Nec-1 and FVN on IR-induced retina injury in mice.** (A) HE staining of retinal tissue in mice underwent sham or IR injury at 3 and 7 d after intravitreal injection of vehicle (DMSO) or 50, 100, 250 μM Fer-1, zVAD, Nec-1 and 100 μM FVN. (B) Analysis on RGCs number and IPL thickness (n = 4). Scale bar = 50 μm. Data are represented as the mean ± SD. ^*^*p* < 0.05, ^**^*p* < 0.01, ^***^*p* < 0.001, ^****^*p* < 0.0001 versus vehicle; ^#^*p* < 0.05, ^##^*p* < 0.01, ^###^*p* < 0.001, ^####^*p* < 0.0001 versus FVN; one-way ANOVA with Bonferroni post hoc analysis. NC: normal control; IR: ischemia reperfusion; RGCL: retinal ganglion cell layer; IPL: inner plexiform layer; INL: inner nuclear layer; OPL: outer plexiform layer; ONL: outer nuclear layer; FVN: the combination of Fer-1, zVAD, and Nec-1.


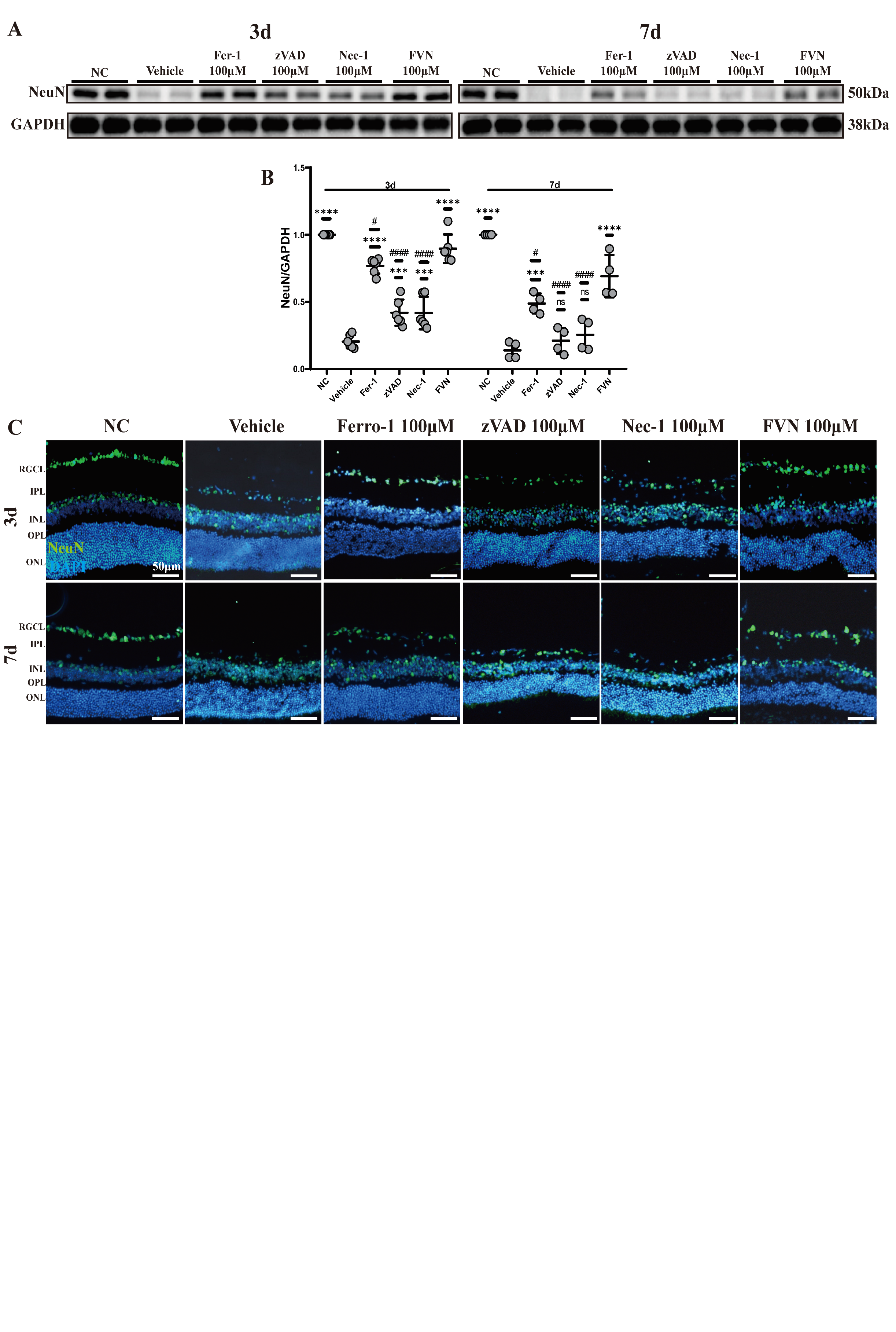


**Fig. S5 In situ administration of 100 μM Fer-1, zVAD, Nec-1 and FVN alters NeuN expression in mice retina underwent IR injury.** (A) Western blot bands of NeuN and GAPDH in sham or IR-injured retina 3 and 7 d after each treatment. (B) Quantitative analysis of the protein expression levels of NeuN (n = 4-6). Data are represented as the mean ± SD. ^*^*p* < 0.05, ^**^*p* < 0.01, ^***^*p* < 0.001, ^****^*p* < 0.0001 versus vehicle; ^#^*p* < 0.05, ^##^*p* < 0.01, ^###^*p* < 0.001, ^####^*p* < 0.0001 versus FVN; one-way ANOVA with Bonferroni post hoc analysis. (C) Representative images of neurons in sham or IR-injured retina 3 and 7 d after each treatment. Neurons were marked with NeuN (green). Nucleus was marked with DAPI (blue). Scale bar = 50 μm. NC: normal control; IR: ischemia reperfusion; RGCL: retinal ganglion cell layer; IPL: inner plexiform layer; INL: inner nuclear layer; OPL: outer plexiform layer; ONL: outer nuclear layer; FVN: the combination of Fer-1, zVAD, and Nec-1.


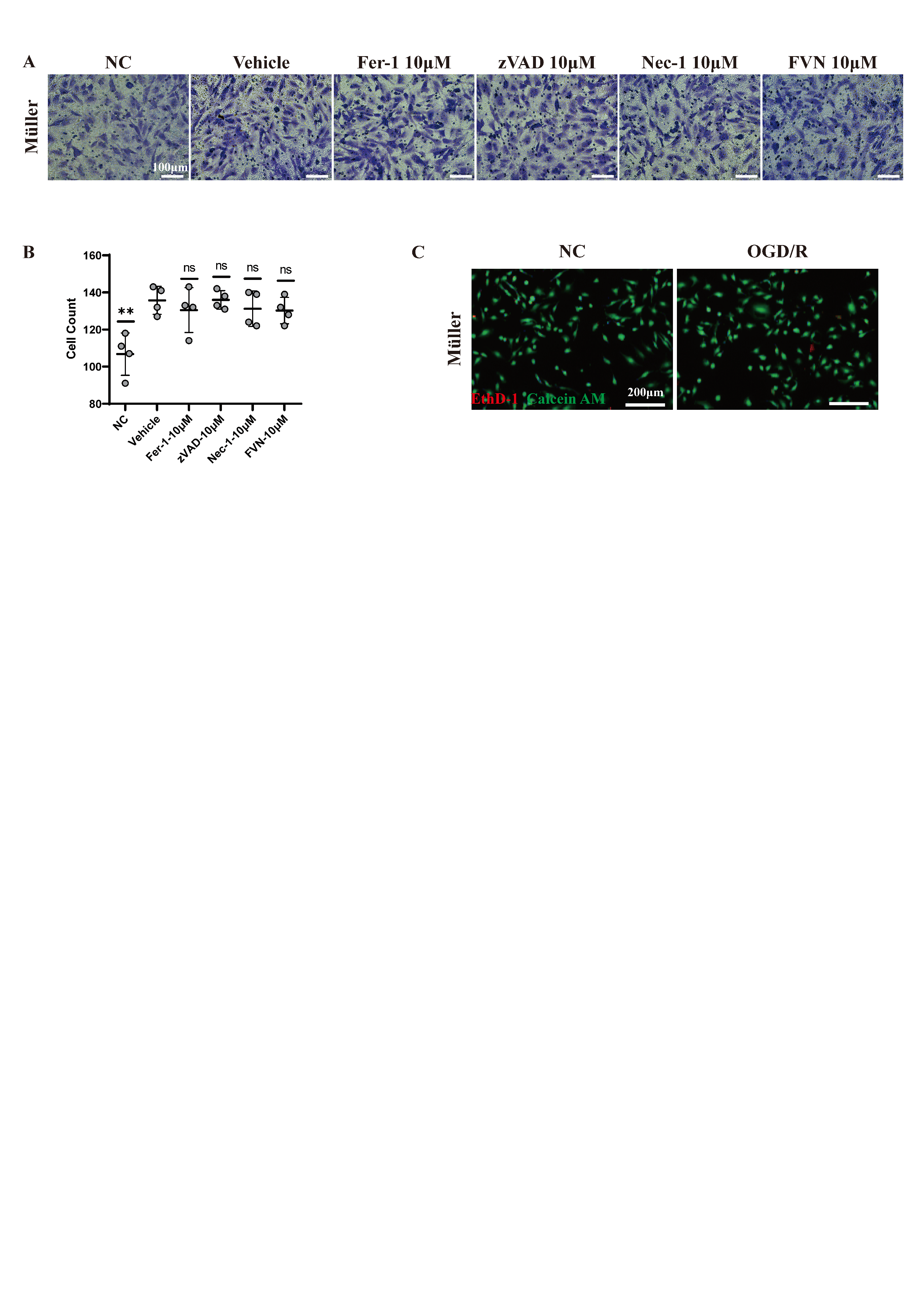


**Fig. S6 10 μM Fer-1, zVAD, Nec-1 and FVN have no direct impact on Müller cell line’s survival and chemotaxis.** (A) The chemotaxis of 10 μM Fer-1, zVAD, Nec-1 and FVN toward Müller cell lines was tested by 24 h Transwell assay and following crystal violet staining and (B) statistical analysis on cell number was performed (n = 4). Scale bar = 100 μm. Data are represented as the mean ± SD. ^**^*p* < 0.01 versus vehicle; one-way ANOVA with Bonferroni post hoc analysis. (C) Representative Live/Dead assay staining for Müller cell lines underwent 2 h OGD and 6 h reoxygenation (n = 3). Scale bar = 200 μm. NC: normal control; OGD/R: oxygen glucose deprivation/reoxygenation; FVN: the combination of Fer-1, zVAD, and Nec-1.


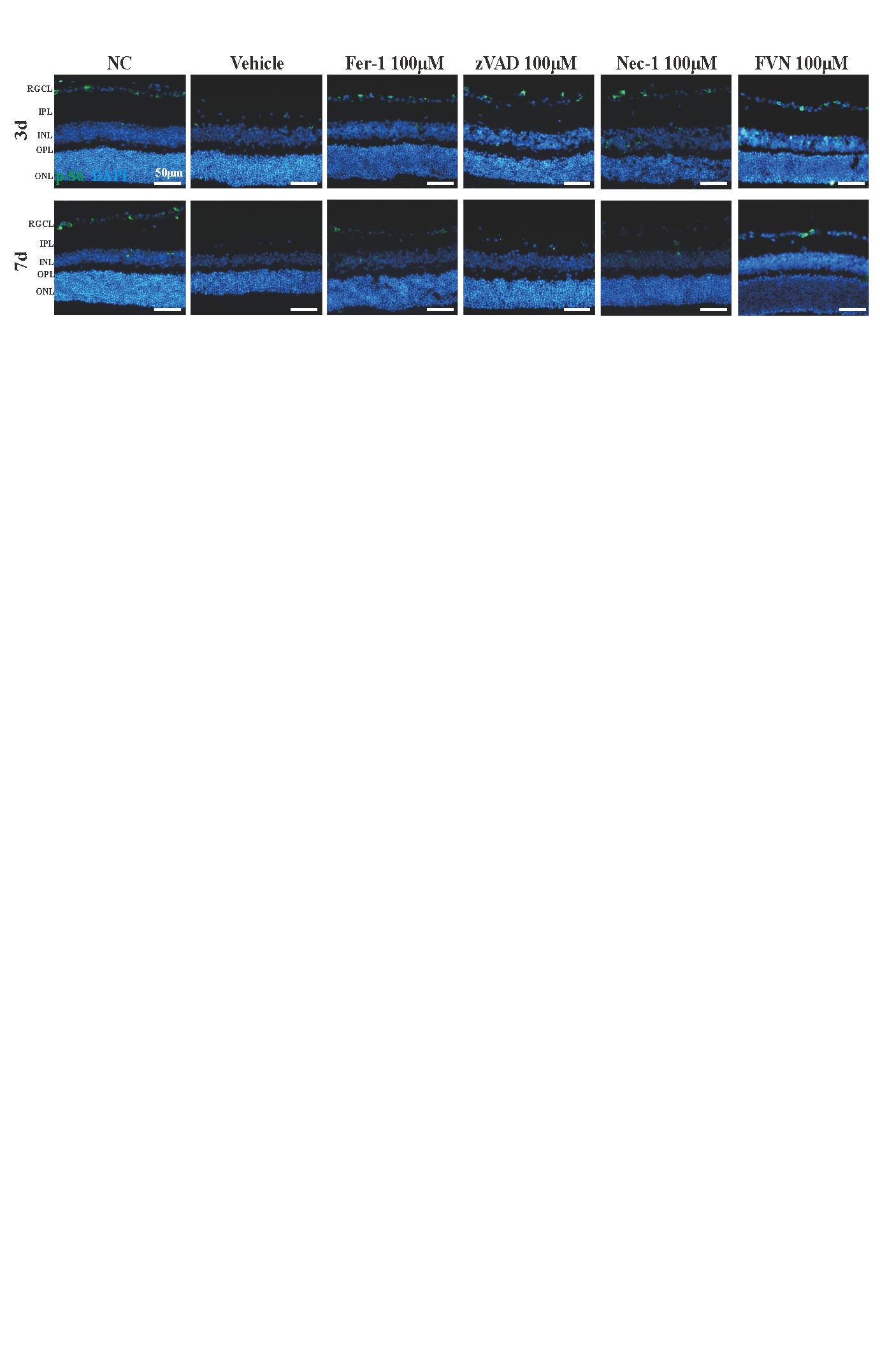


**Fig. S7 In situ administration of 100 μM Fer-1, zVAD, Nec-1 and FVN alters p-S6 expression in mice retina underwent IR injury.** Representative images of p-S6 (green) expression in sham or IR-injured retina 3 and 7 d after each treatment. Nucleus was marked with DAPI (blue). Scale bar = 50 μm. NC: normal control; IR: ischemia reperfusion; RGCL: retinal ganglion cell layer; IPL: inner plexiform layer; INL: inner nuclear layer; OPL: outer plexiform layer; ONL: outer nuclear layer; FVN: the combination of Fer-1, zVAD, and Nec-1.


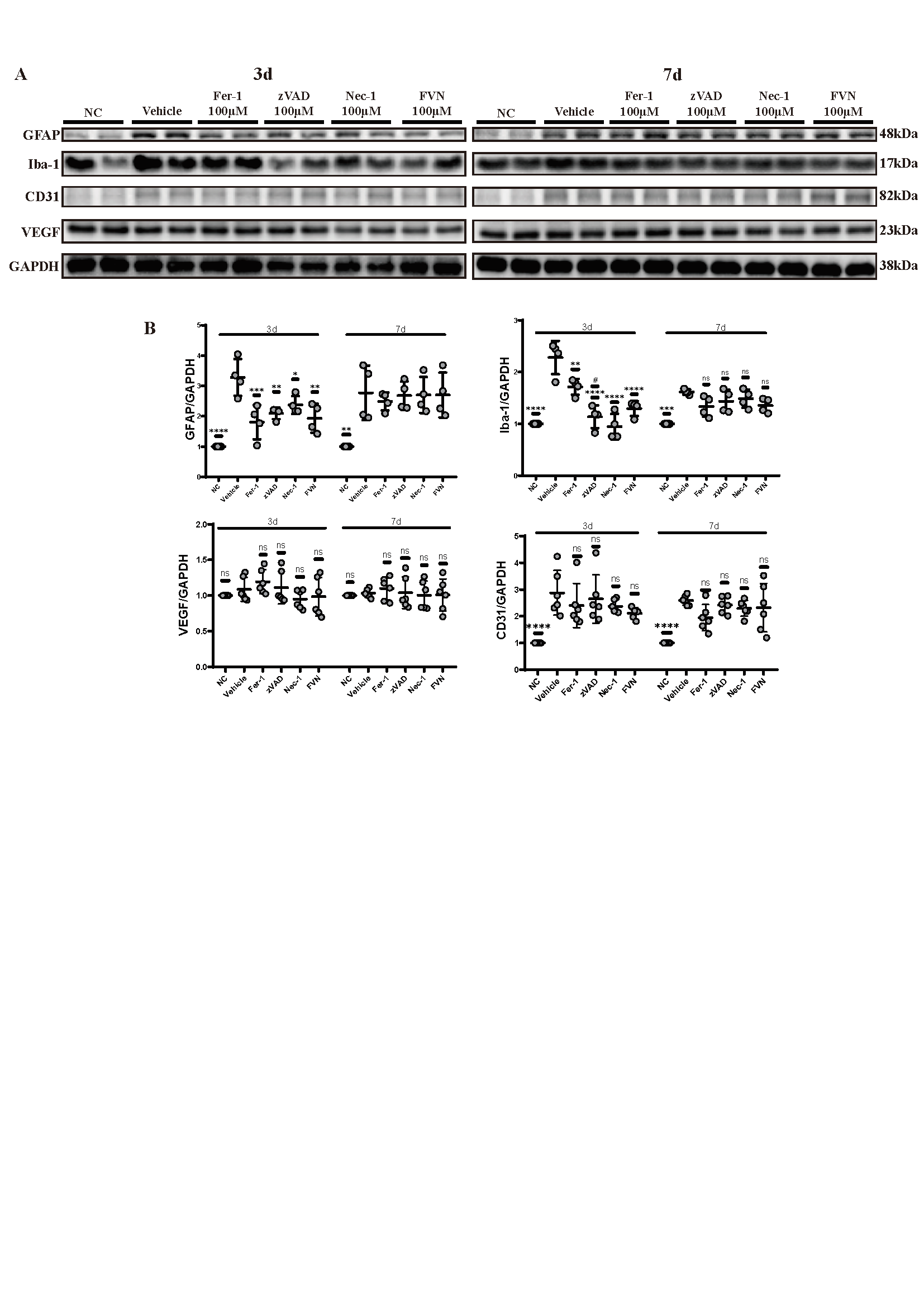


**Fig. S8 Expression level of GFAP, Iba-1, CD31 and VEGF in sham and IR-injured retina at 3 and 7 d post 100 μM Fer-1, zVAD, Nec-1 and FVN treatment.** (A) Western blot bands of GFAP, Iba-1, CD31, VEGF and GAPDH in sham or IR-injured retina 3 and 7 d after each treatment and (B) quantitative analysis of the protein expression levels (n = 4-6). FVN: the combination of Fer-1, zVAD and Nec-1. Data are represented as the mean ± SD. ^*^*p* < 0.05, ^**^ *p* < 0.01, ^***^*p* < 0.001, ^****^*p* < 0.0001 versus vehicle; ^#^*p* < 0.05, ^##^*p* < 0.01, ^###^*p* < 0.001, ^####^*p* < 0.0001 versus FVN; one-way ANOVA with Bonferroni post hoc analysis. NC: normal control; IR: ischemia reperfusion; FVN: the combination of Fer-1, zVAD, and Nec-1.

**Table S1. Clinical information of the healthy donor and patients with end-stage glaucoma.**

| **Patient type** | **Gender** | **Age** | **Glaucoma Course (year)** | **Visual Acuity** | **Latest IOP**  **(mmHg)*** |
| --- | --- | --- | --- | --- | --- |
| Healthy Donor | Male | 25 | 0 | N/A | 17.0 |
| Congenital Glaucoma | Female | 18 | 12 | NLP | 40.7 |
| Primary Angle closure Glaucoma | Female | 69 | 7 | NLP | 53.0 |
| Neovascular Glaucoma | Male | 45 | 5 | NLP | 55.0 |

IOP: intraocular pressure. *Normal range: 10–21 mmHg. NLP: No Light Perception. N/A: Not Applicable

**Table S2. Inhibitors, activators, and antibodies used for medication, immunofluorescent staining and immunohistochemistry, and Western blot.**

| **Inhibitors** | **Source** | **Company** | **CAS No.** | **Dilution^1^** |
| --- | --- | --- | --- | --- |
| Fer-1^2^ | Chemical Compound | MedChemExpress | 347174-05-4 | 1:100 in NS^2^ |
| zVAD^2^ | Chemical Compound | MedChemExpress | 161401-82-7 | 1:100 in NS^2^ |
| Nec-1^2^ | Chemical Compound | MedChemExpress | 4311-88-0 | 1:100 in NS^2^ |
| **Inducers** | **Source** | **Company** | **CAS No.** | **Dilution^1^** |
| RSL-3^2^ | Chemical Compound | MedChemExpress | [1219810-16-8](https://www.medchemexpress.cn/search.html?q=1219810-16-8&ft=&fa=&fp=) | 1:2000 in NS^2^ |
| Erastin^2^ | Chemical Compound | MedChemExpress | [571203-78-6](https://www.medchemexpress.cn/search.html?q=571203-78-6&ft=&fa=&fp=) | 1:200 in NS^2^ |
| **Primary Antibodies** | **Source** | **Company** | **Catalog No.** | **Dilution^1^** |
| TF | Rabbit mAb | Abcam | ab278498 | 1:1000 |
| TFR1 | Rabbit pAb | ABclonal | 5865 | 1:1000 |
| SLC7a11 (xCT) | Rabbit mAb | Abcam | ab175186 | 1:5000 |
| VDAC | Goat mAb | Abcam | ab37985 | 1μg/mL |
| ACSL4 | Rabbit mAb | Abcam | ab155282 | 1:20000 |
| GPX4 | Rabbit pAb | Abcam | ab125066 | 1:2000  1:200 IHC-P |
| FSP1 | Rabbit pAb | Proteintech | 20886-1 | 1:1000,  1:200 IHC-P |
| Caspase 9 | Rabbit mAb | Abcam | ab202068 | 1:2000 |
| Cleaved Caspase 3 | Rabbit mAb | Abcam | ab214430 | 1:1000 |
| Bcl2 | Rabbit mAb | Abcam | ab182858 | 1:2000 |
| Pro-Caspase 8 | Rabbit mAb | Abcam | ab108333 | 1:5000  1:200 IHC-P |
| RIP1 | Rabbit mAb | Abcam | ab202985 | 1:1000 |
| RIP3 (phospho S232) | Rabbit mAb | Abcam | ab195117 | 1:1000 |
| Iba-1 | Rabbit mAb | Abcam | ab178846 | 1:1000  1:500 IF |
| GFAP | Rabbit mAb | Abcam | ab68428 | 1:10000  1:250 IF |
| CD31 | Rabbit mAb | Abcam | ab222783 | 1:2000 |
| VEGF | Rabbit pAb | ABclonal | A12303 | 1:1000 |
| Tuj-1 (beta III Tubulin) | Rabbit pAb | Abcam | ab18207 | 1 µg/mL |
| Tuj-1 (beta III Tubulin) | Mouse pAb | Abcam | ab78078 | 1 µg/mL |
| NeuN | Rabbit mAb | Abcam | ab177487 | 1:1000 |
| IB4 isolectin | Africa Shrub legume | Thermo | I21412 | 1:500 IF |
| Phospho-S6 (Ser235/236) | Rabbit mAb | Cell Signaling Technology | 4858T | 1:100 IF |
| GAP43 | Rabbit mAb | Abcam | ab75810 | 1:1000 |
| Cox2 | Rabbit mAb | Abcam | ab179800 | 1:2000 |
| GAPDH | Mouse mAb | Proteintech | ab9482 | 1:10000 |
| Caspase 3 | Rabbit pAb | Abcam | ab32351 | 1:50 IHC-P |
| RIPK1 | Rabbit pAb | ABclonal | 4814 | 1:100 IHC-P |
| **Secondary Antibodies** | **Source** | **Company** | **Catalog No.** | **Dilution^1^** |
| Goat Anti-Rabbit IgG (H&L) (HRP) | Goat pAb | Proteintech | SA00001-15 | 1:10000 |
| Rabbit anti-Goat IgG (H&L) (HRP) | Rabbit pAb | Proteintech | SA00001-4 | 1:10000 |
| Goat anti-Rabbit IgG (H&L) (Alexa Fluor® 555) | Goat pAb | Cell Signaling Technology | 4413 | 1:1000 IF |
| Goat anti-Rabbit IgG (H&L) (Alexa Fluor® 488) | Goat pAb | Cell Signaling Technology | 4412 | 1:1000 IF |
| Goat anti-Rabbit IgG (H&L) (HRP) | Goat pAb | Solarbio | SE134 | 1:200 IHC-P |
| Goat anti-mouse IgG (H&L) (Alexa Fluor® 488) | Goat pAb | Cell Signaling Technology | 4408S | 1:1000 IF |

^1^The dilution ratio of antibodies without following IF or IHC-P was used for the Western blot.

^2^Fer-1: Ferrostatin-1 (10mM in DMSO); zVAD: z-VAD-FMK (10mM in DMSO); Nec-1: Necrostatin-1 (10mM in DMSO); RSL-3 (10mM in DMSO); Erastin (10mM in DMSO); NS: Normal Saline.

**Table S3. Gene-specific primers (5’-3’, F = Forward, R = Reverse).**

| **Gene** | **Primer** |
| --- | --- |
| Atp5g3-F | TCTGCATCAGTGTTATCTCGGC |
| Atp5g3-R | CACCAGAACCAGCAACTCCTA |
| Rpl8-F | CGGGAACTACGCCACAGTC |
| Rpl8-R | CACGGCCAGCCTTTAAGATAG |
| Cs-F | GGACAATTTTCCAACCAATCTGC |
| Cs-R | TCGGTTCATTCCCTCTGCATA |
| Ireb2-F | CGGCACCAAGTATGATATTCTGC |
| Ireb2-R | AGGGCACTTCAACATTGCTCT |
| Ptgs2-F | TGAGCAACTATTCCAAACCAGC |
| Ptgs2-R | GCACGTAGTCTTCGATCACTATC |
| GAPDH-F | AGGTCGGTGTGAACGGATTTG |
| GAPDH-R | TGTAGACCATGTAGTTGAGGTCA |
| TNF-α-F | CCTGTAGCCCACGTCGTAG |
| TNF-α-R | GGGAGTAGACAAGGTACAACCC |
| IL-1β-F | GAAATGCCACCTTTTGACAGTG |
| IL-1β-R | TGGATGCTCTCATCAGGACAG |
| IL-6-F | CTGCAAGAGACTTCCATCCAG |
| IL-6-R | AGTGGTATAGACAGGTCTGTTGG |
| NF-κB-F | GGAGGCATGTTCGGTAGTGG |
| NF-κB-R | CCCTGCGTTGGATTTCGTG |
| iNOS-F | TCCTGGAGGAAGTGGGCCGAAG |
| iNOS-R | CCTCCACGGGCCCGGTACTC |
| CCL2-F | TTAAAAACCTGGATCGGAACCAA |
| CCL2-R | GCATTAGCTTCAGATTTACGGGT |
