# Supplementary material for: Inhibiting multiple forms of cell death optimizes ganglion cells survival after retinal ischemia reperfusion injury
Source: Cell Death Dis. 2022 May 30;13(5):507. doi: 10.1038/s41419-022-04911-9 (PMC9151775; doi:10.1038/s41419-022-04911-9)

Dear Editor:

All original images of the Western blots in both formal and supplementary figures were provided here. For the original images which had one molecular weight marker blurred in the Merge mode, we provided the corresponding Colorimetric pictures, the adjacent blots or full images of the whole membrane to prove the authenticity and reliability of our original data.

Thanks!

Yours sincerely,

Min Chen, M.D., Ph.D.

Figure-1A TF

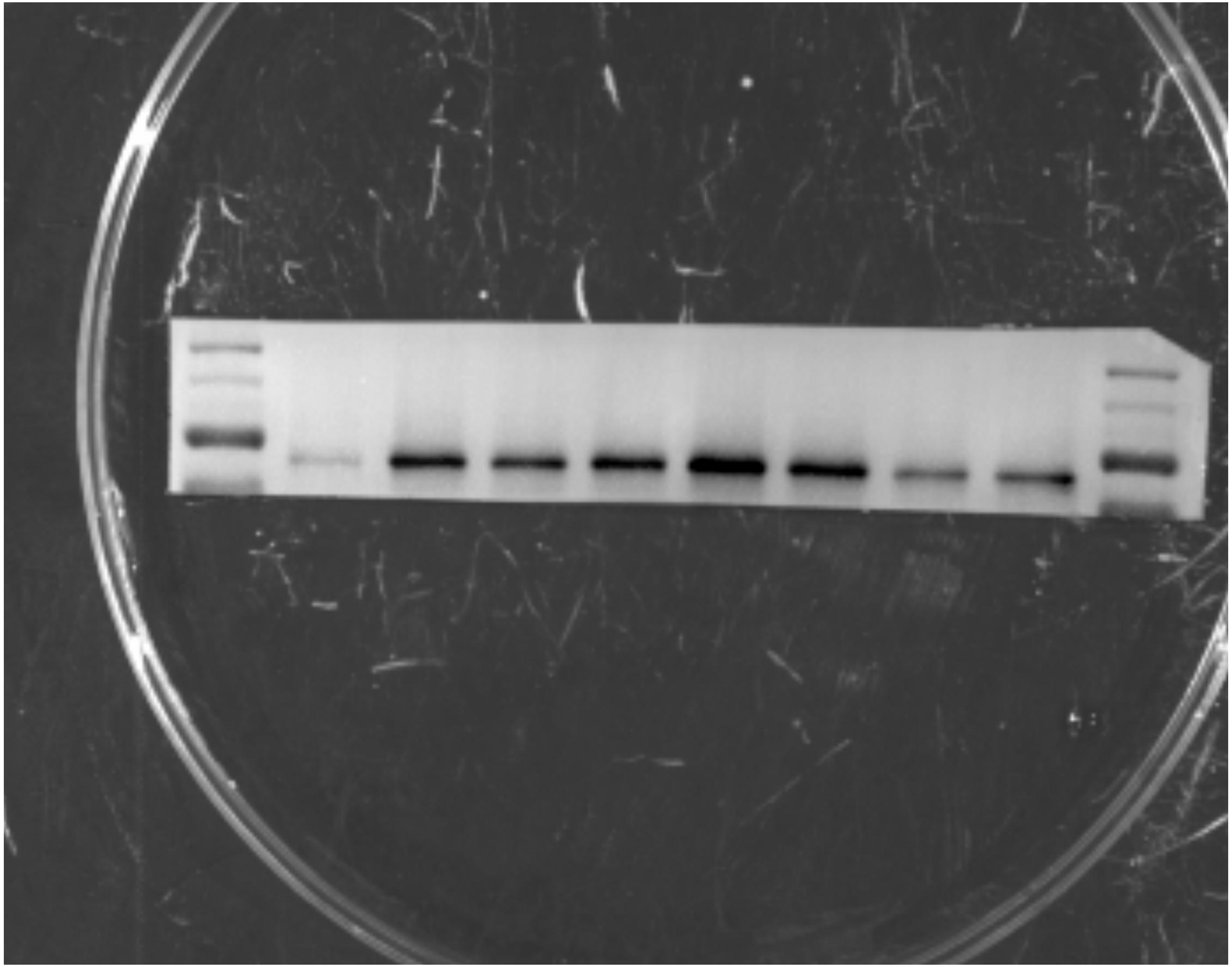

Figure-1A SCL7A11

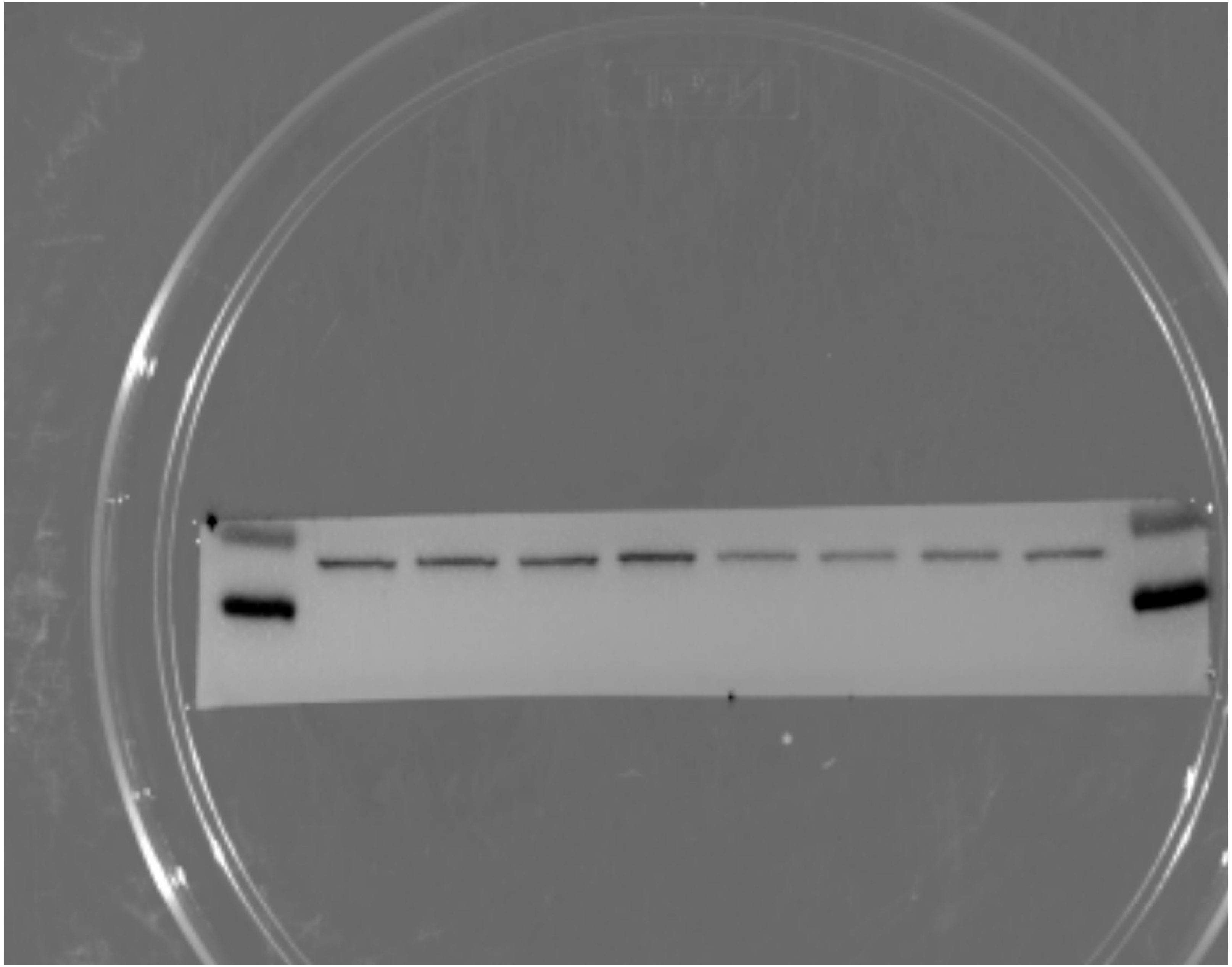

Figure-1A VDAC

VDAC

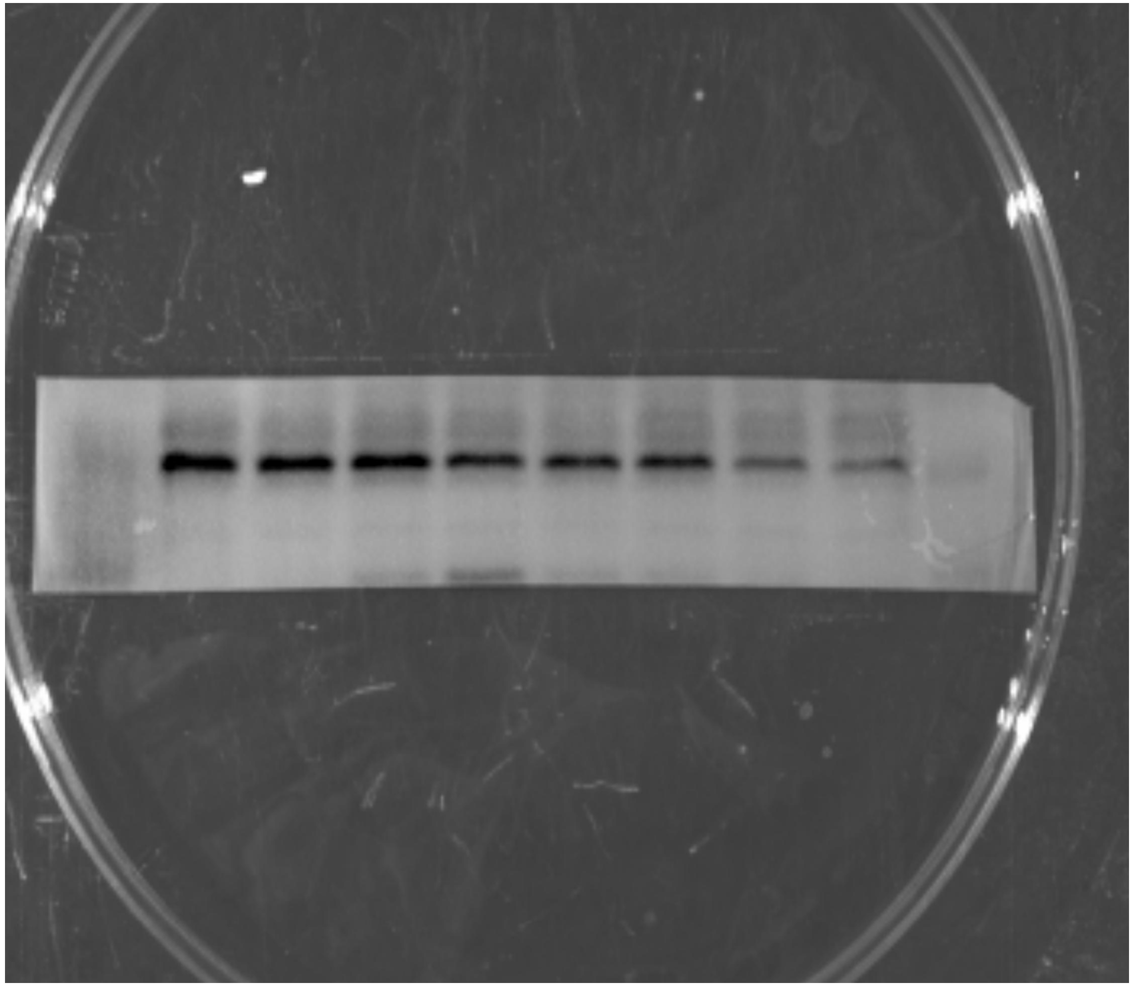

VDAC and its adjacent blot above

Merge mode

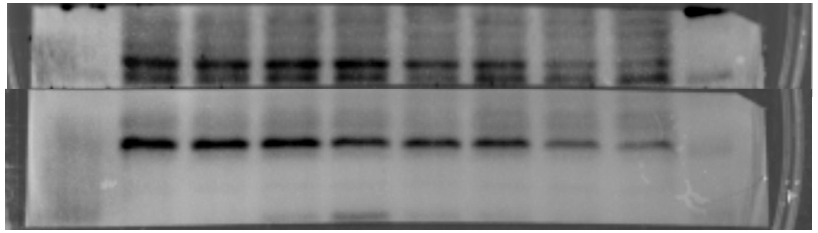

Colormeritric mode

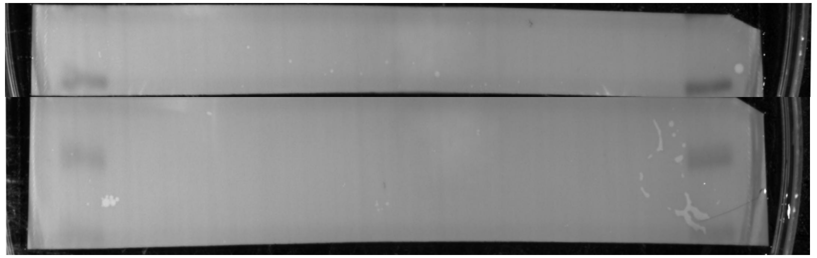

Figure-1A All orignal pictures in the spliced pictures are shown

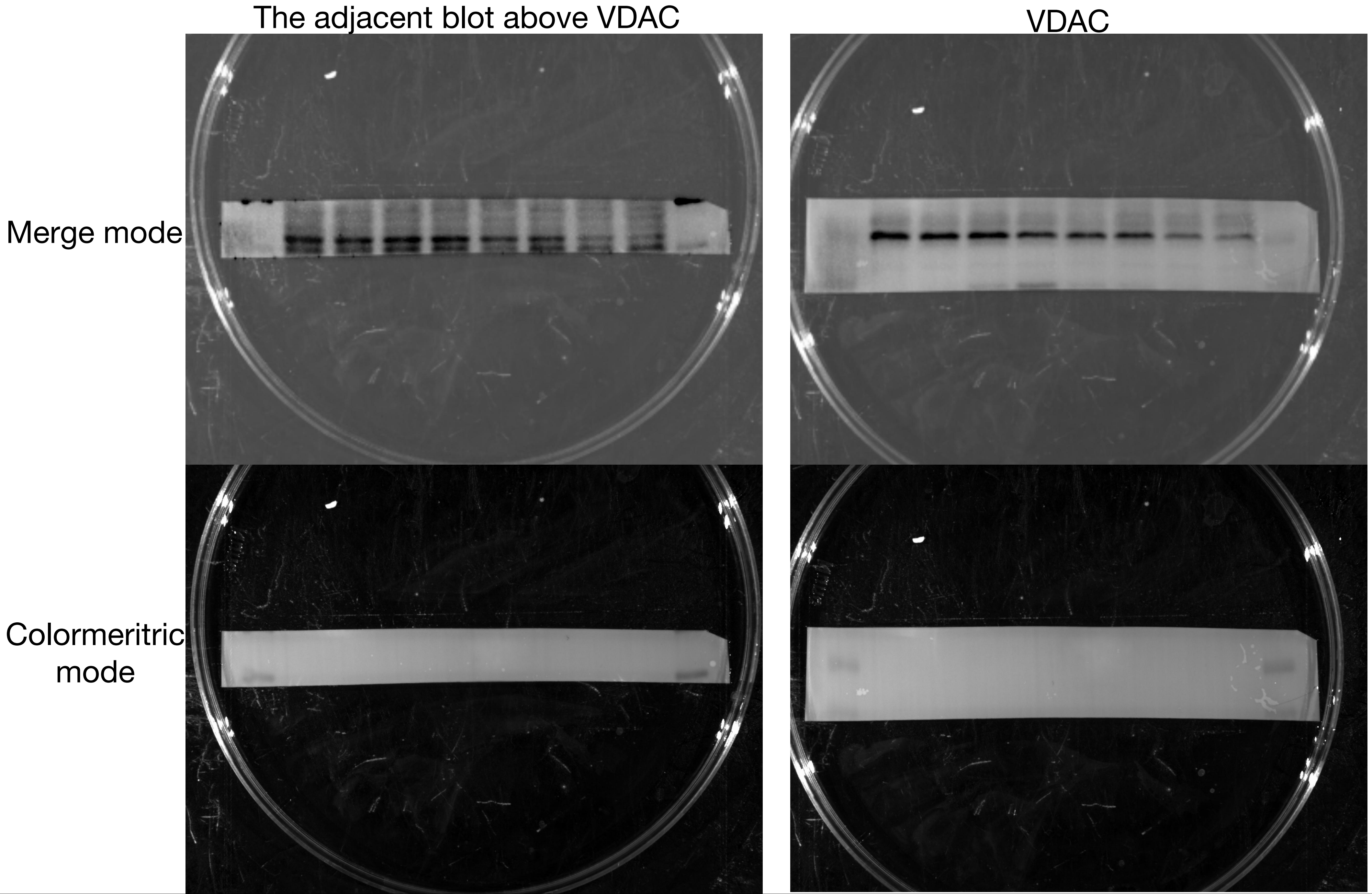

Figure-1A GPX4

Merge mode

Colormeritric mode

Adjacent Blot

Adjacent Blot

Adjacent Blot

Adjacent Blot

GAPDH

GPX4

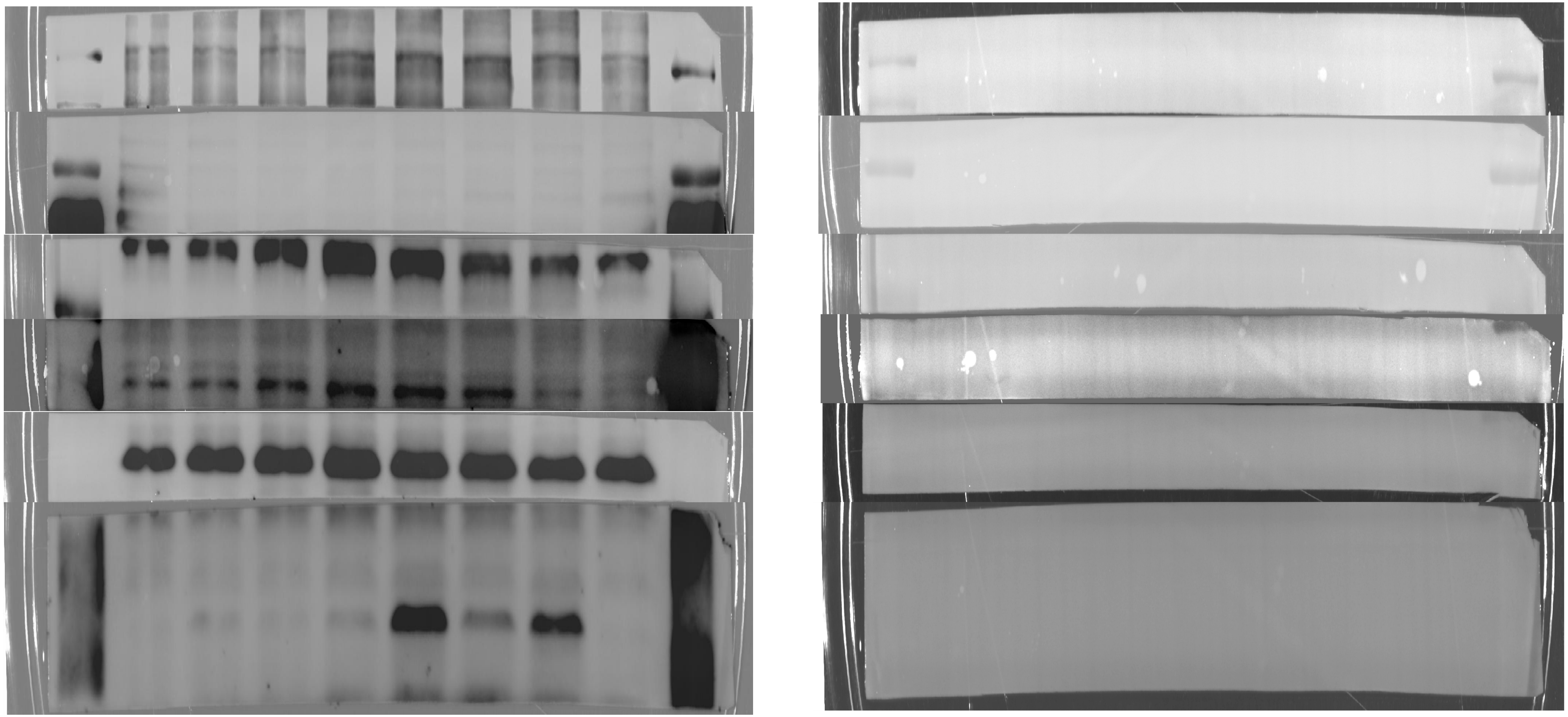

Figure-1A All orignal pictures in the spliced pictures are shown

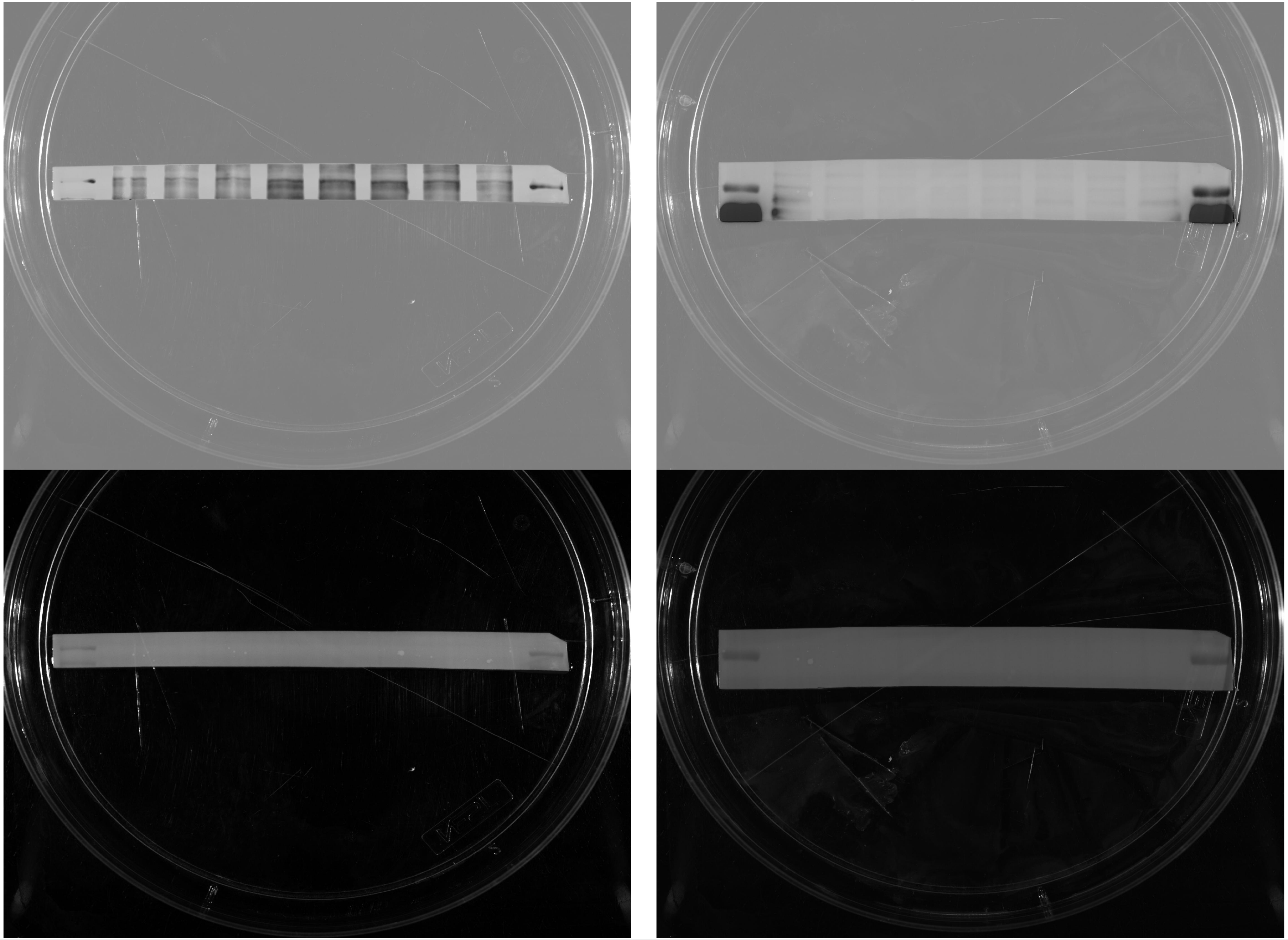

Figure-1A All orignal pictures in the spliced pictures are shown

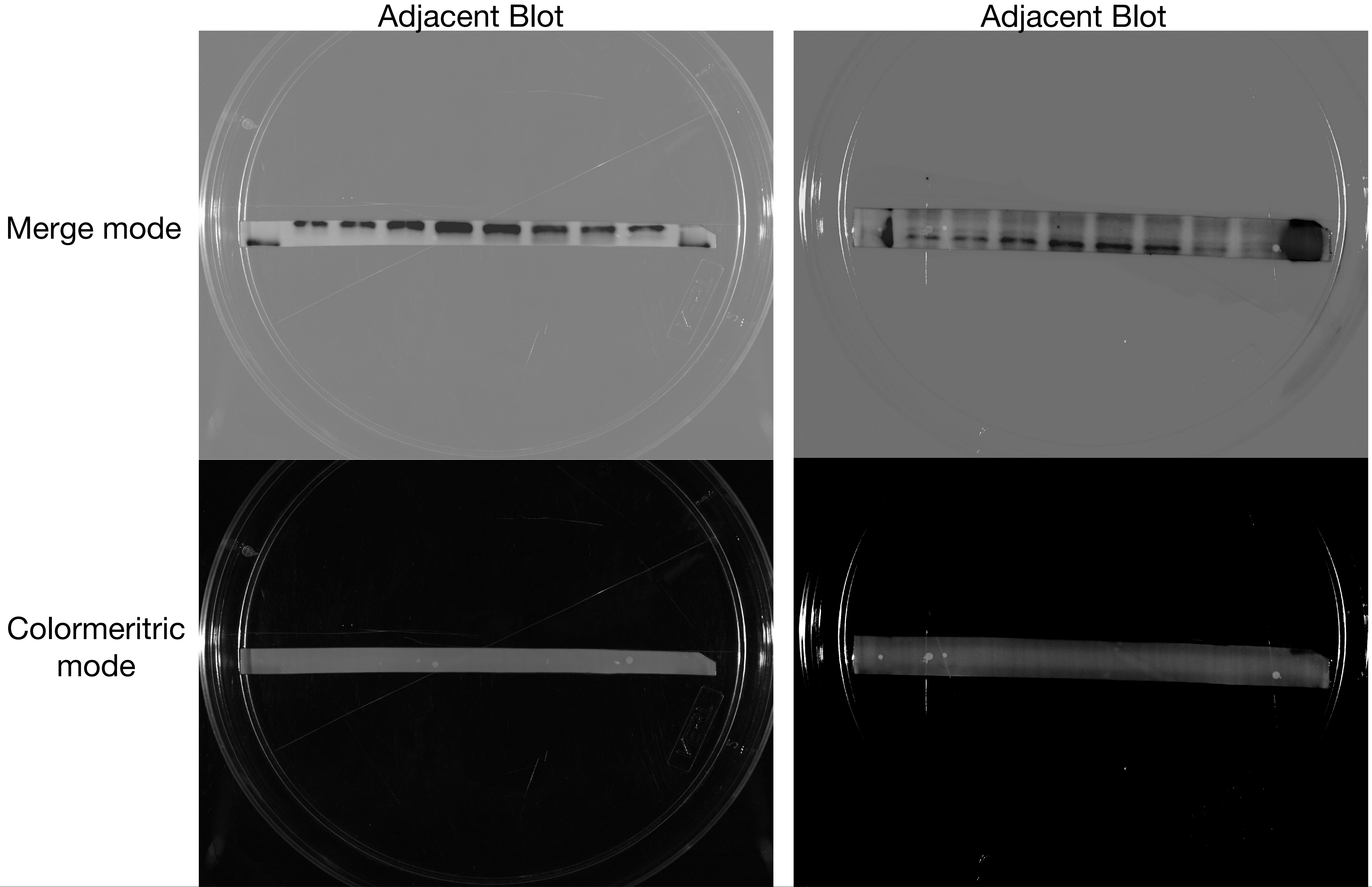

Figure-1A All orignal pictures in the spliced pictures are shown

GAPDH GPX4

Merge mode

Colormeritric mode

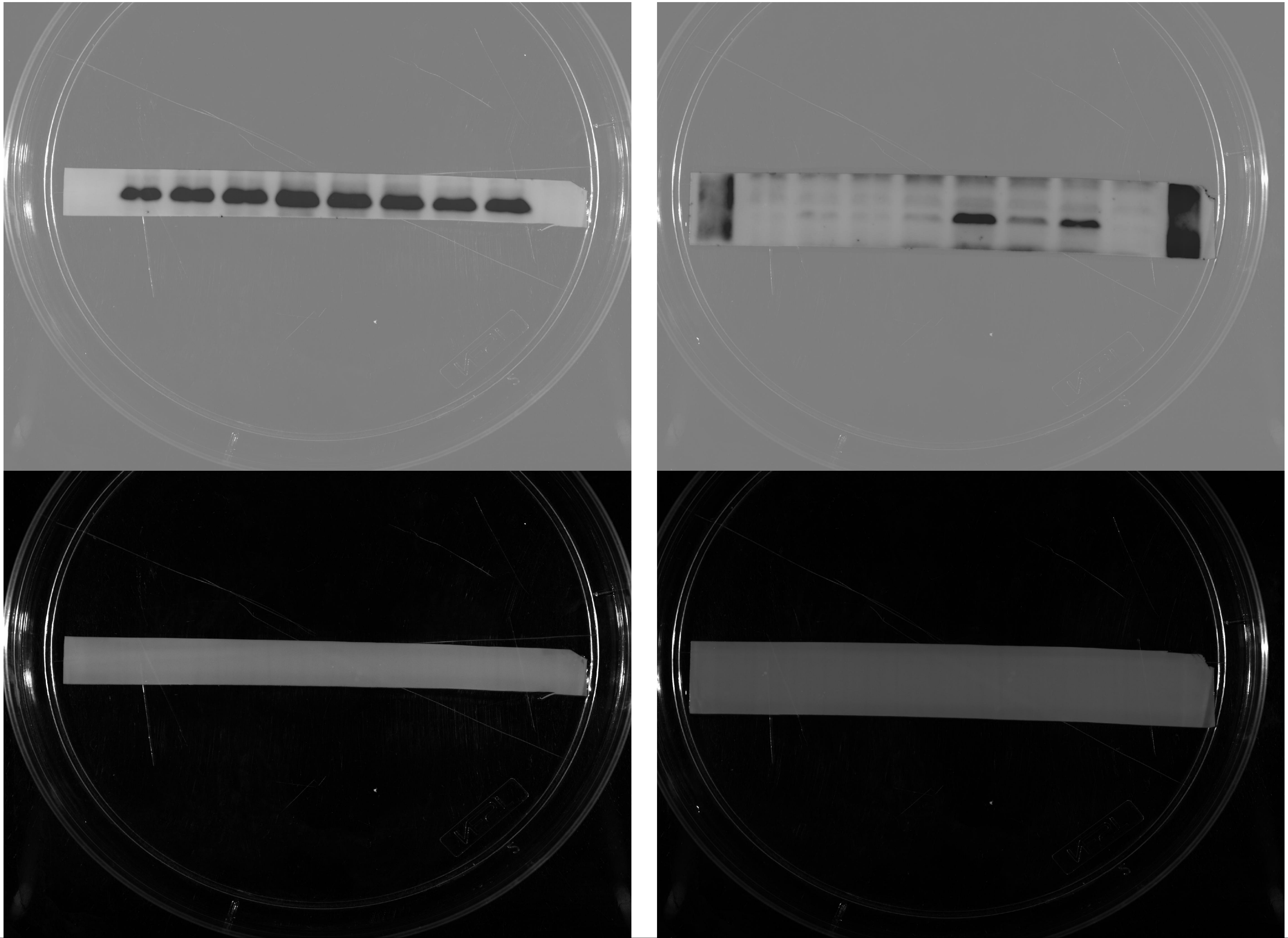

Figure-1A FSP1

FSP1

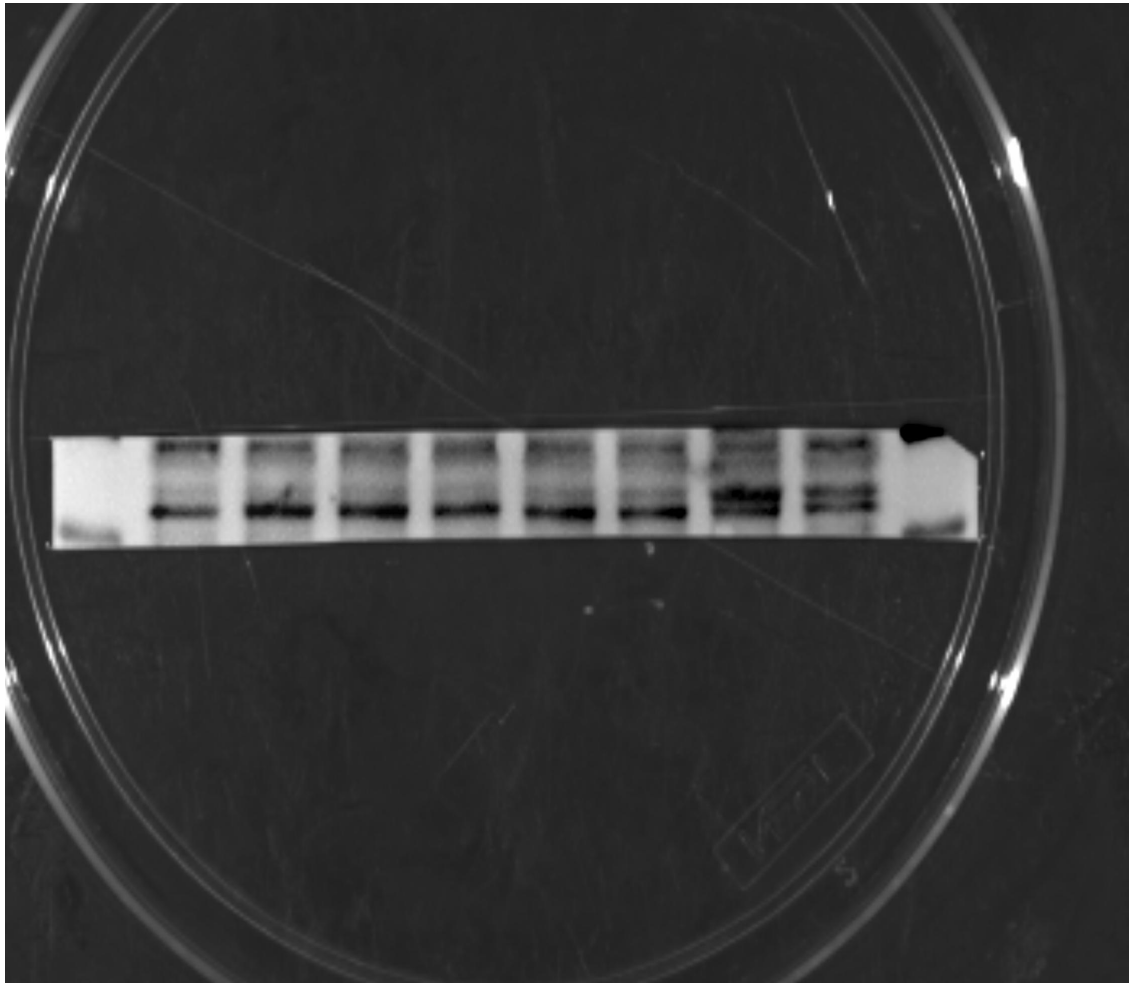

FSP1 and its adjacent blot above

Merge mode

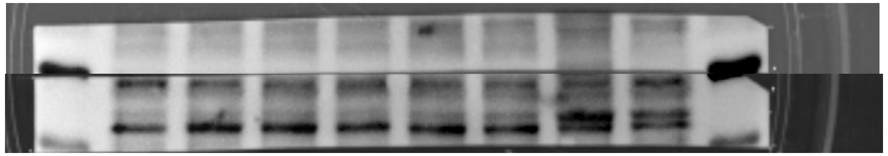

Colormeritric mode

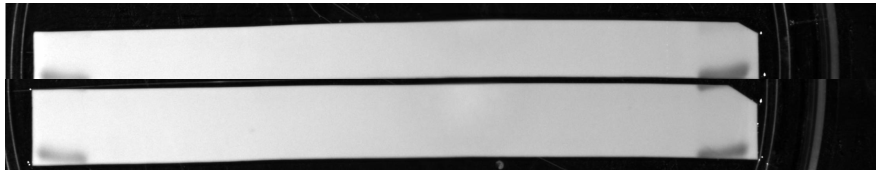

Figure-1A All orignal pictures in the spliced pictures are shown

The adjacent blot above FSP1

FSP1

Merge mode

Colormeritric mode

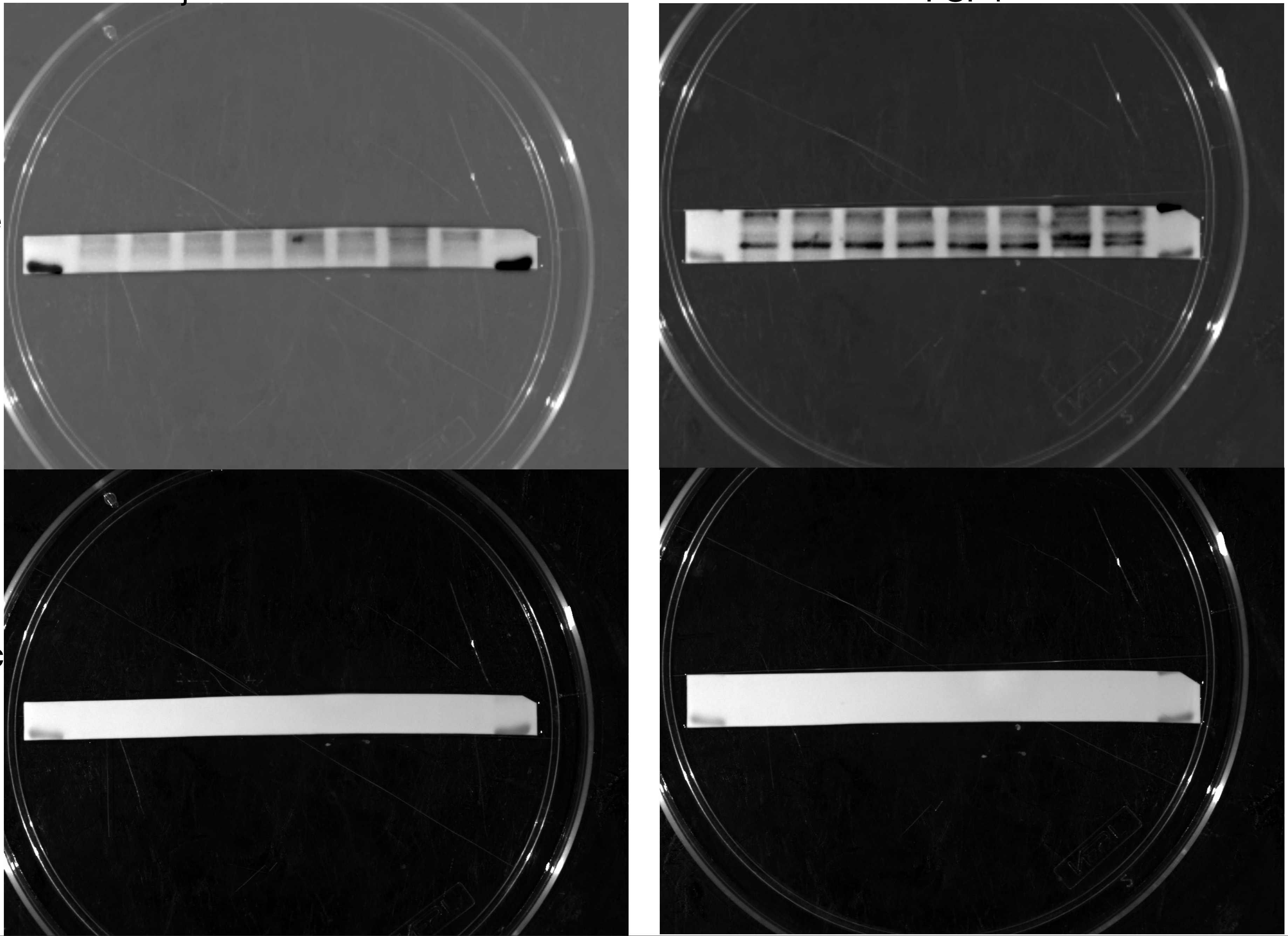

Figure-1A ACSL4

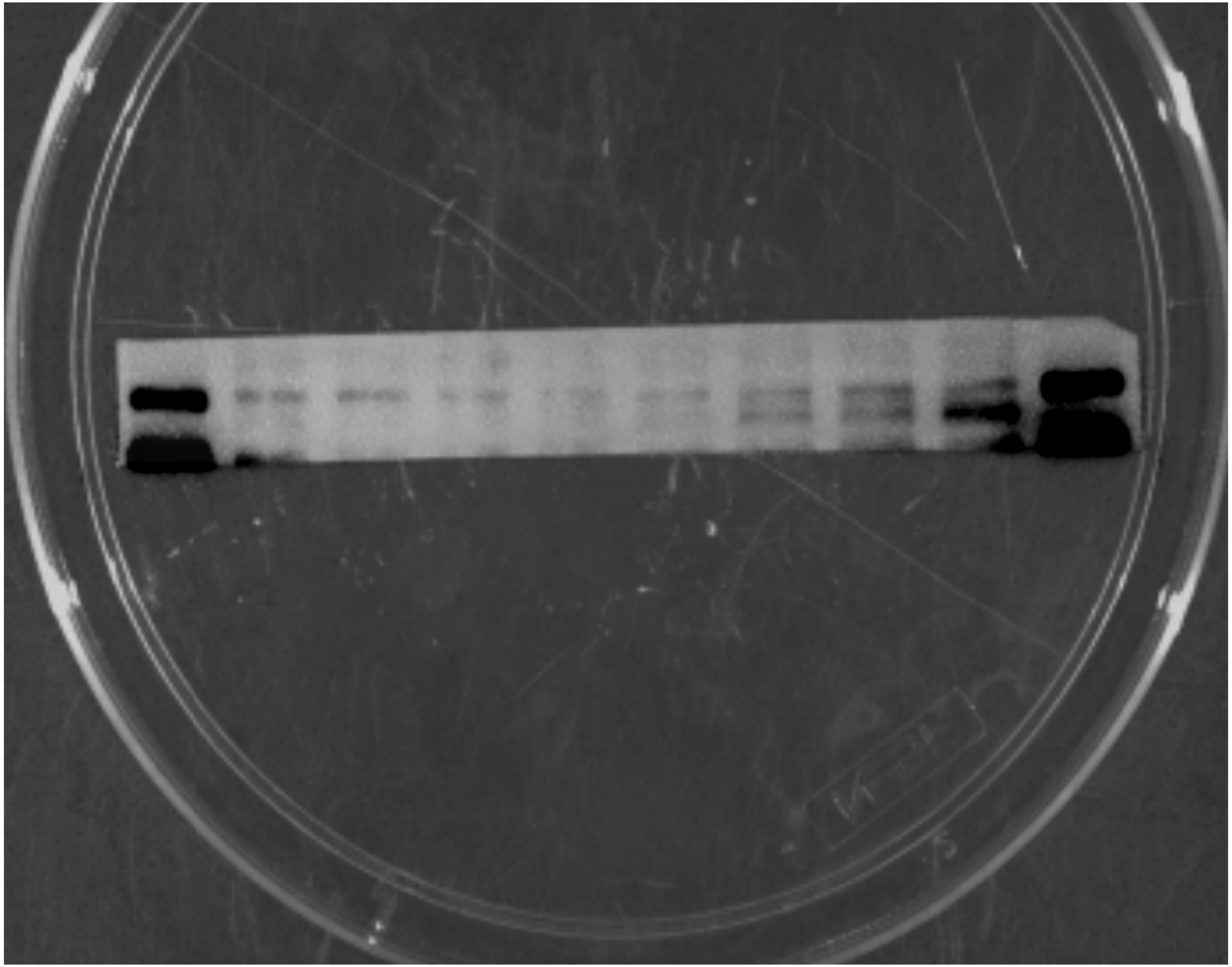

Figure-1A GAPDH

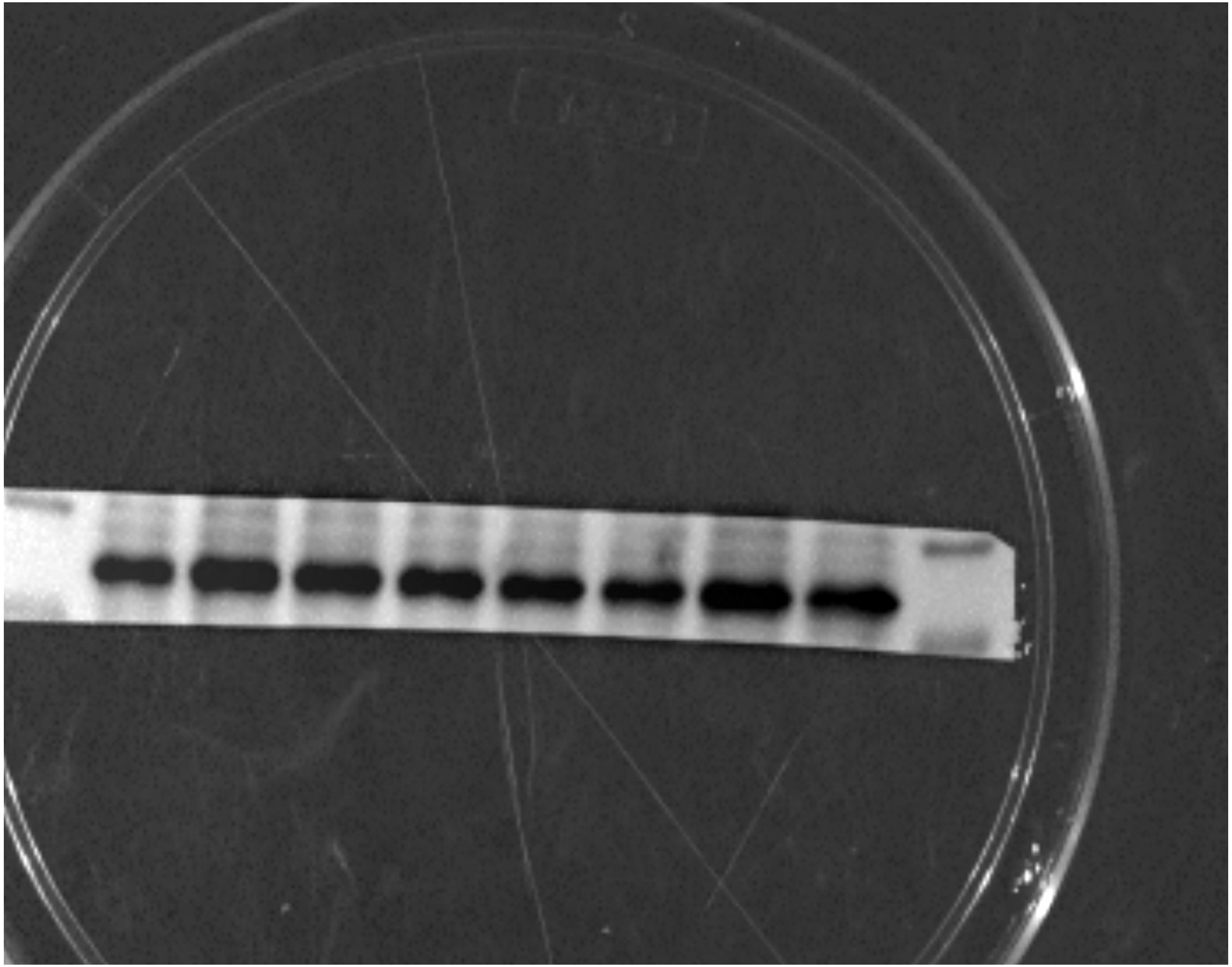

Figure-1B RIP1, RIP3 and GAPDH are all from one membrane

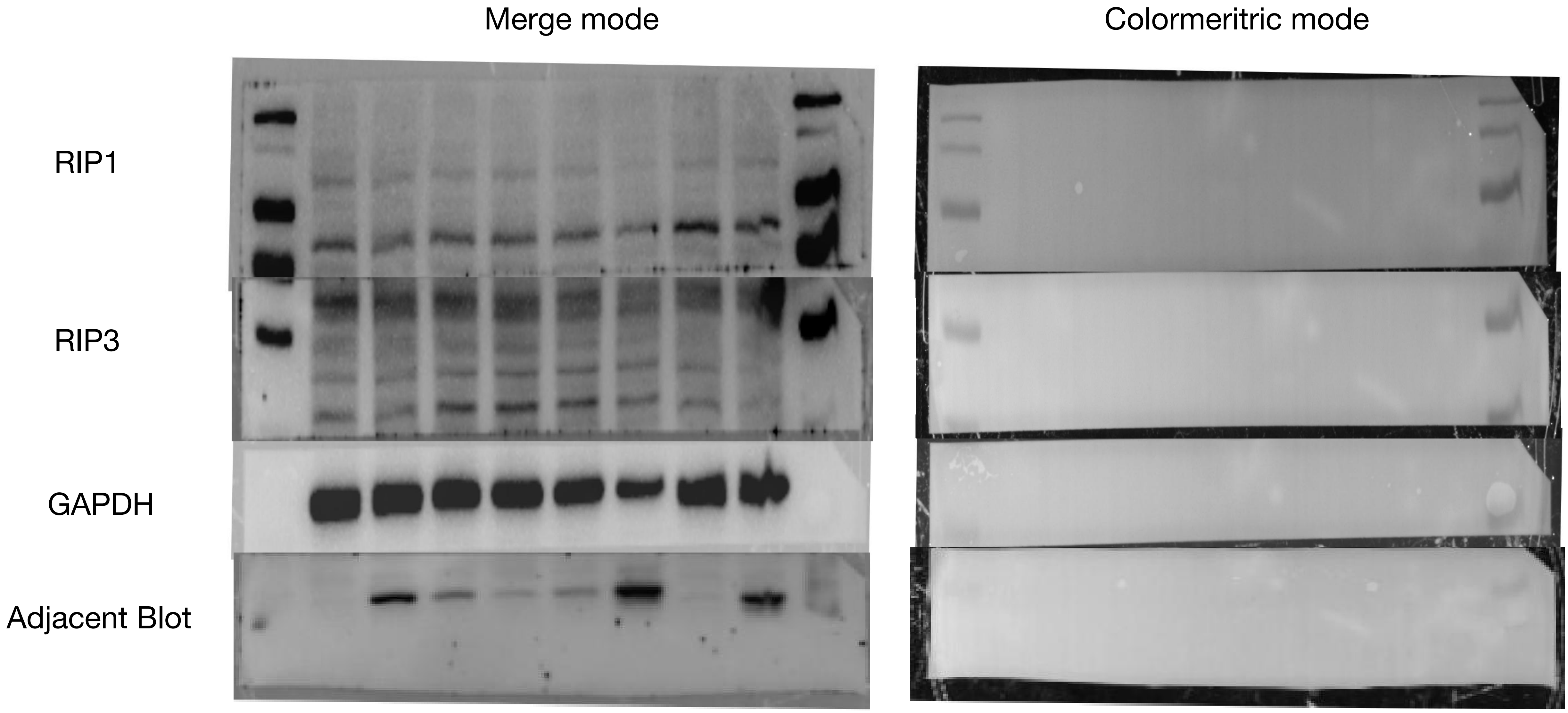

Figure-1B All orignal pictures in the spliced pictures are shown

RIP1 RIP3

Merge mode

Colormeritric mode

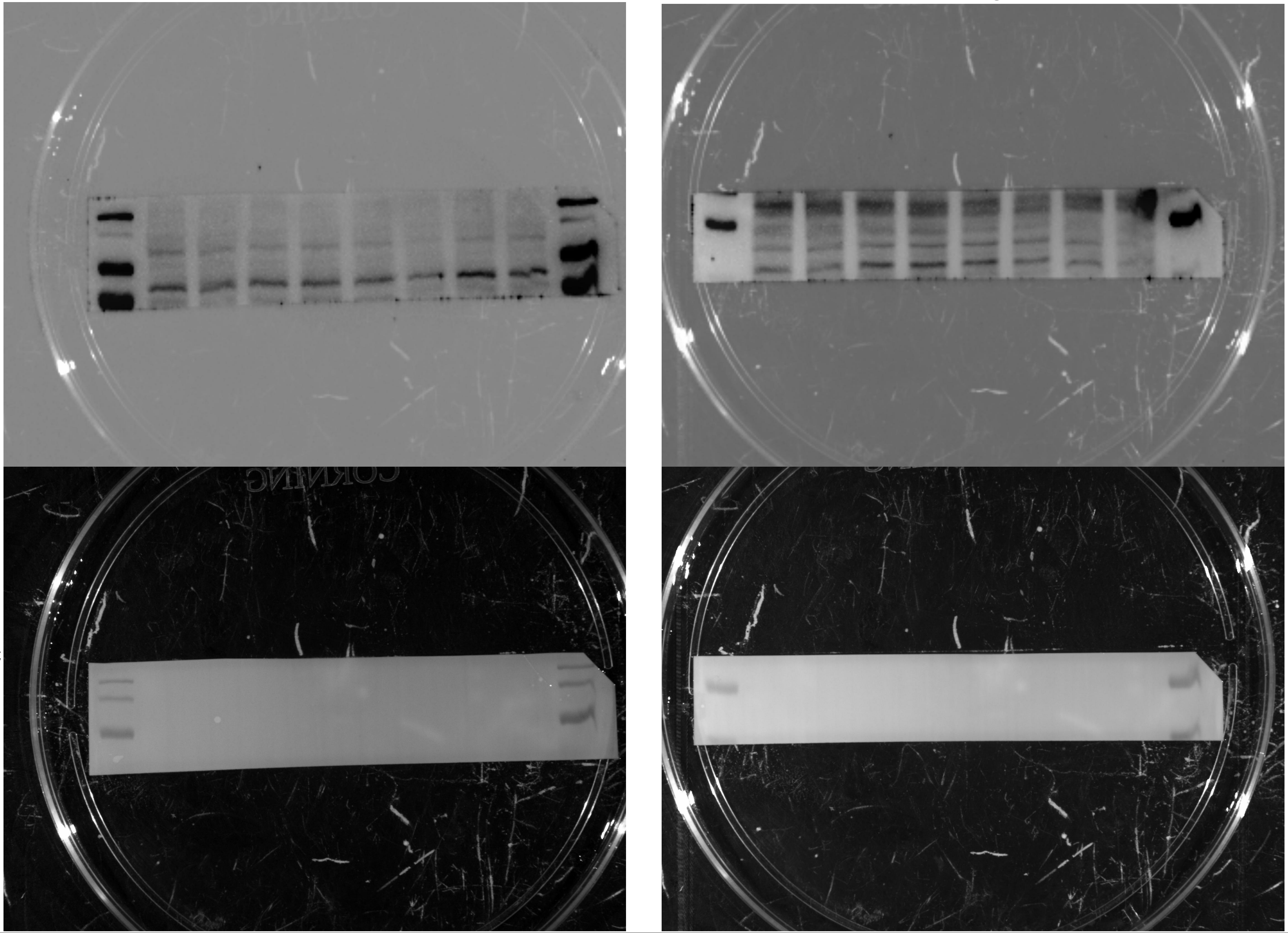

Figure-1B All orignal pictures in the spliced pictures are shown

GAPDH | The adjacent blots below GAPDH

Merge mode

Colormeritric mode

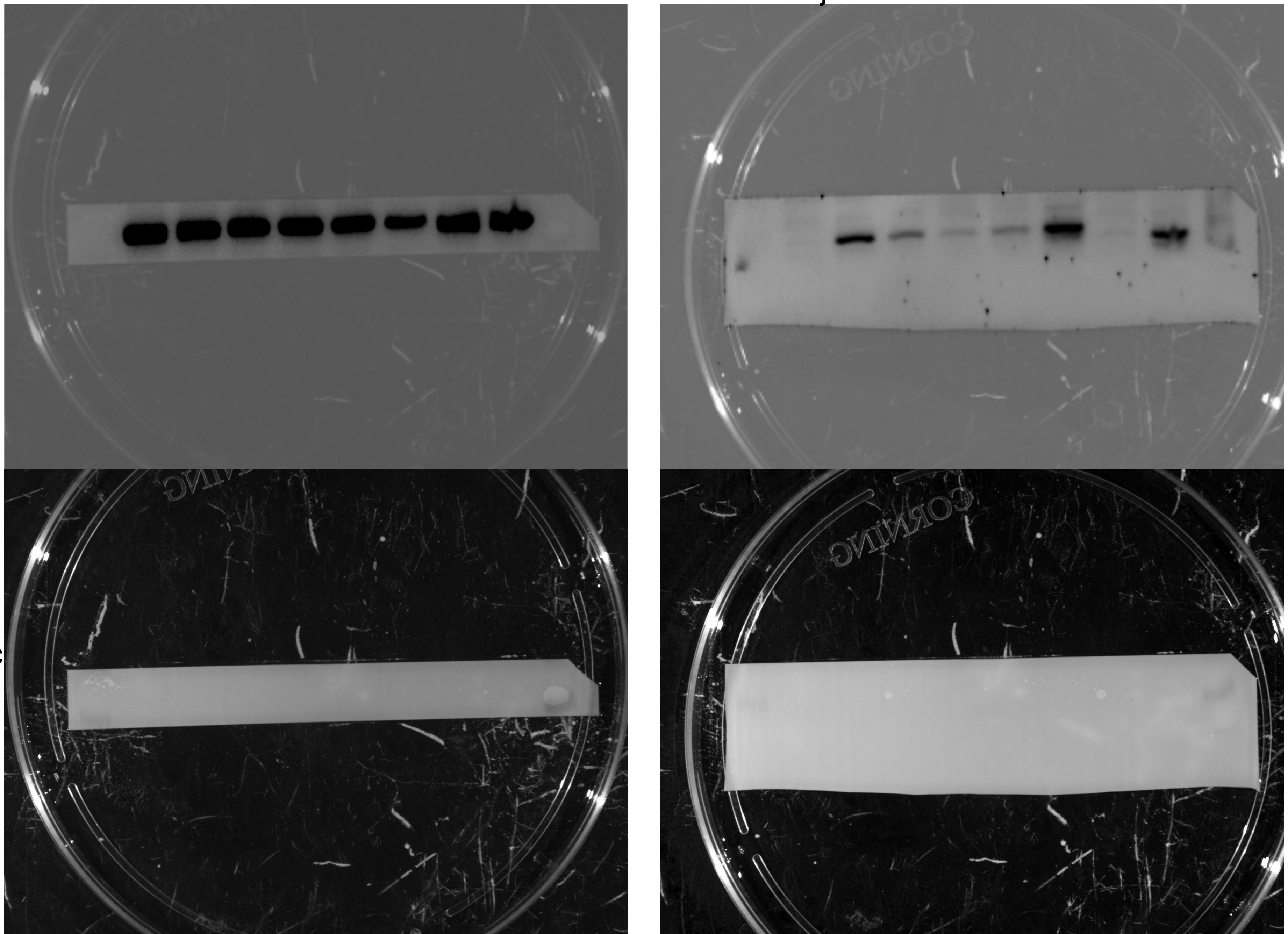

Figure-1B Pro-Caspase 8

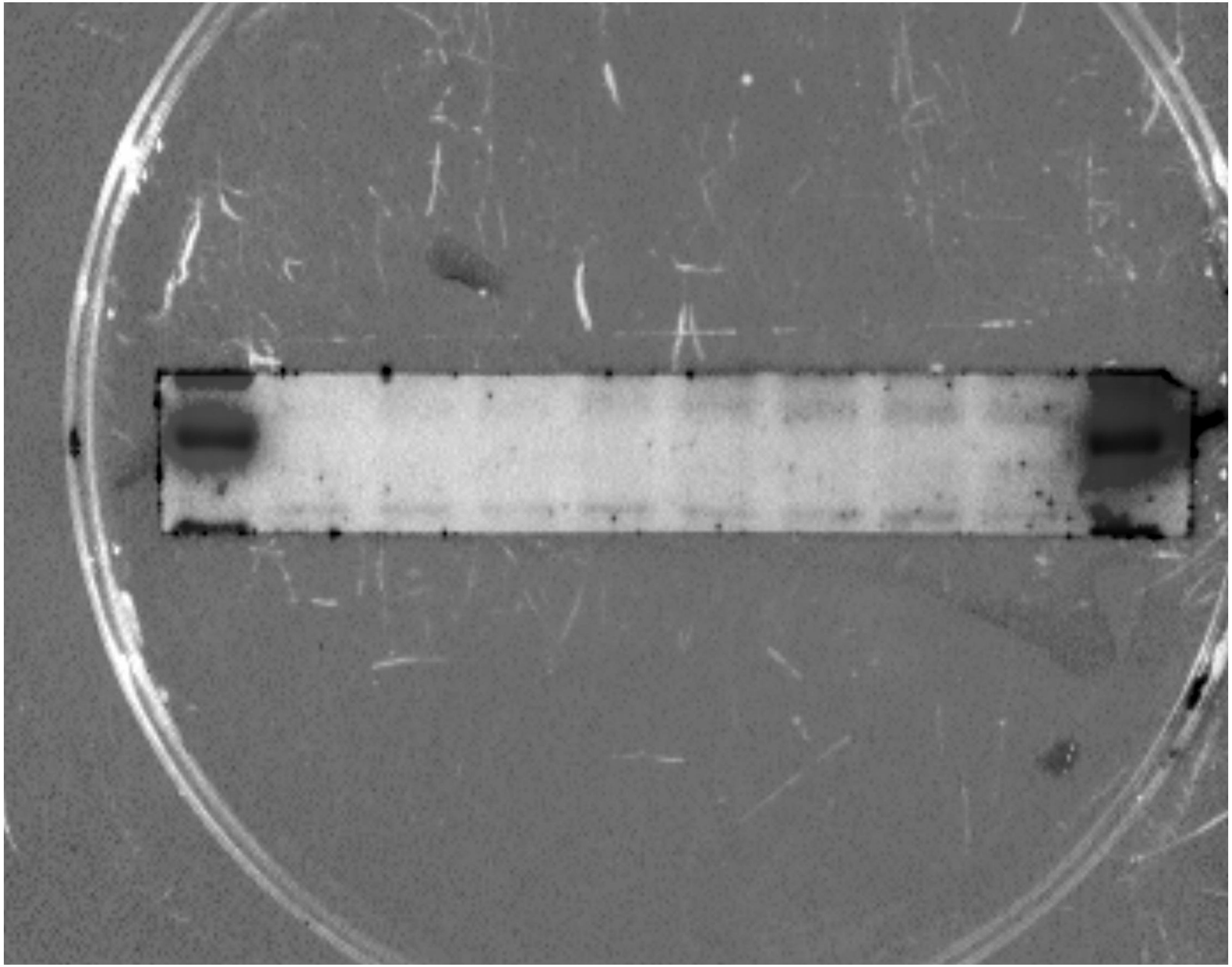

Figure-1C Pro-Caspase 9, Cleaved Caspase3 and GAPDH are all from one membrane

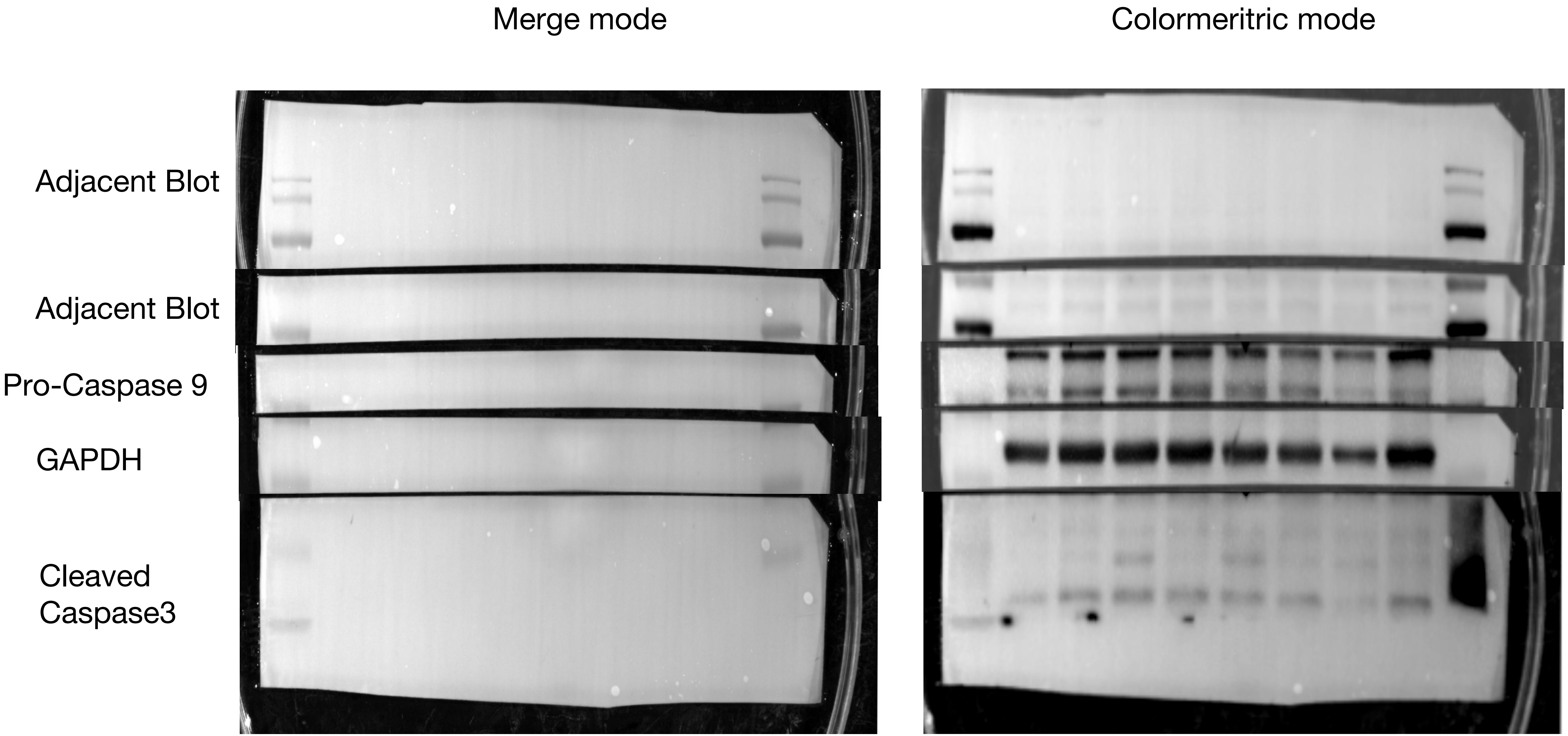

Figure-1C All orignal pictures in the spliced pictures are shown

The adjacent bolt

The adjacent bolt above Caspase 9

Merge mode

Colormeritric mode

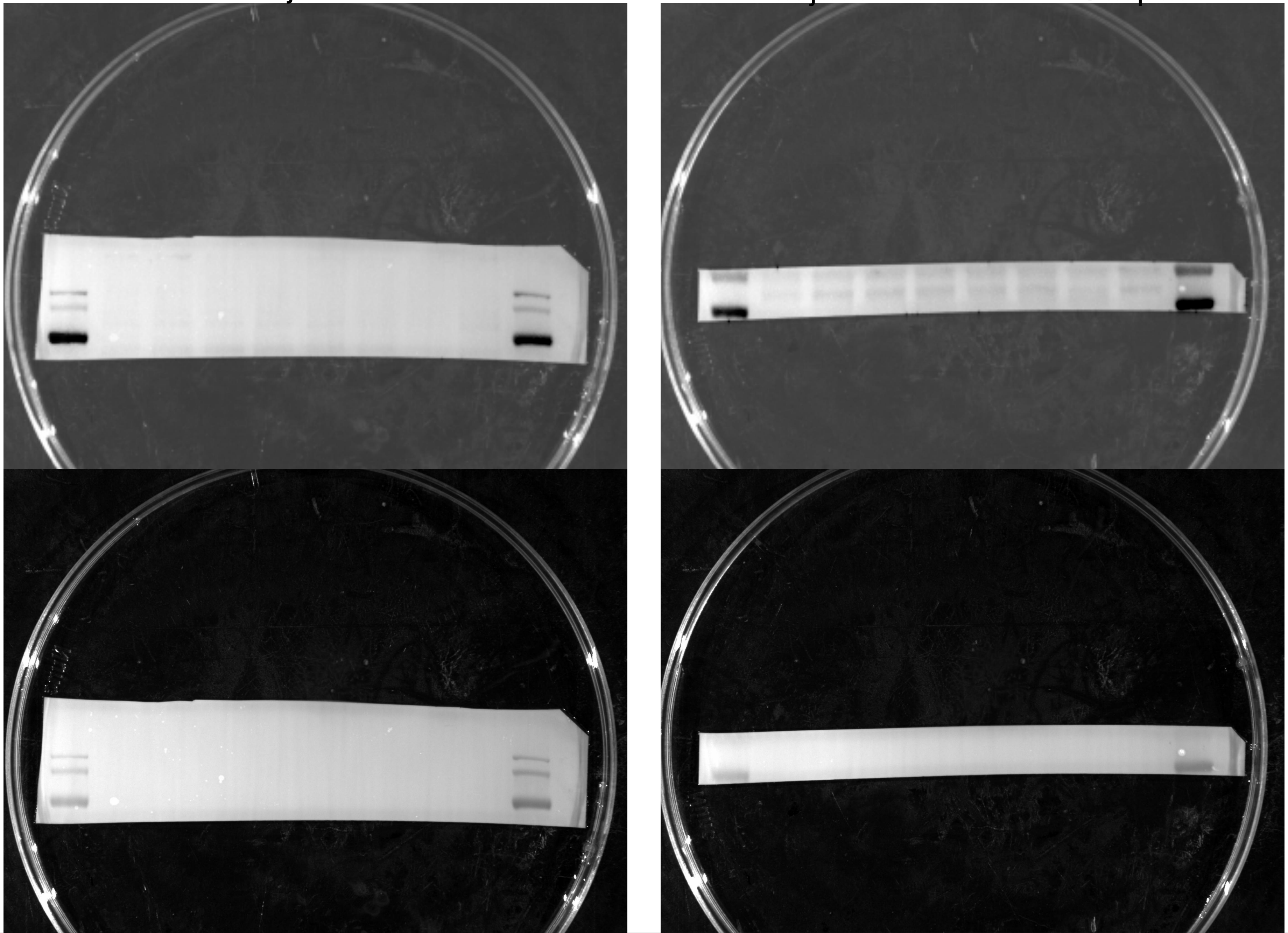

Figure-1C All orignal pictures in the spliced pictures are shown

Caspase 9

GAPDH

Merge mode

Colormeritric mode

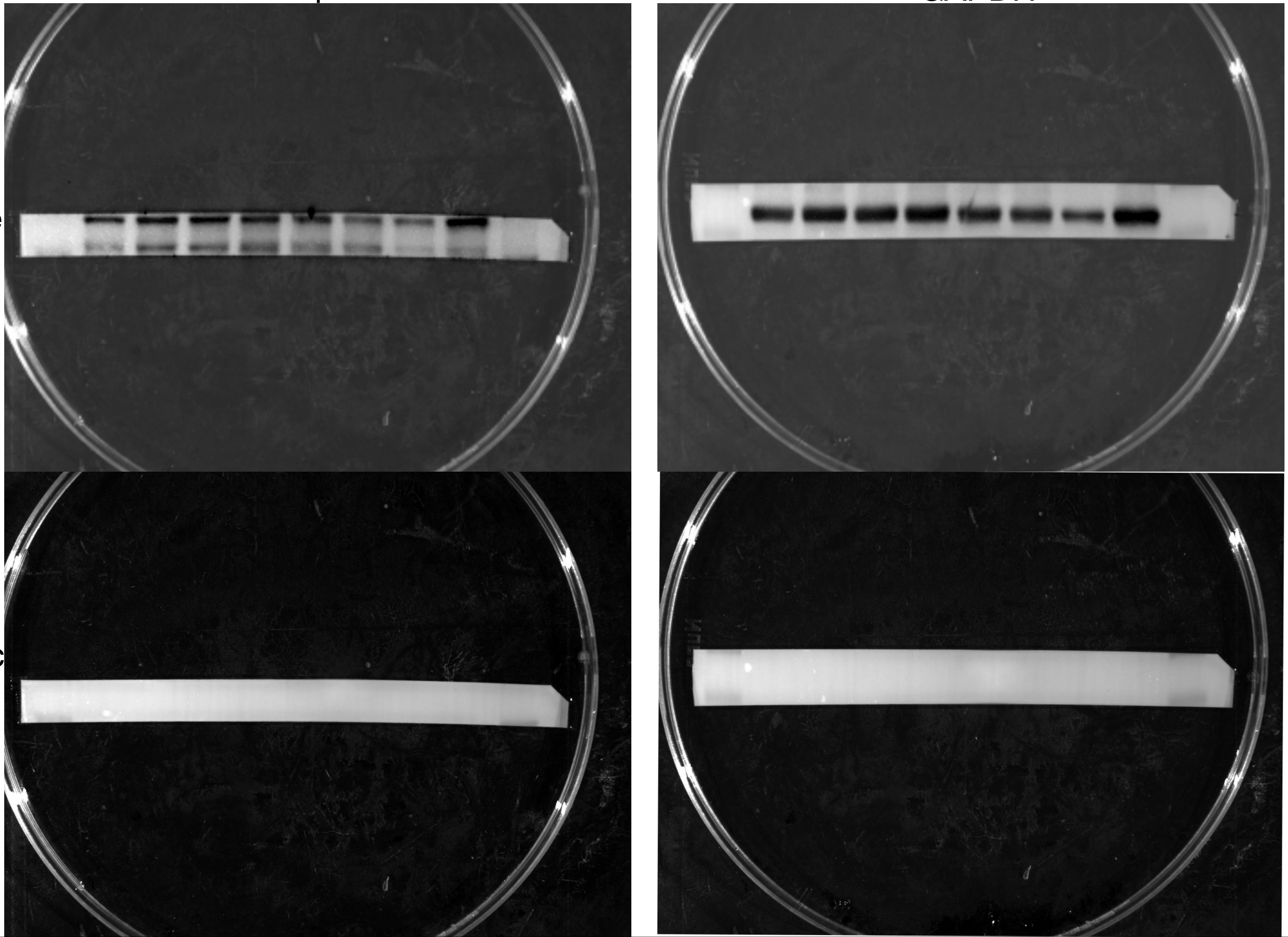

Figure-1C All orignal pictures in the spliced pictures are shown

Cleaved Caspase 3

Merge mode

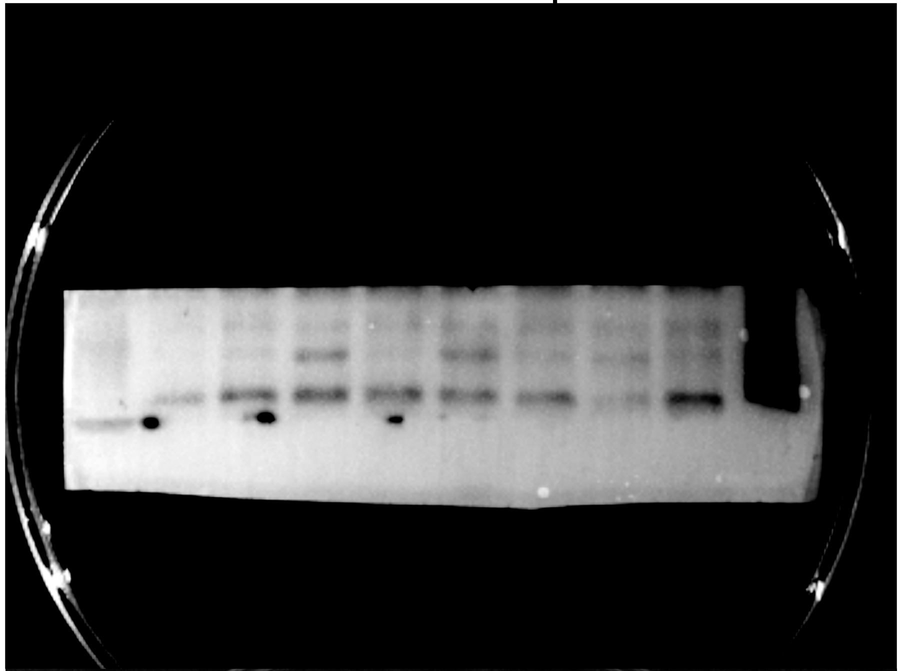

Colormeritric mode

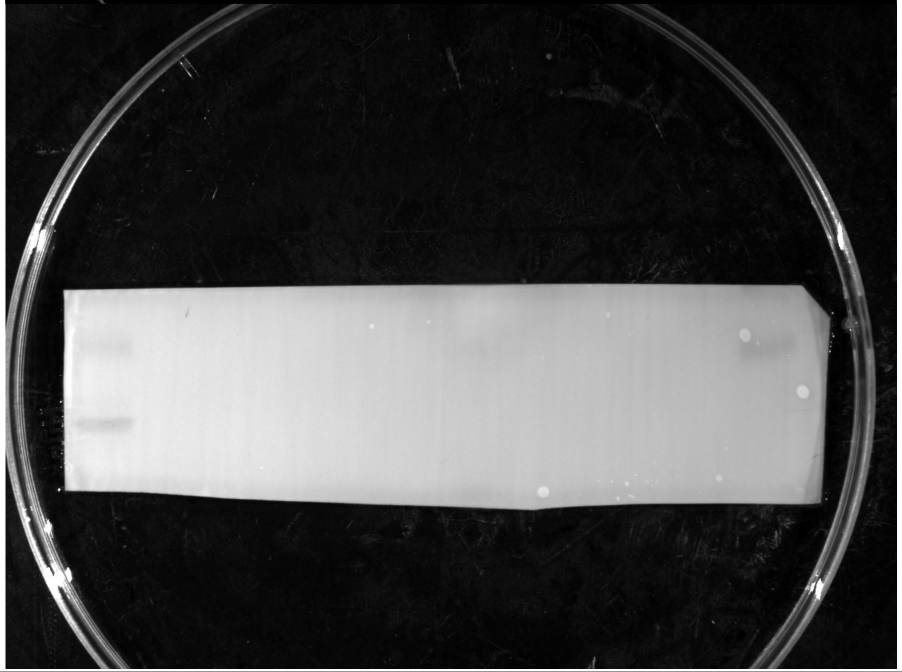

Figure-1C Bcl-2 26kDa

Bcl-2

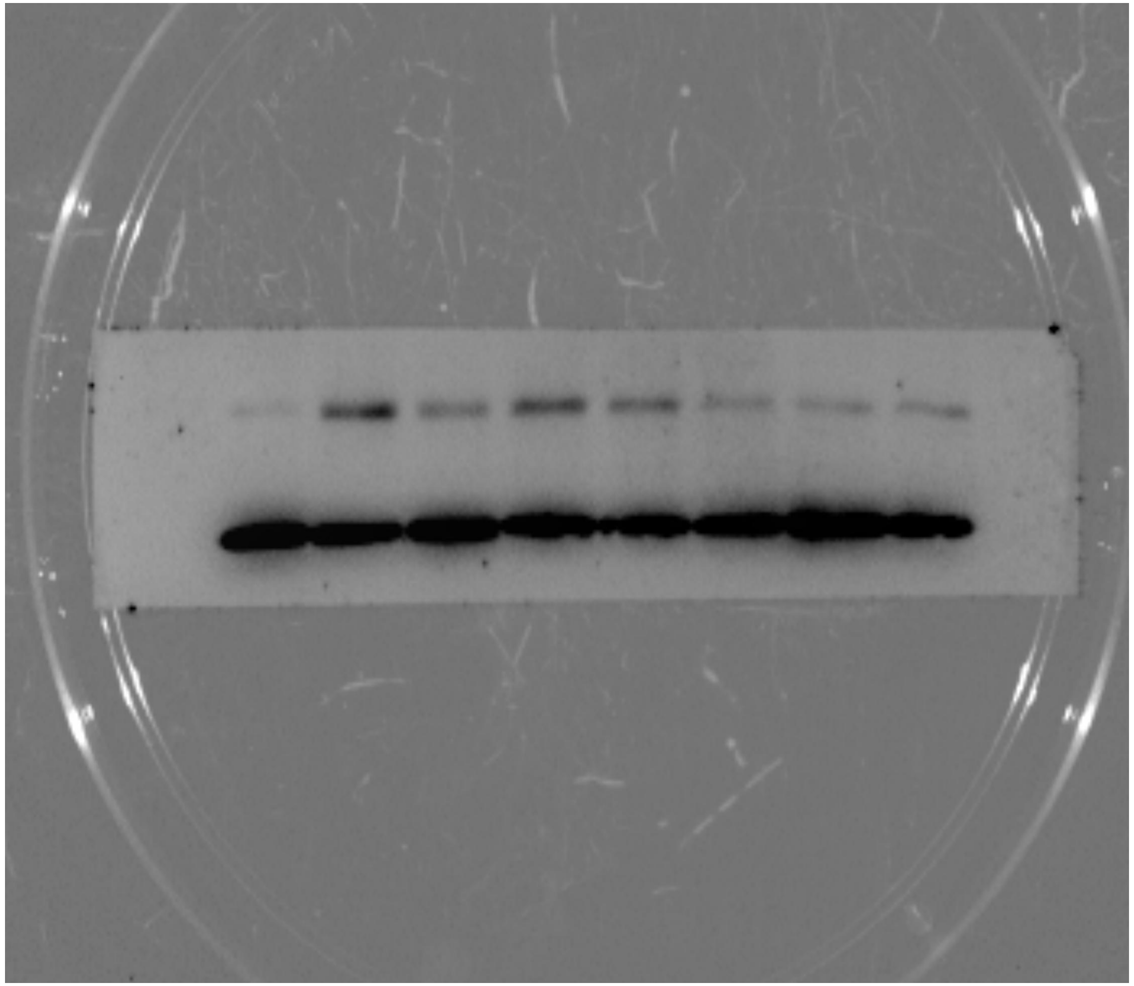

Bcl-2 and its adjacent blot above

Merge mode

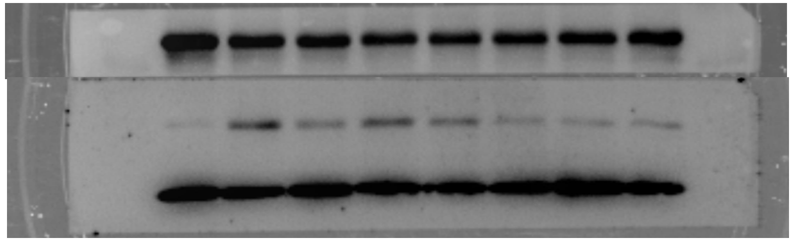

Colormeritric mode

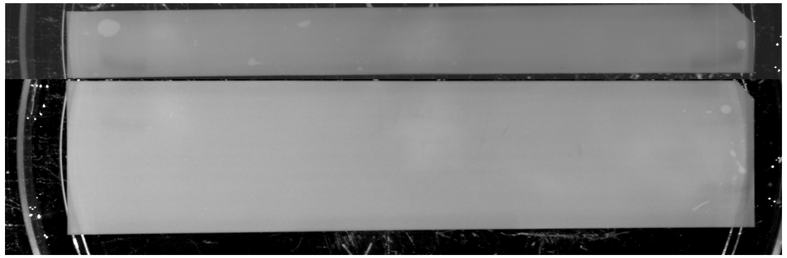

Figure-1C All orignal pictures in the spliced pictures are shown

The adjacent bolt above Bcl-2 | Bcl-2

Merge mode

Colormeritric mode

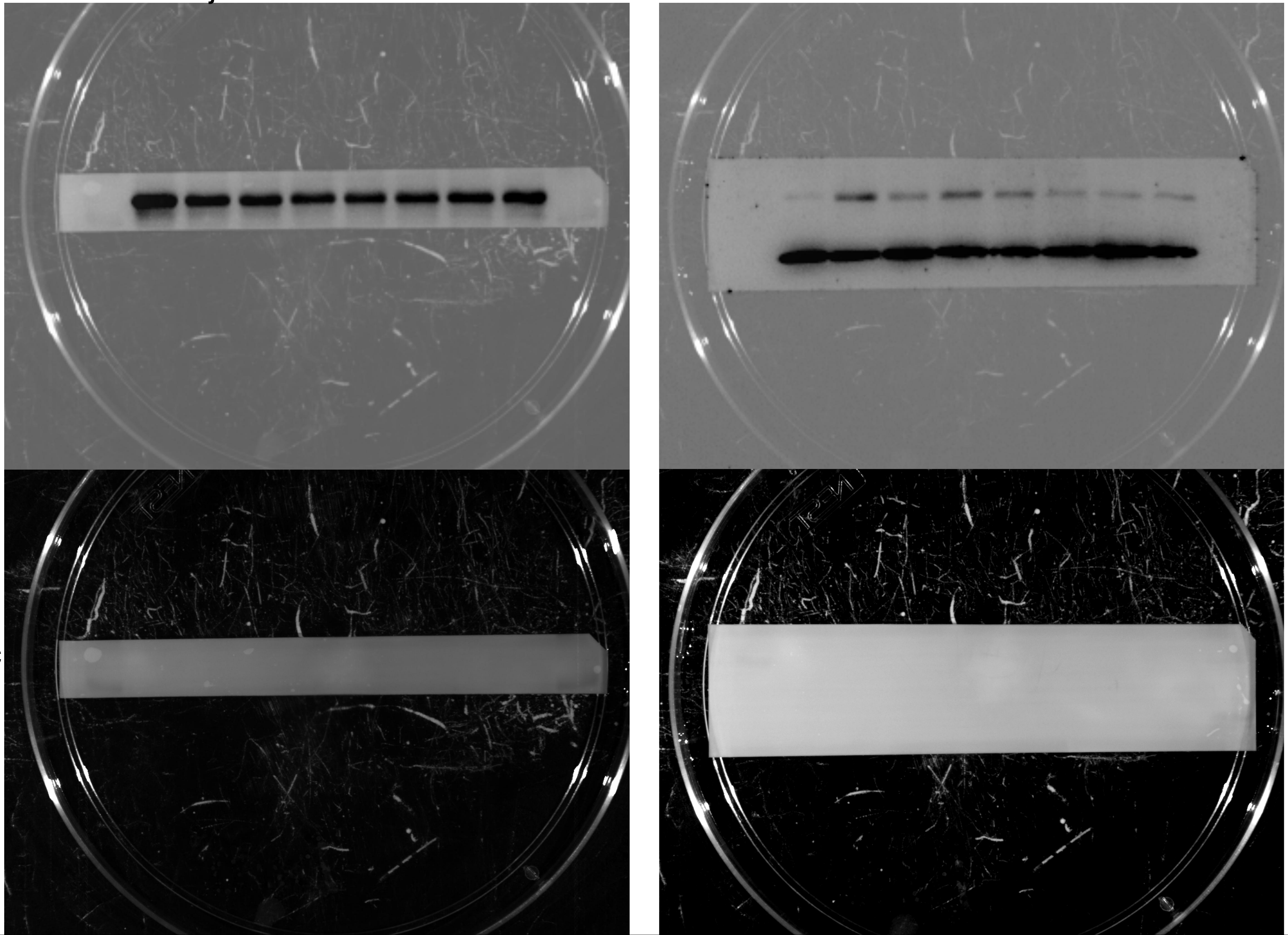

Figure-4 3D TFR1

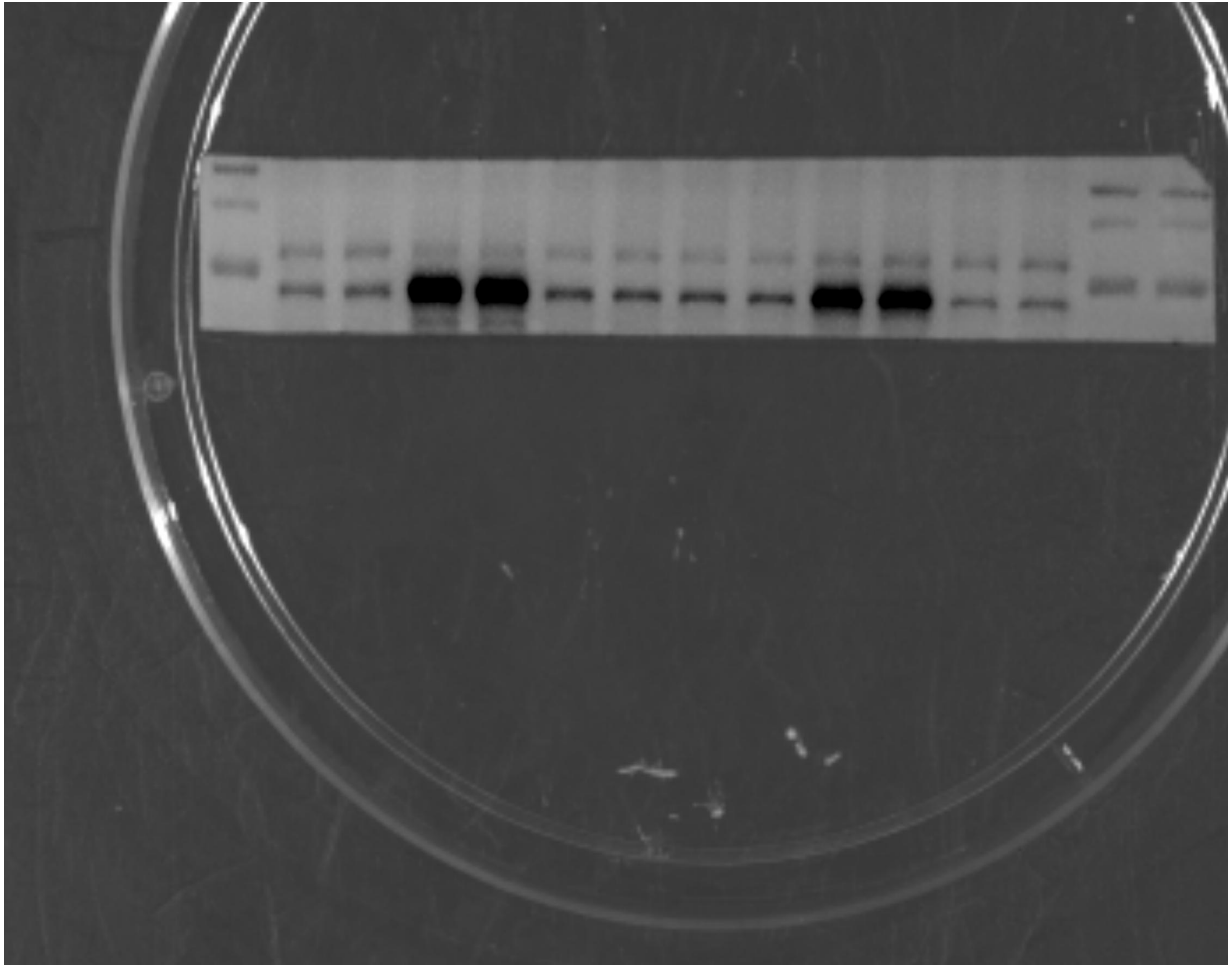

Figure-4 3D TF, Cox2, NeuN, GAPDH and Cleaved Caspase3 are all from one membrane

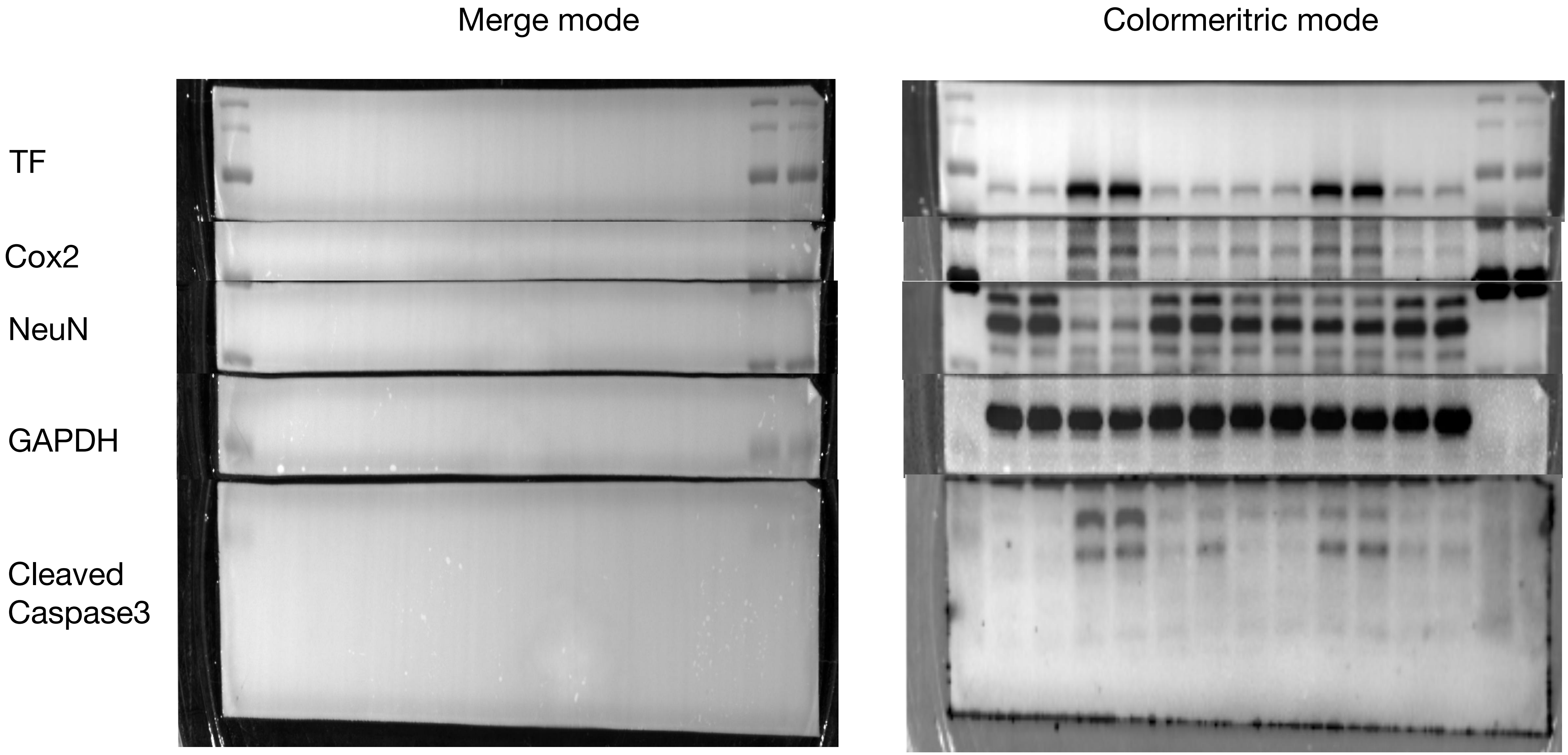

Figure-4 3D All orignal pictures in the spliced pictures are shown

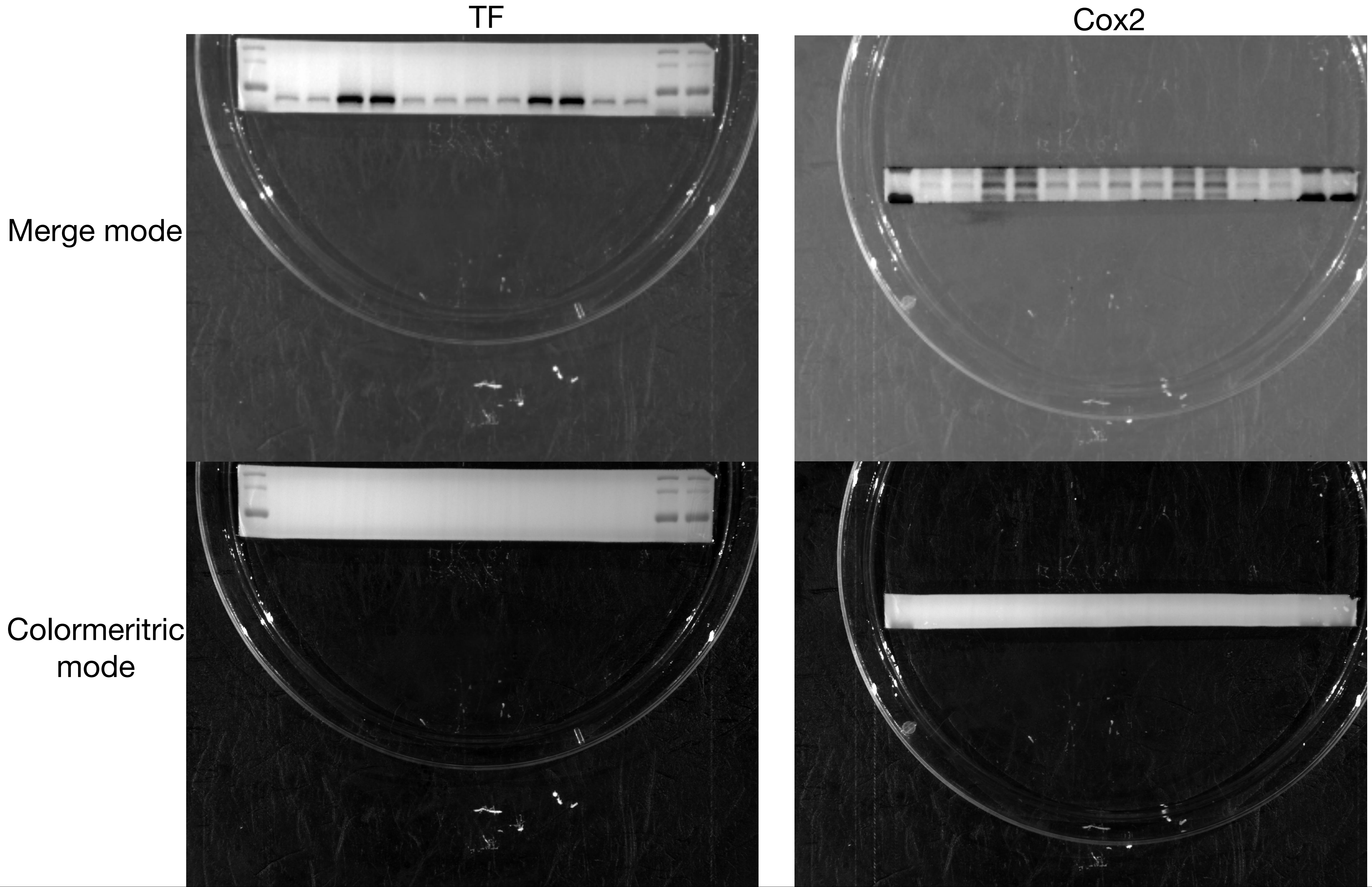

Figure-4 3D All orignal pictures in the spliced pictures are shown

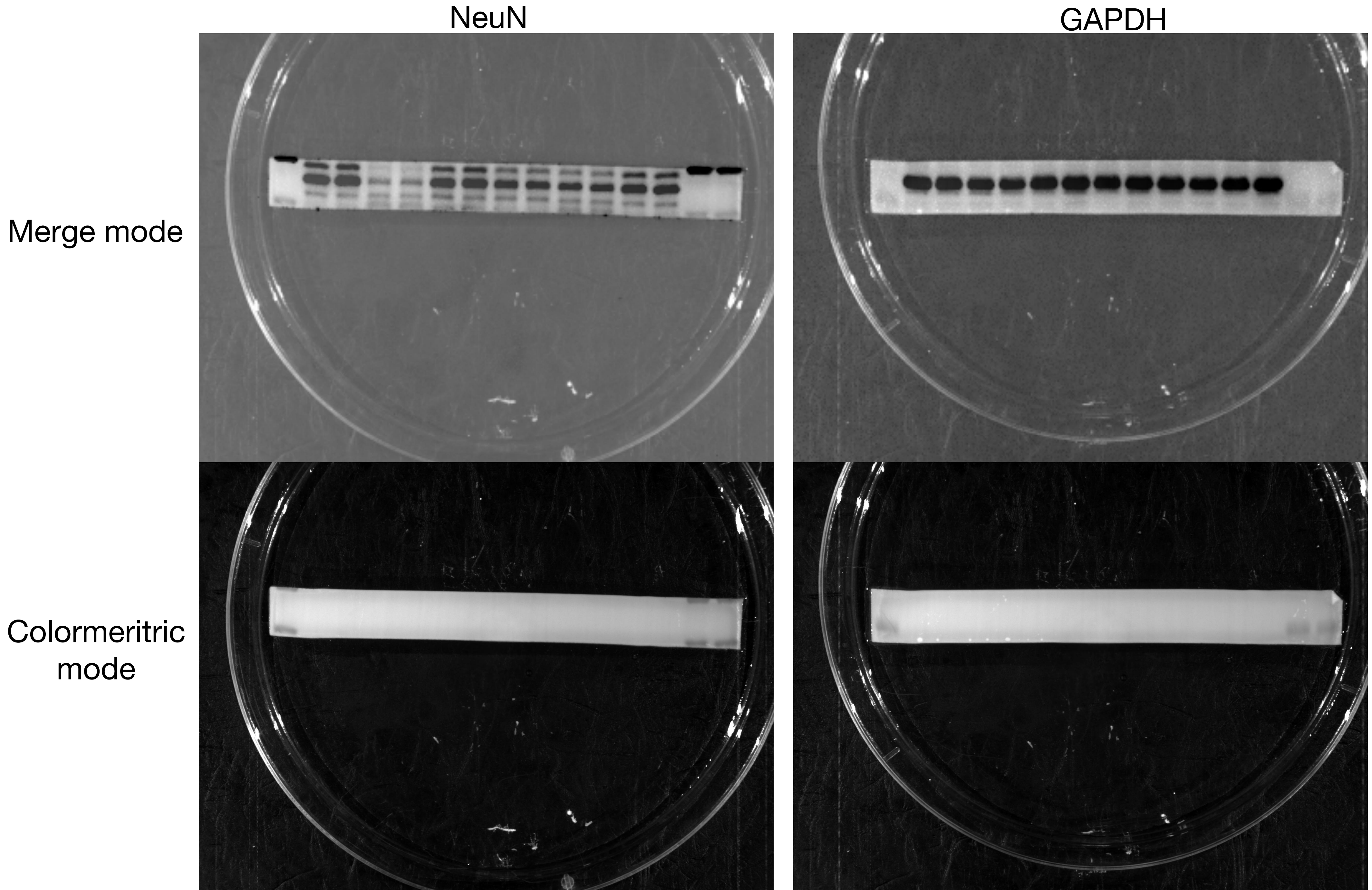

Figure-4 3D All orignal pictures in the spliced pictures are shown

Cleaved Caspase3

Merge mode

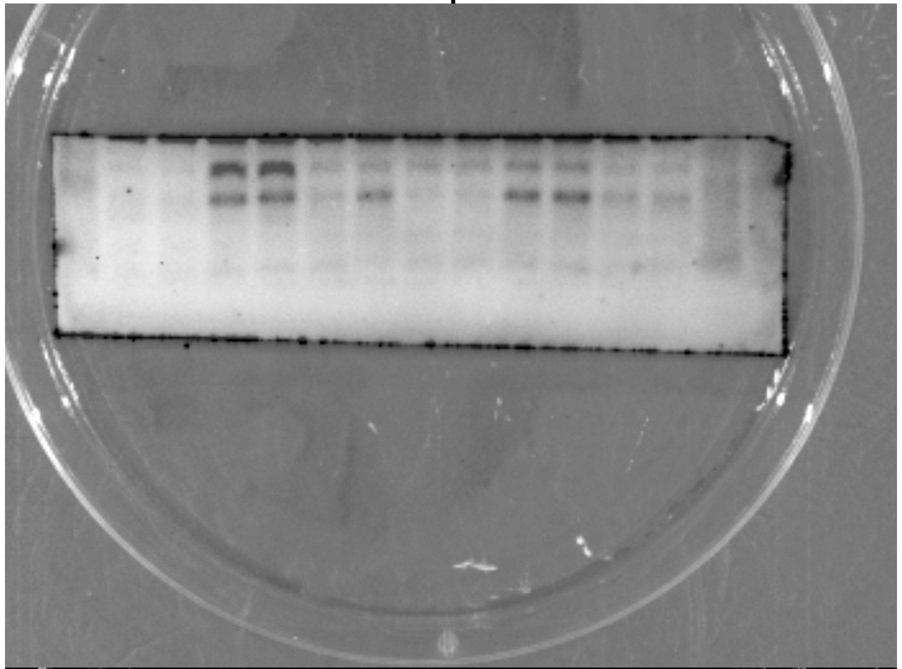

Colormeritric mode

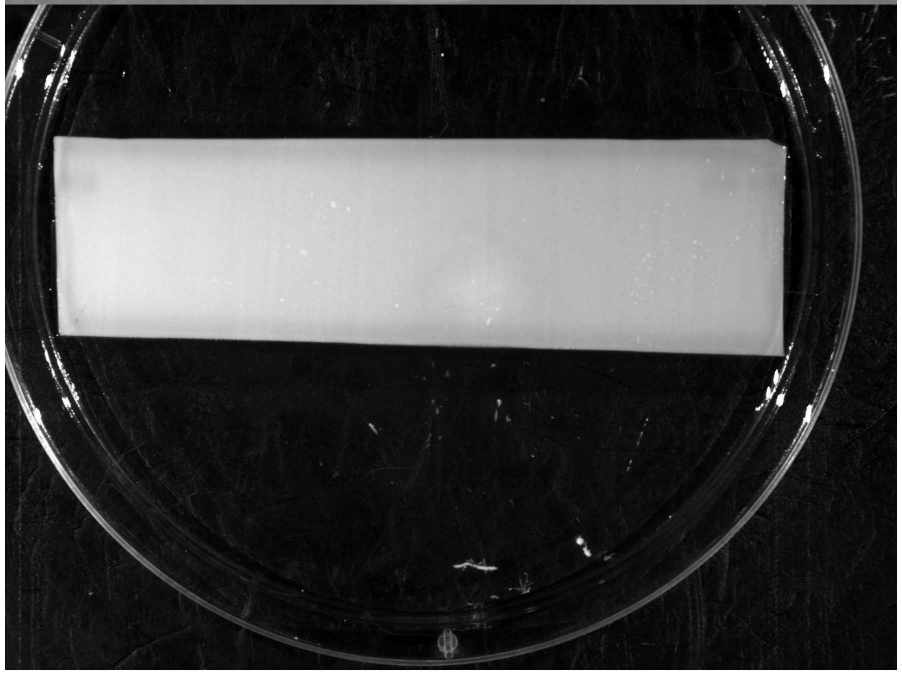

Figure-4 3D SLC7A11

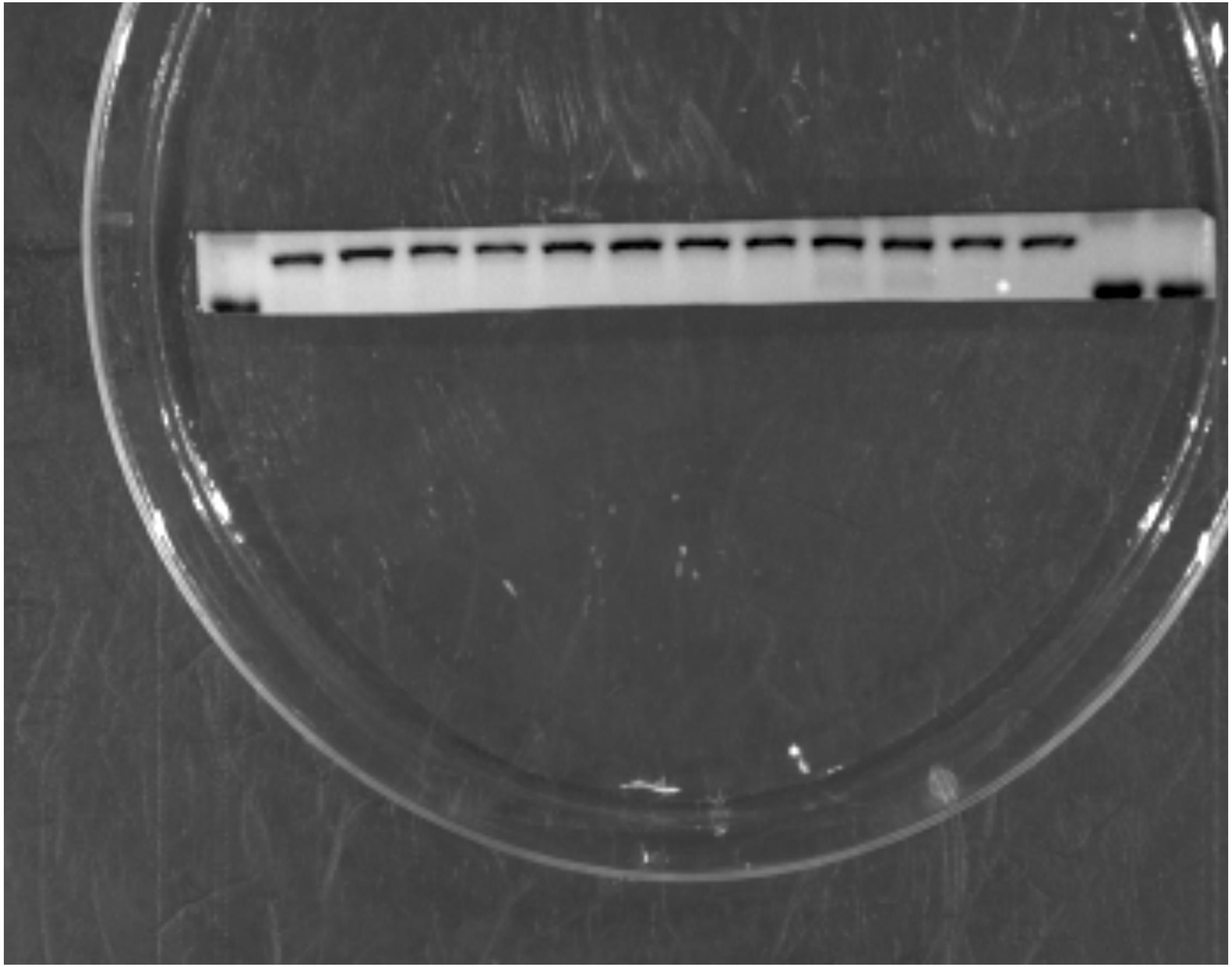

Figure-4 3D GAP43

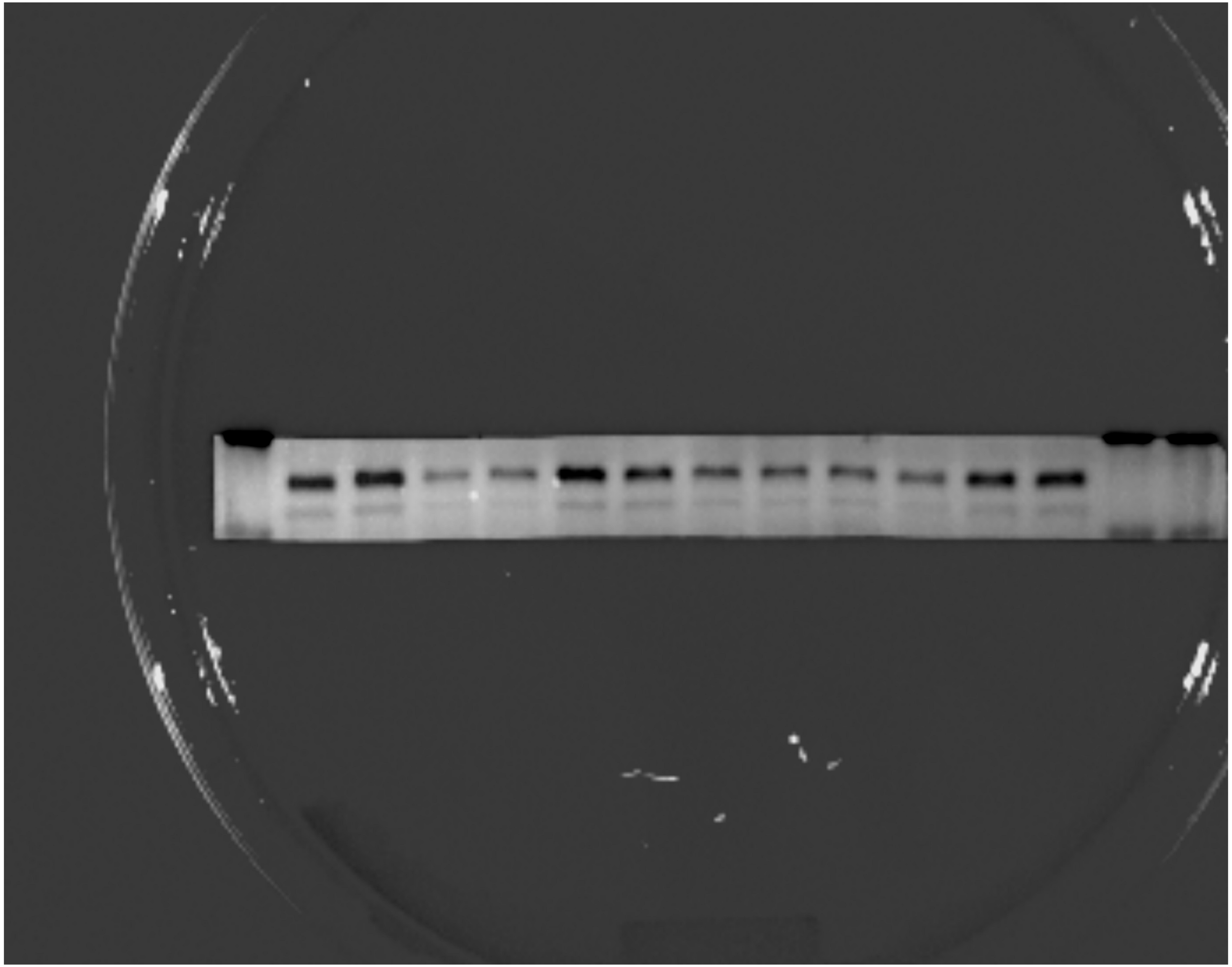

Figure-4 3D RIP1, FSP1 and VDAC are all from one membrane, the blot of VDAC was stripped to show GAPDH

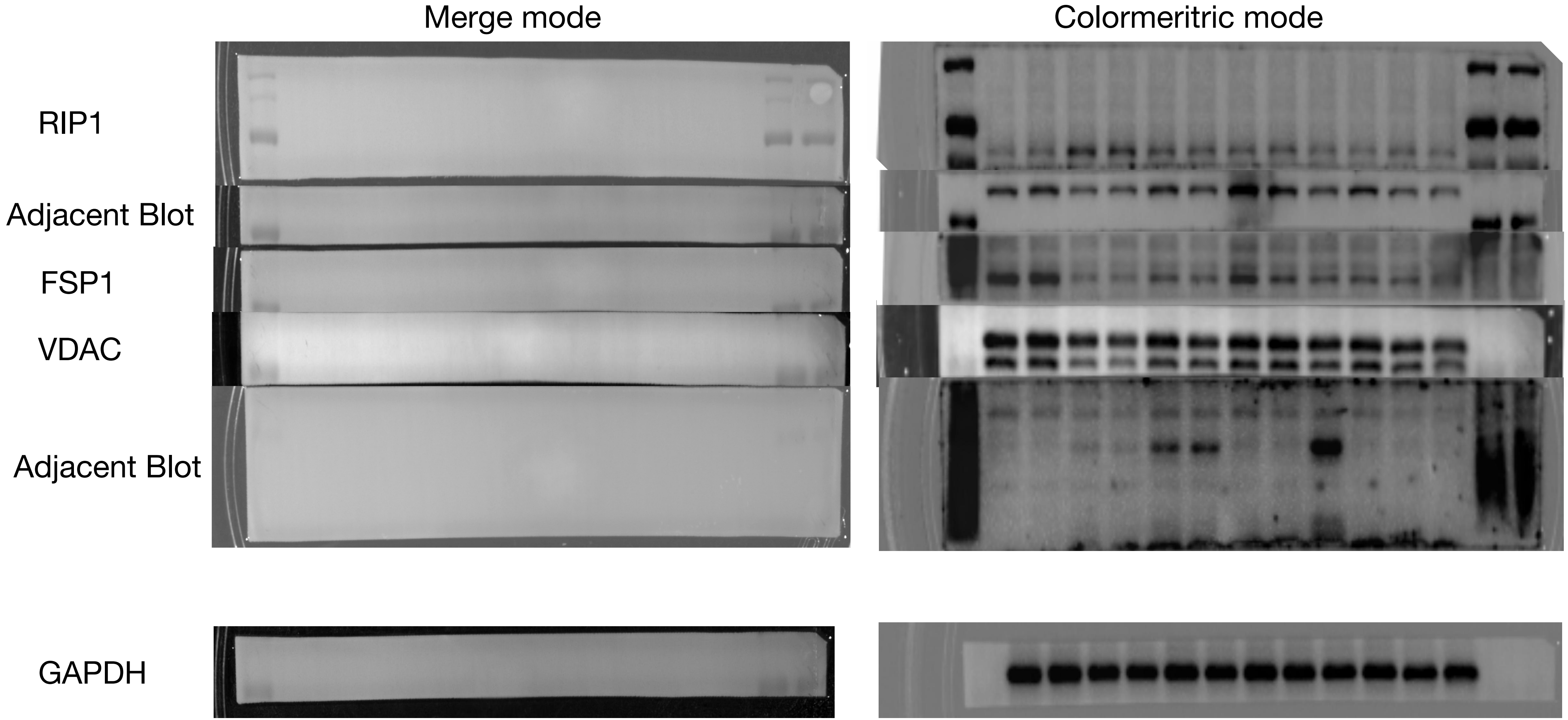

Figure-4 3D All orignal pictures in the spliced pictures are shown

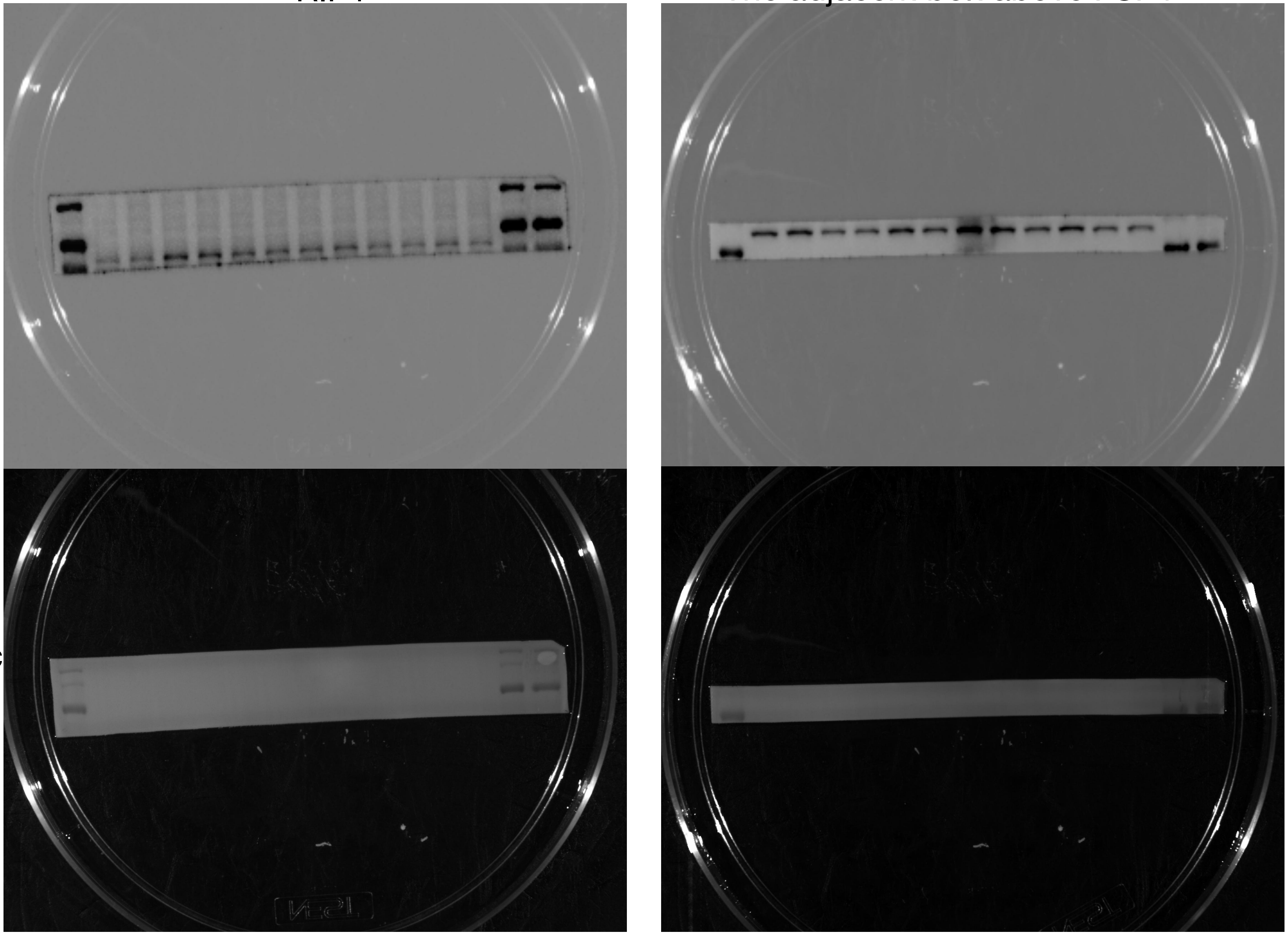

Figure-4 3D All orignal pictures in the spliced pictures are shown

FSP1

The adjacent bolt below VDAC(GAPDH)

Merge mode

Colormeritric mode

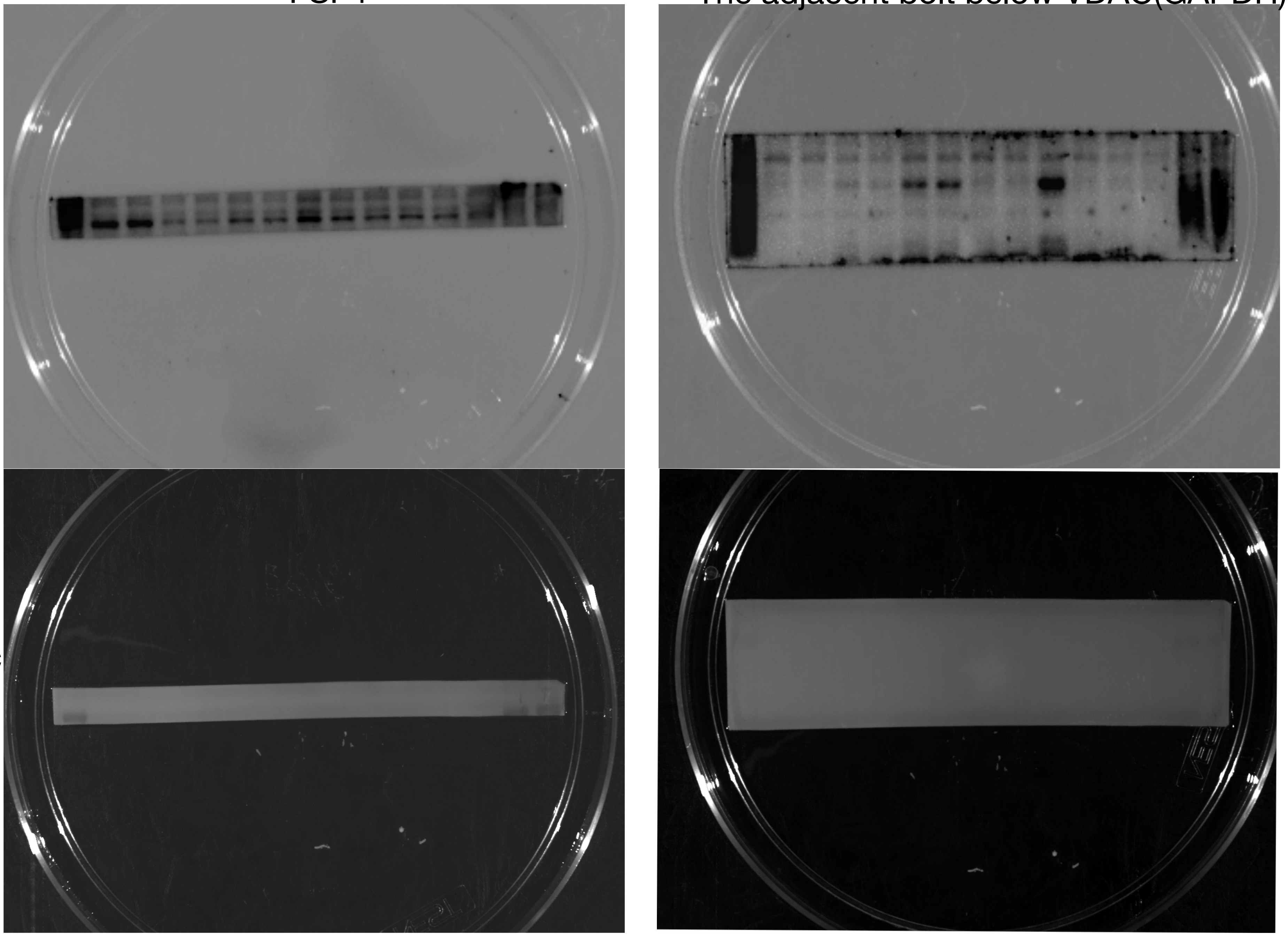

Figure-4 3D All orignal pictures in the spliced pictures are shown

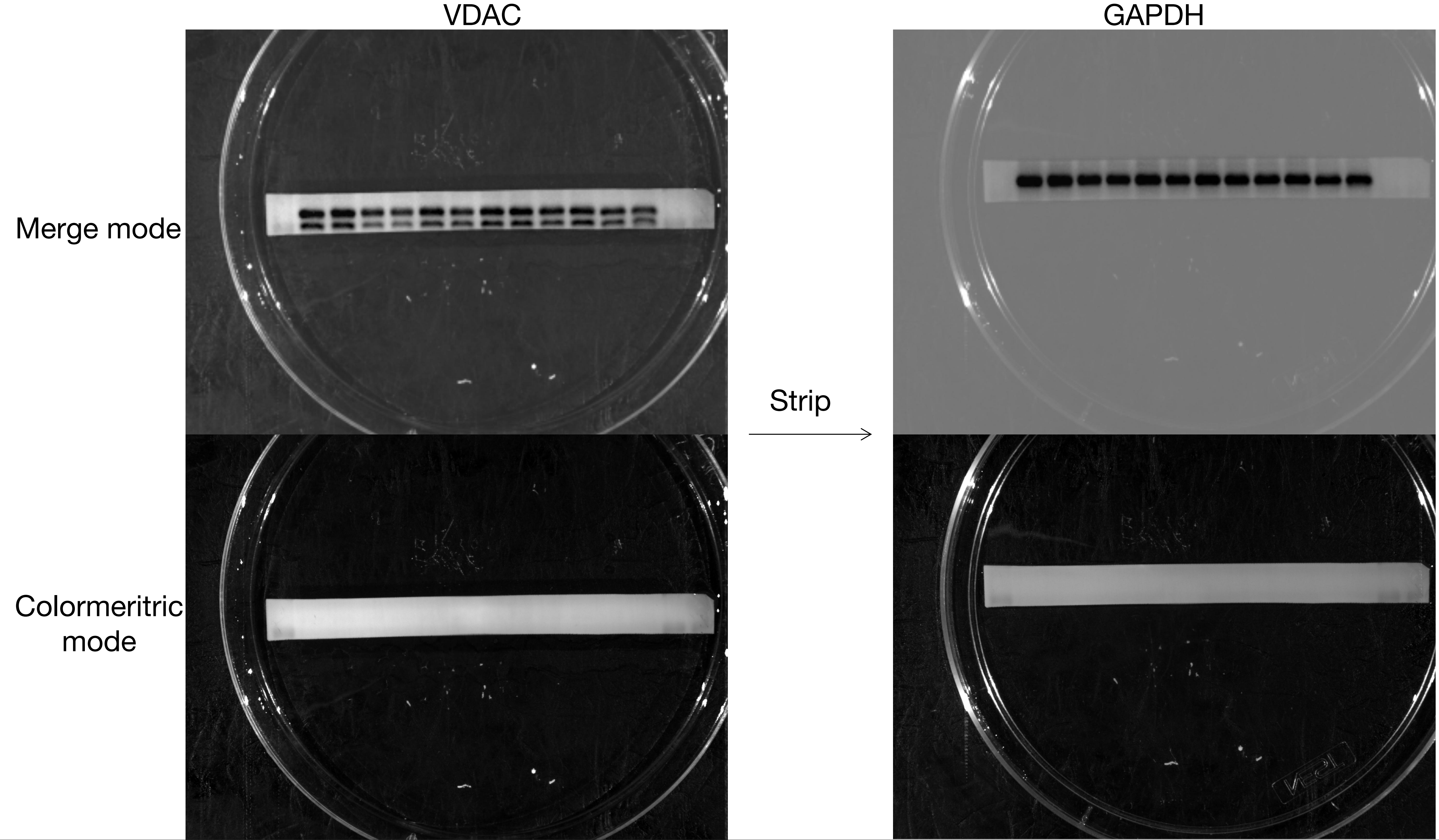

Figure-4 7D FSP-1 and VDAC are from one membrane and are adjacent

Merge mode

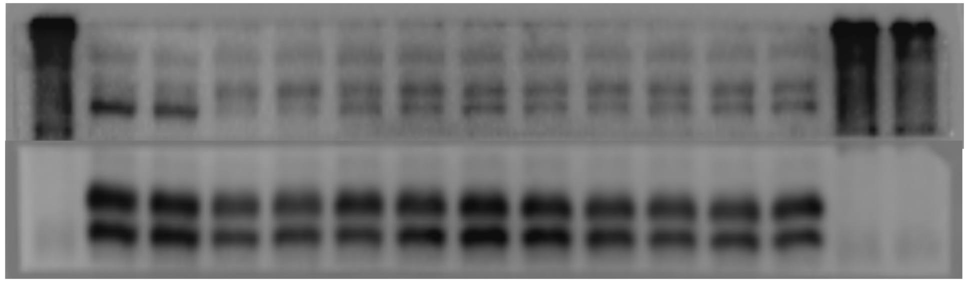

Colormeritric mode

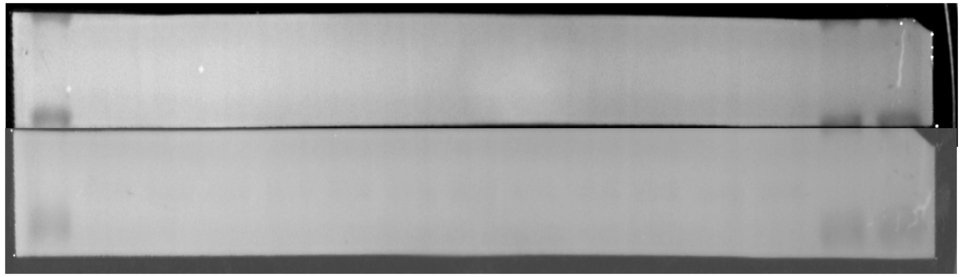

FSP1

VDAC

Figure-4 7D All orignal pictures in the spliced pictures are shown

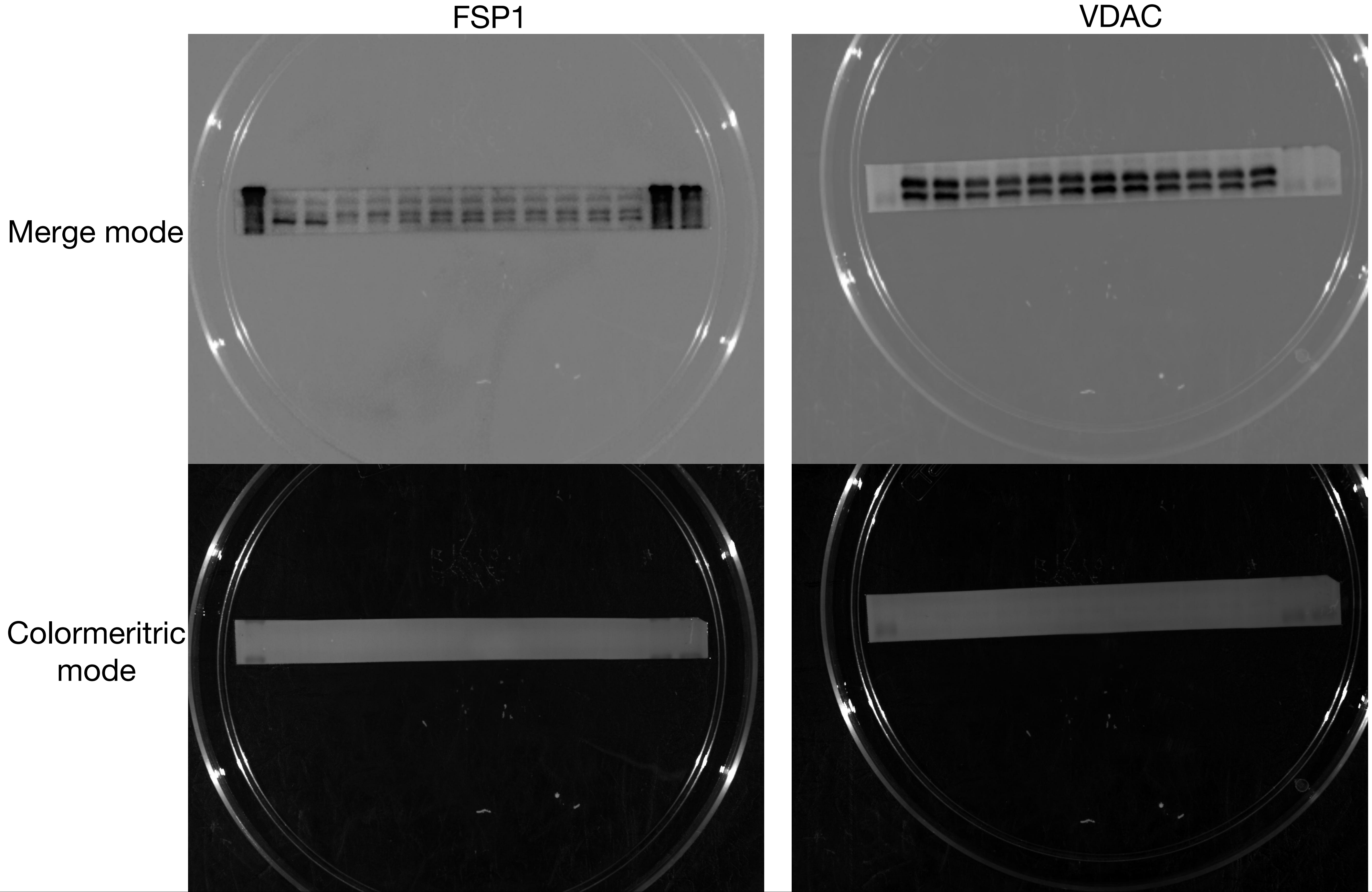

Figure-4 7D RIP1

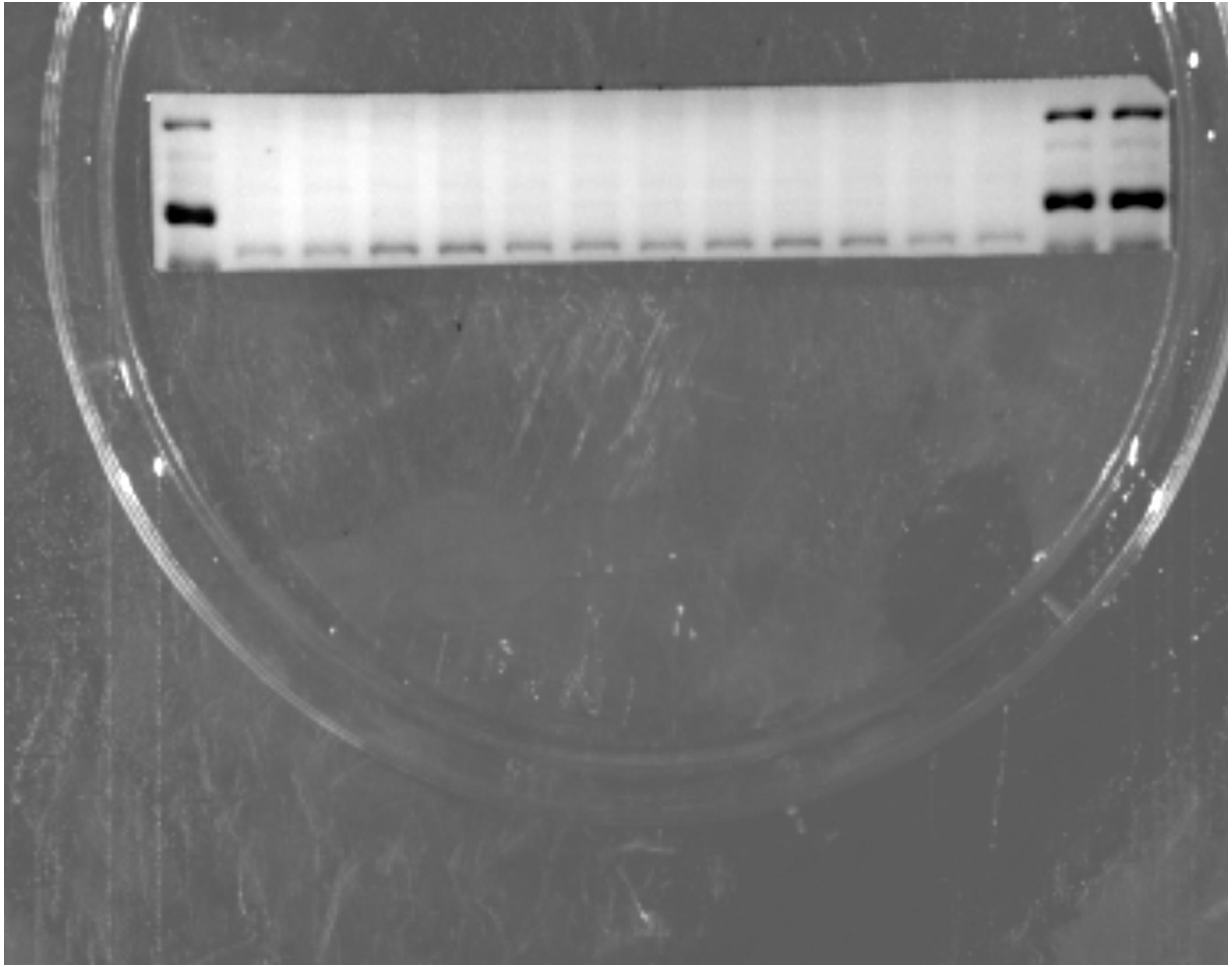

Figure-4 7D GAP43

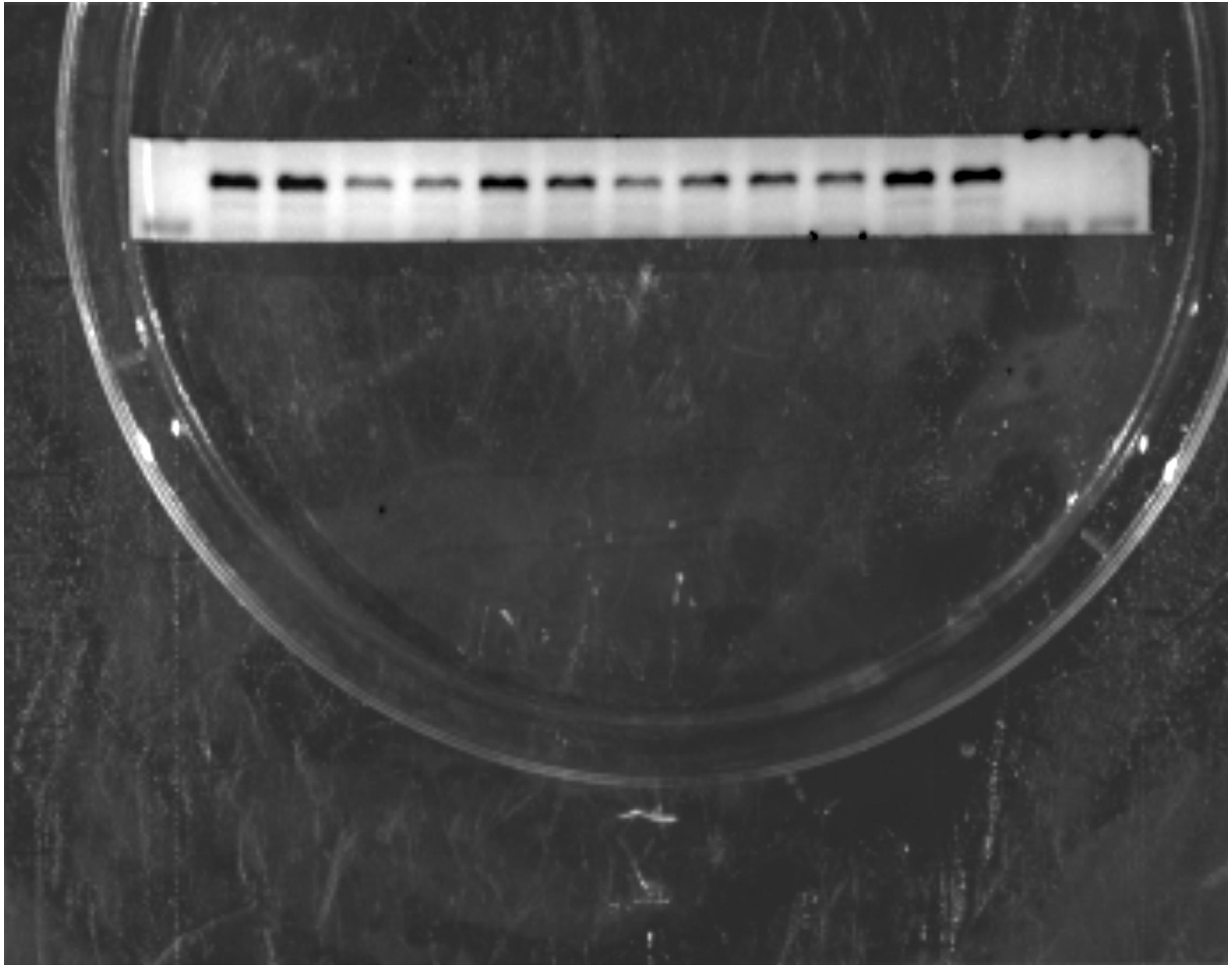

Figure-4 7D TFR1 SLC7A11 and NeuN are all from one membrane

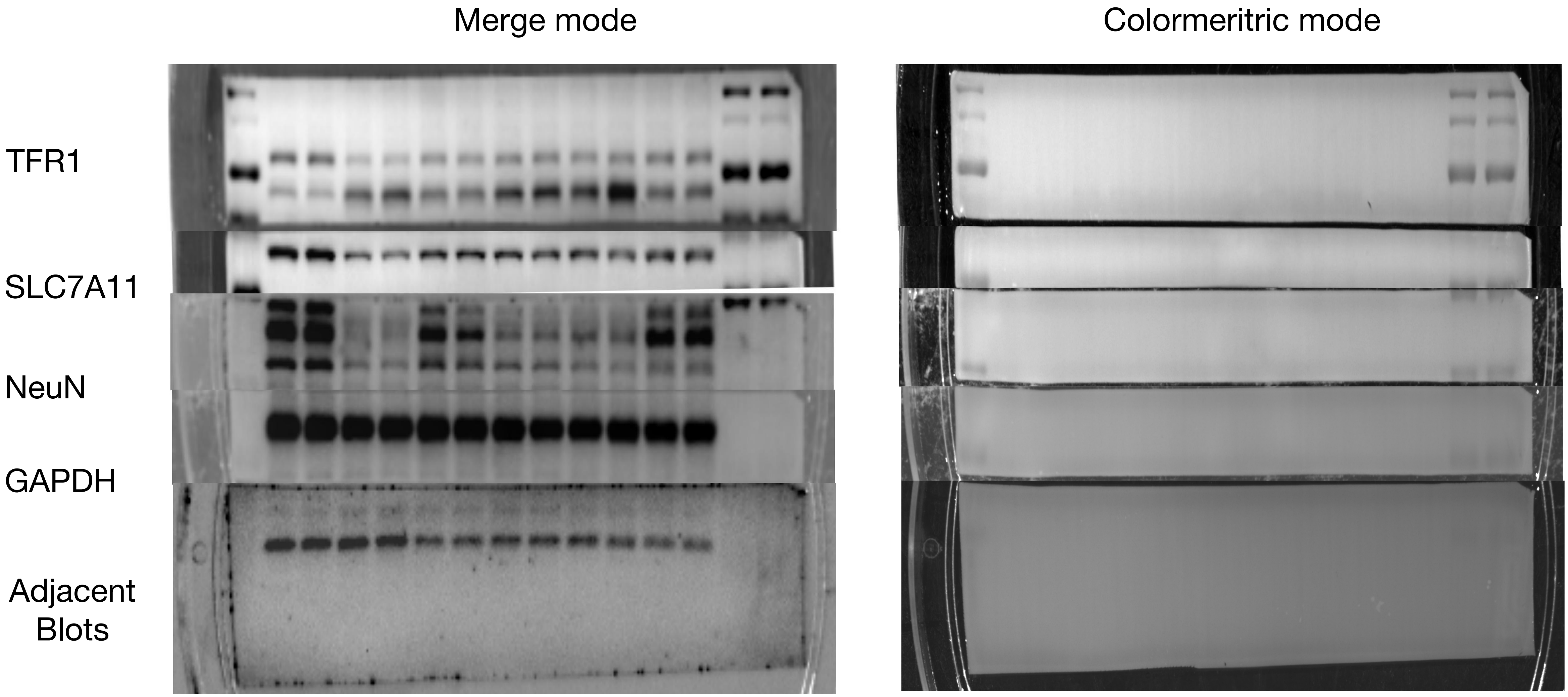

Figure-4 7D All orignal pictures in the spliced pictures are shown

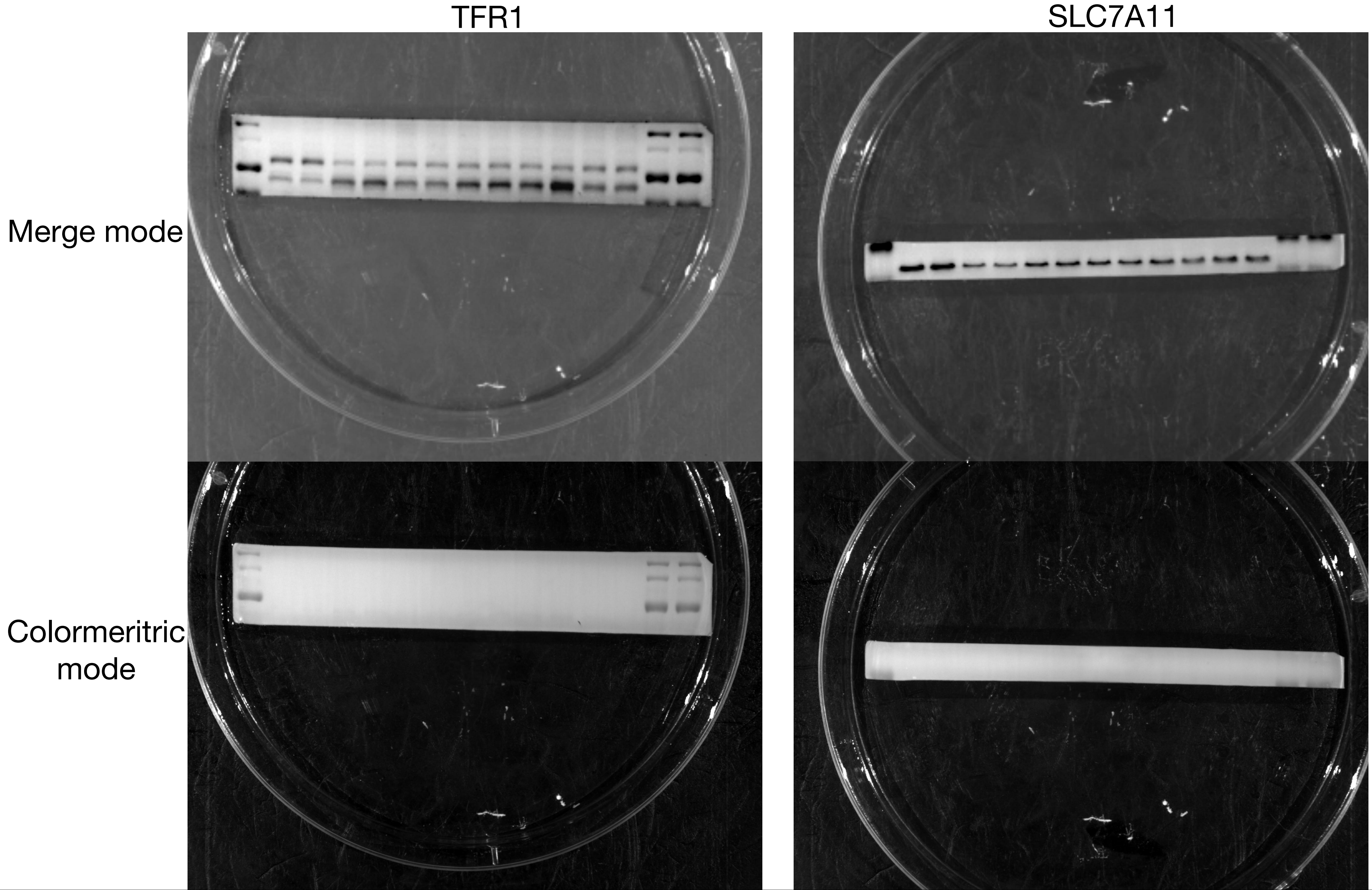

Figure-4 7D All orignal pictures in the spliced pictures are shown

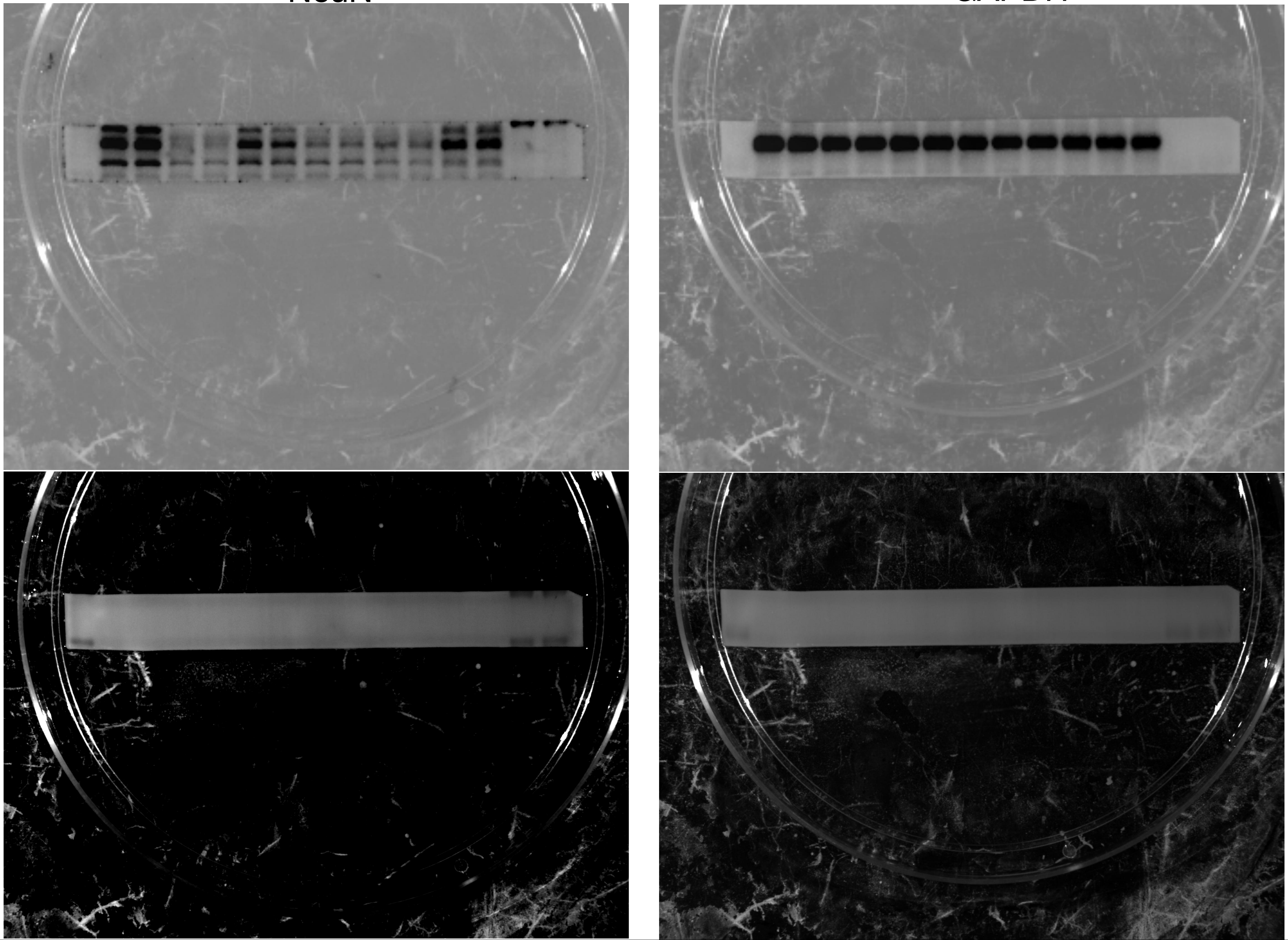

Figure-4 7D All orignal pictures in the spliced pictures are shown

The adjacent blots below GAPDH

Merge mode

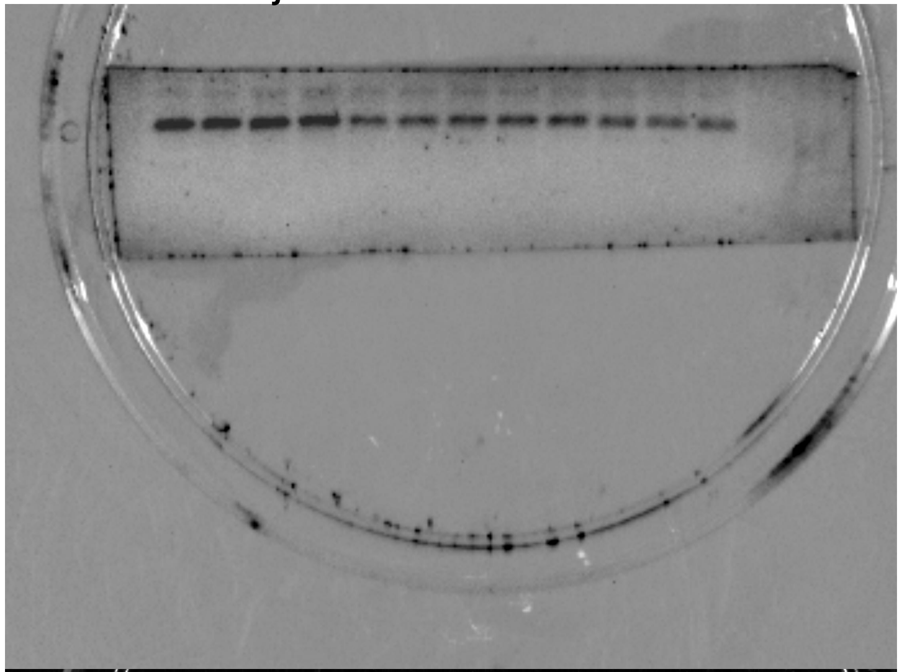

Colormeritric mode

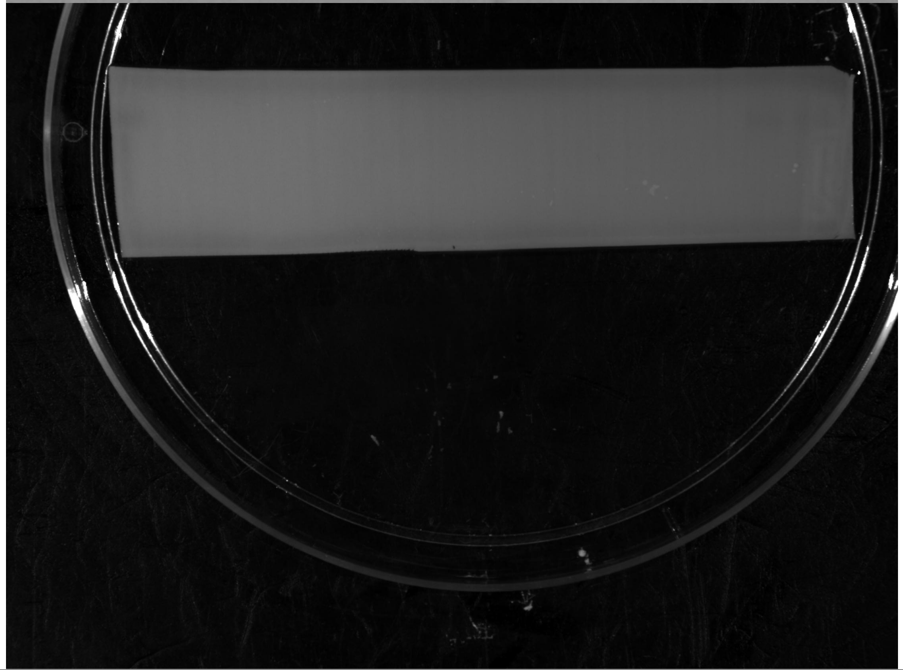

Figure-4 7D TF

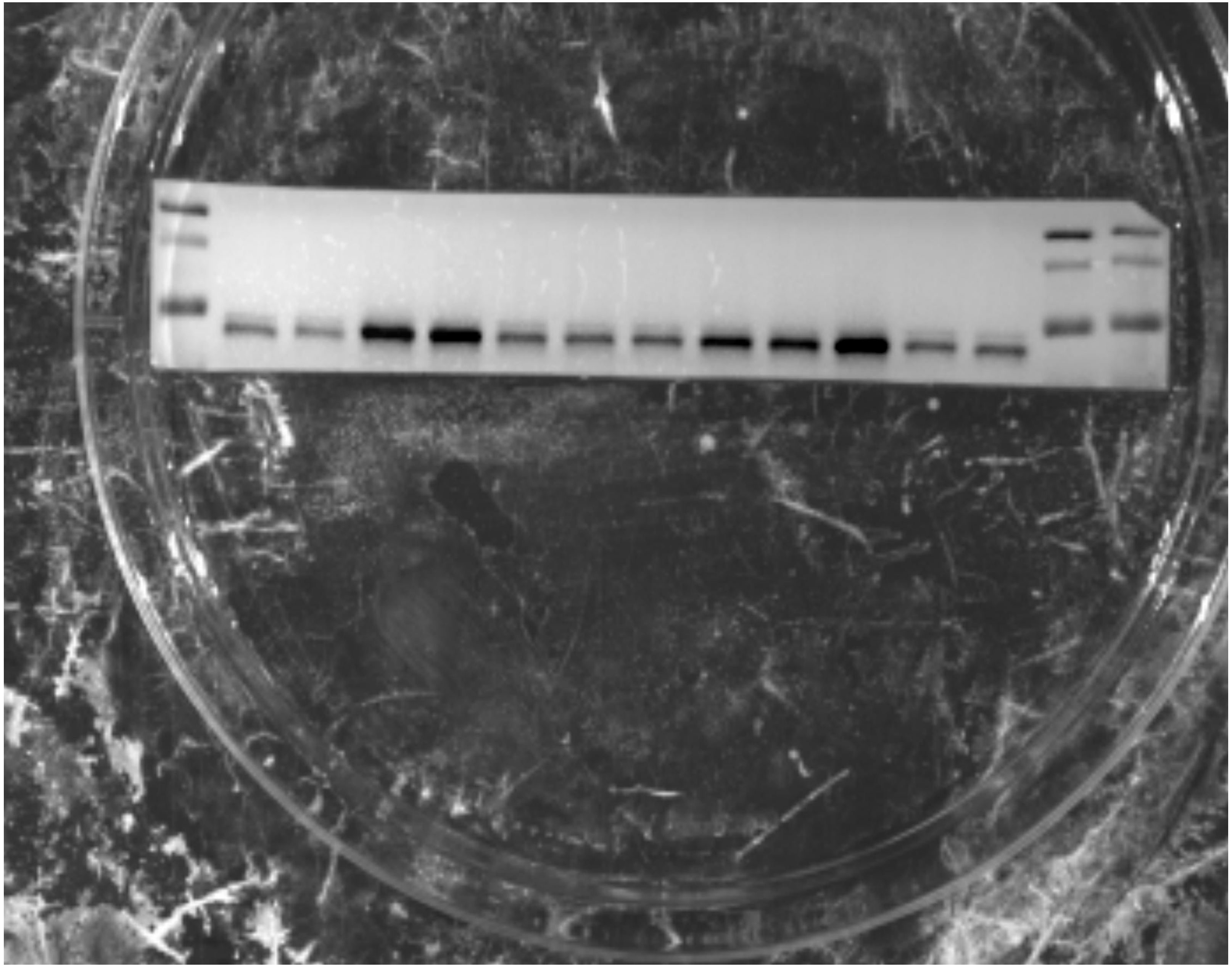

Figure-4 7D Cox2

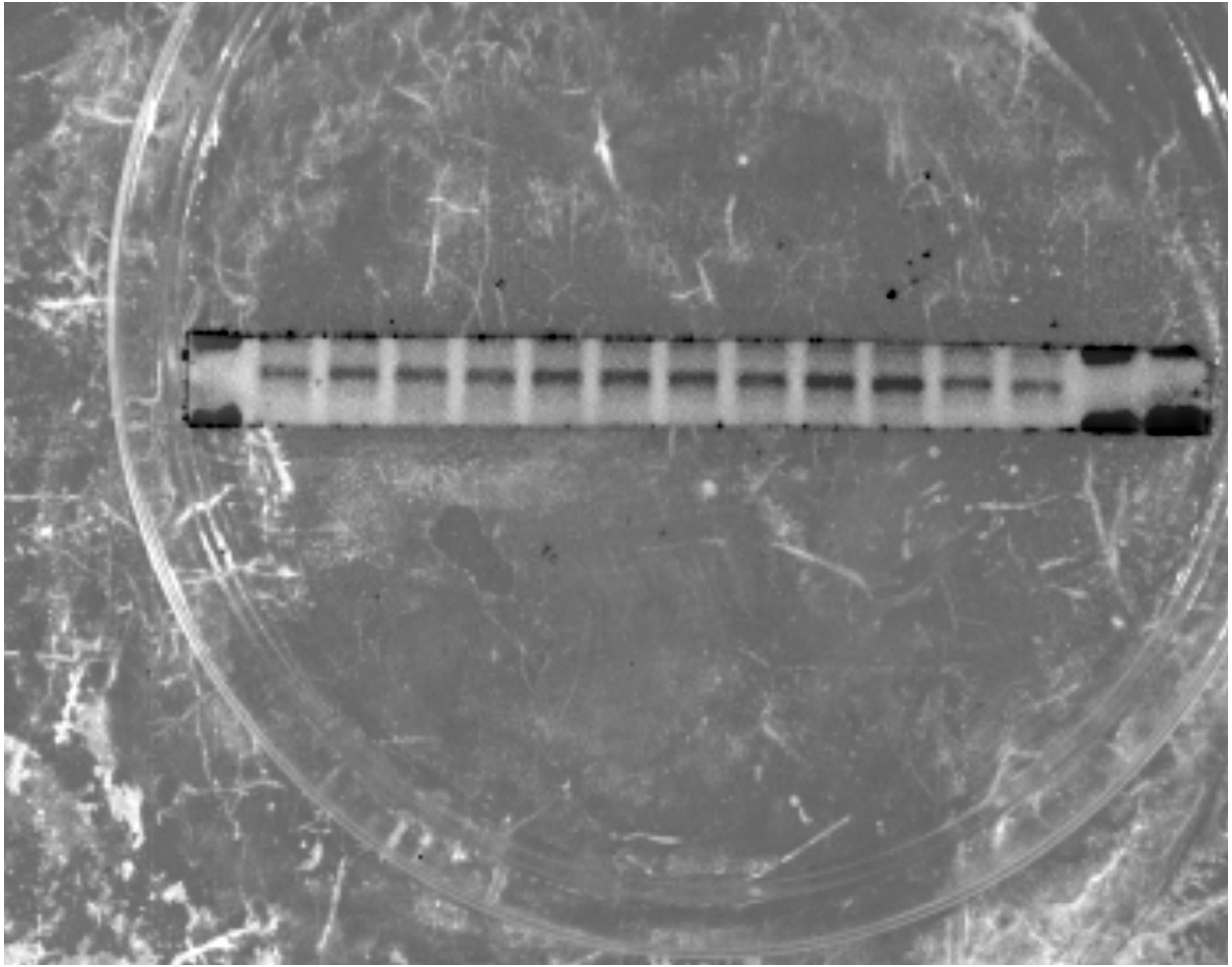

Figure-4 7D GAPDH

Merge mode

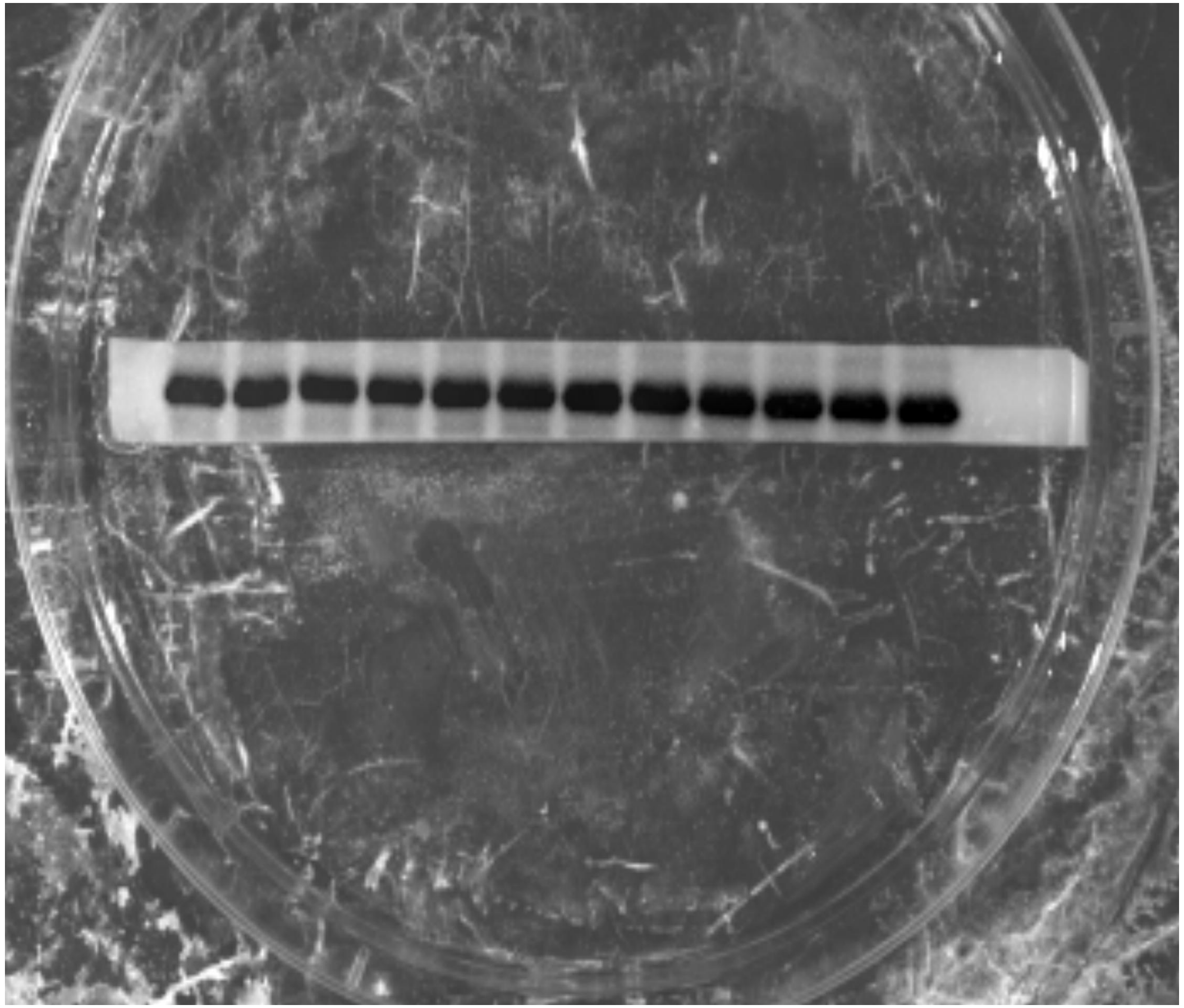

Colormeritric mode

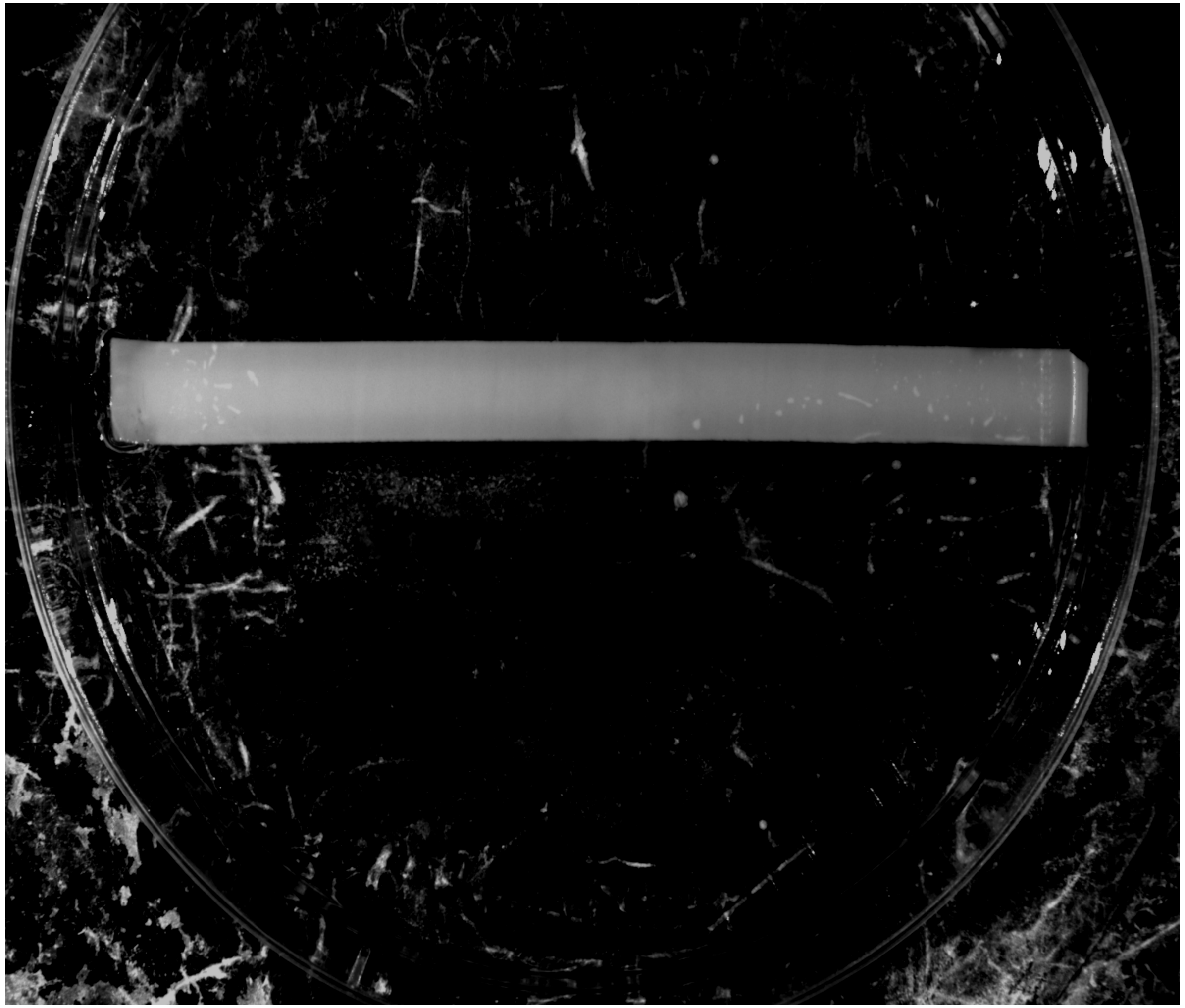

Figure-4 7D Cleaved Caspase3 and its adjacent GAPDH

Cleaved Caspase3

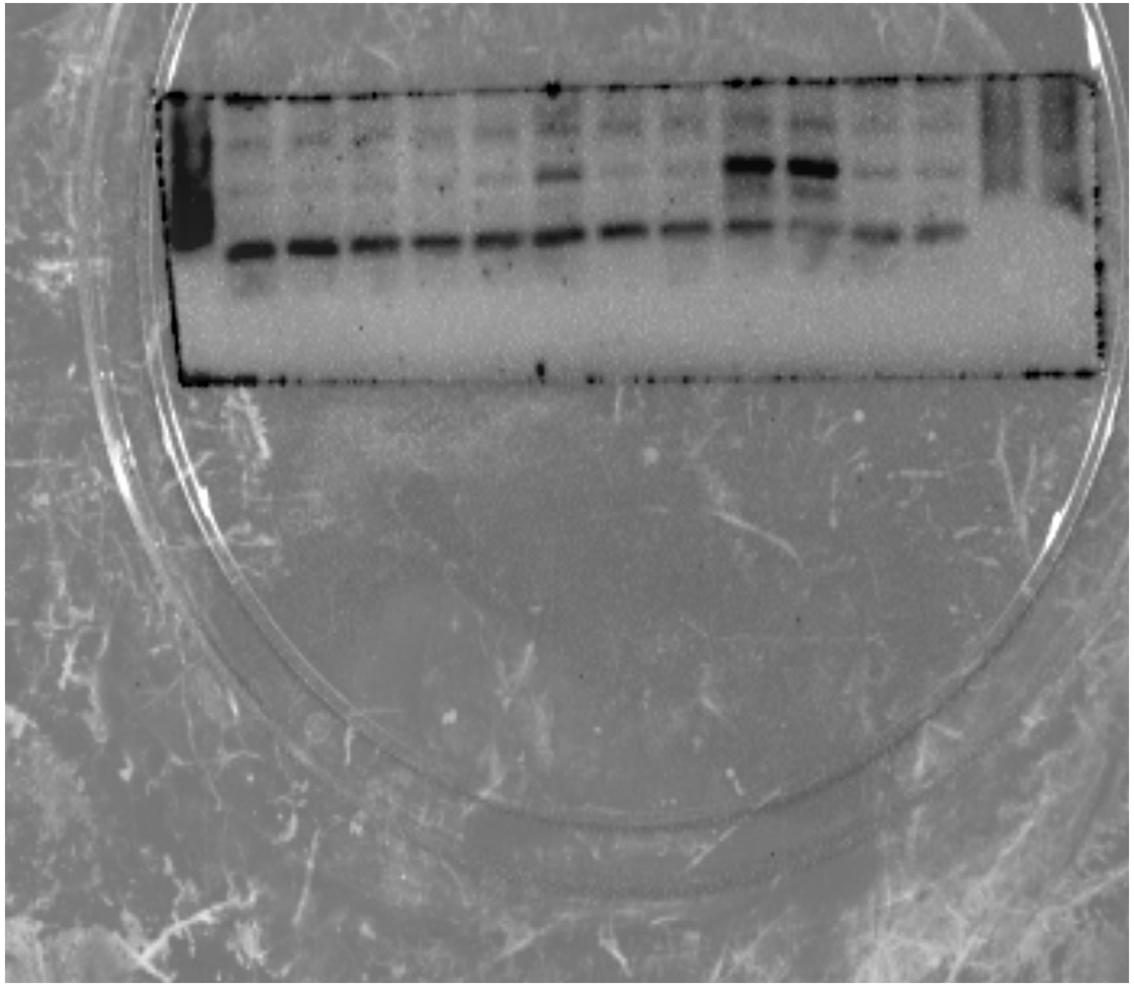

Cleaved Caspase3 and its adjacent GAPDH

Merge mode

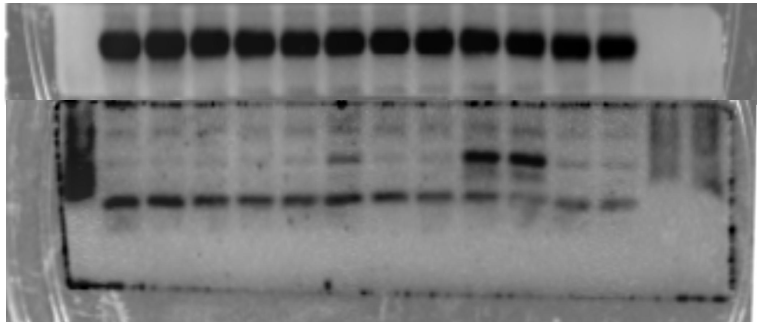

Colormeritric mode

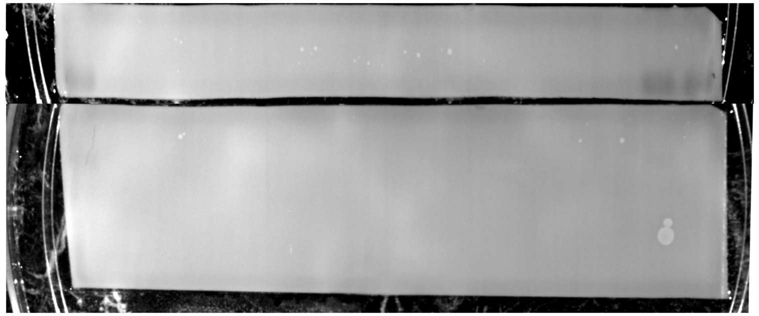

Figure-4 7D All orignal pictures in the spliced pictures are shown

The adjacent GAPDH

Cleaved Caspase3

Merge mode

Colormeritric mode

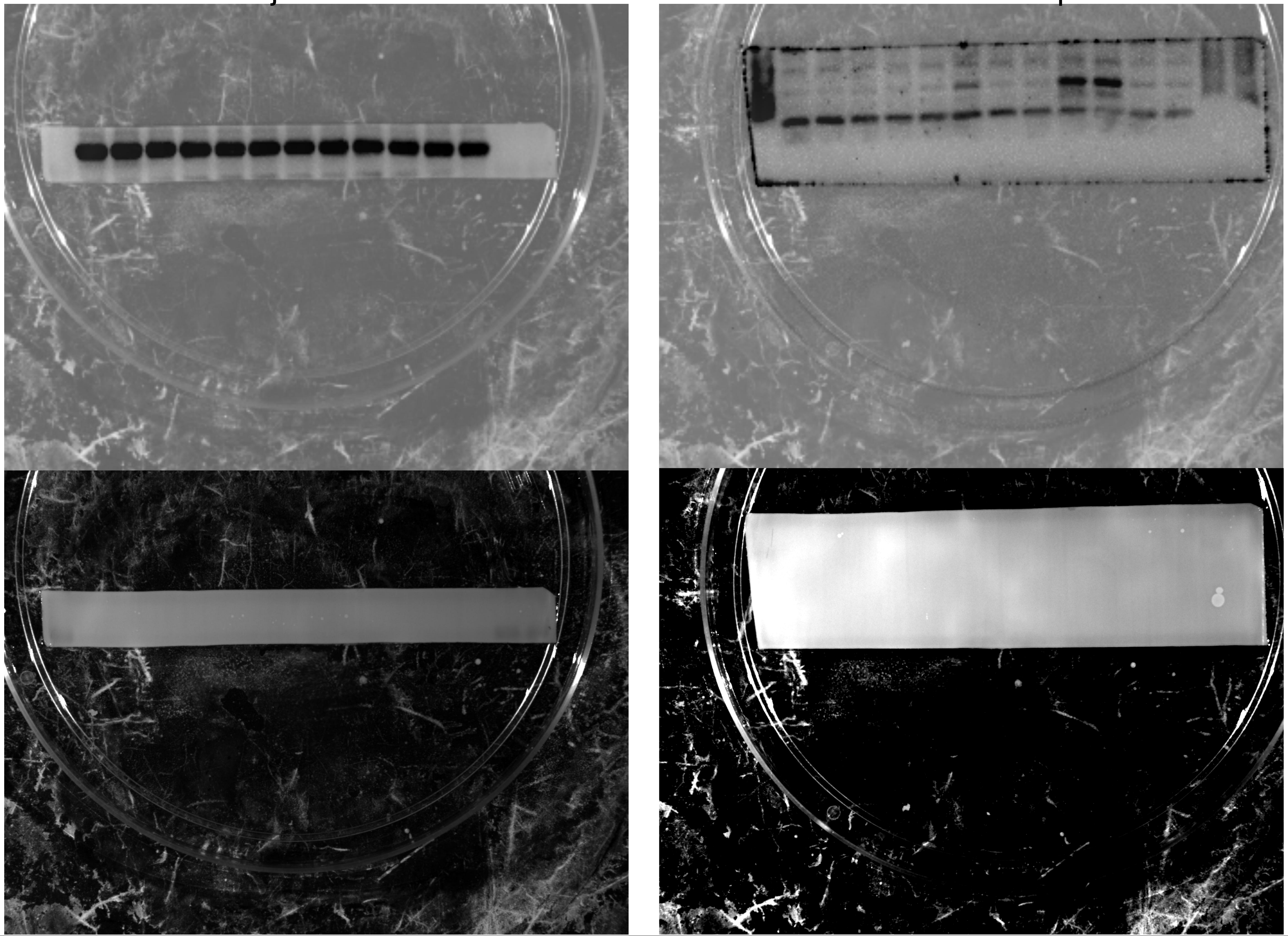

Supplementary Figure-8A 3D GFAP

Merge mode

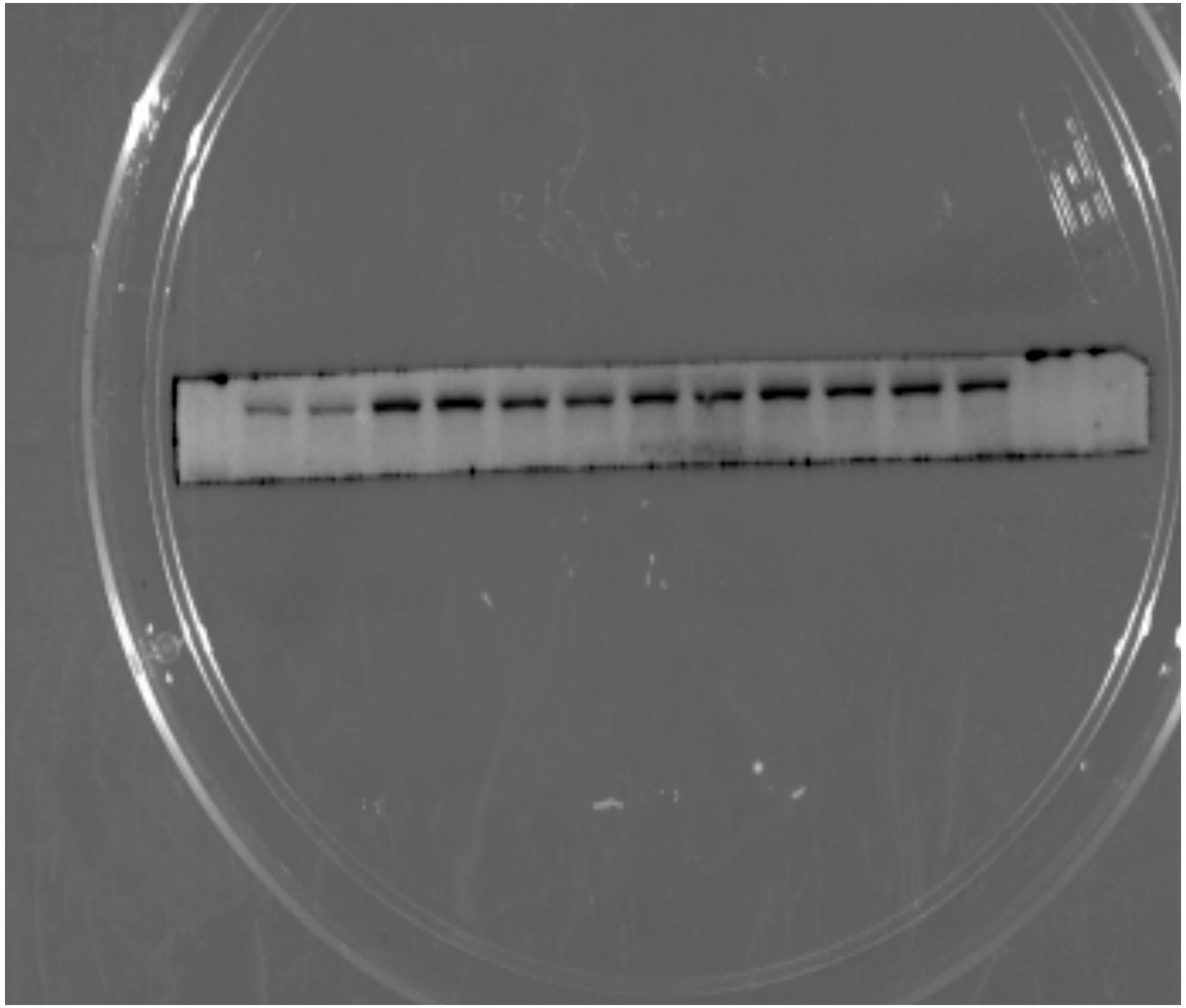

Colormeritric mode

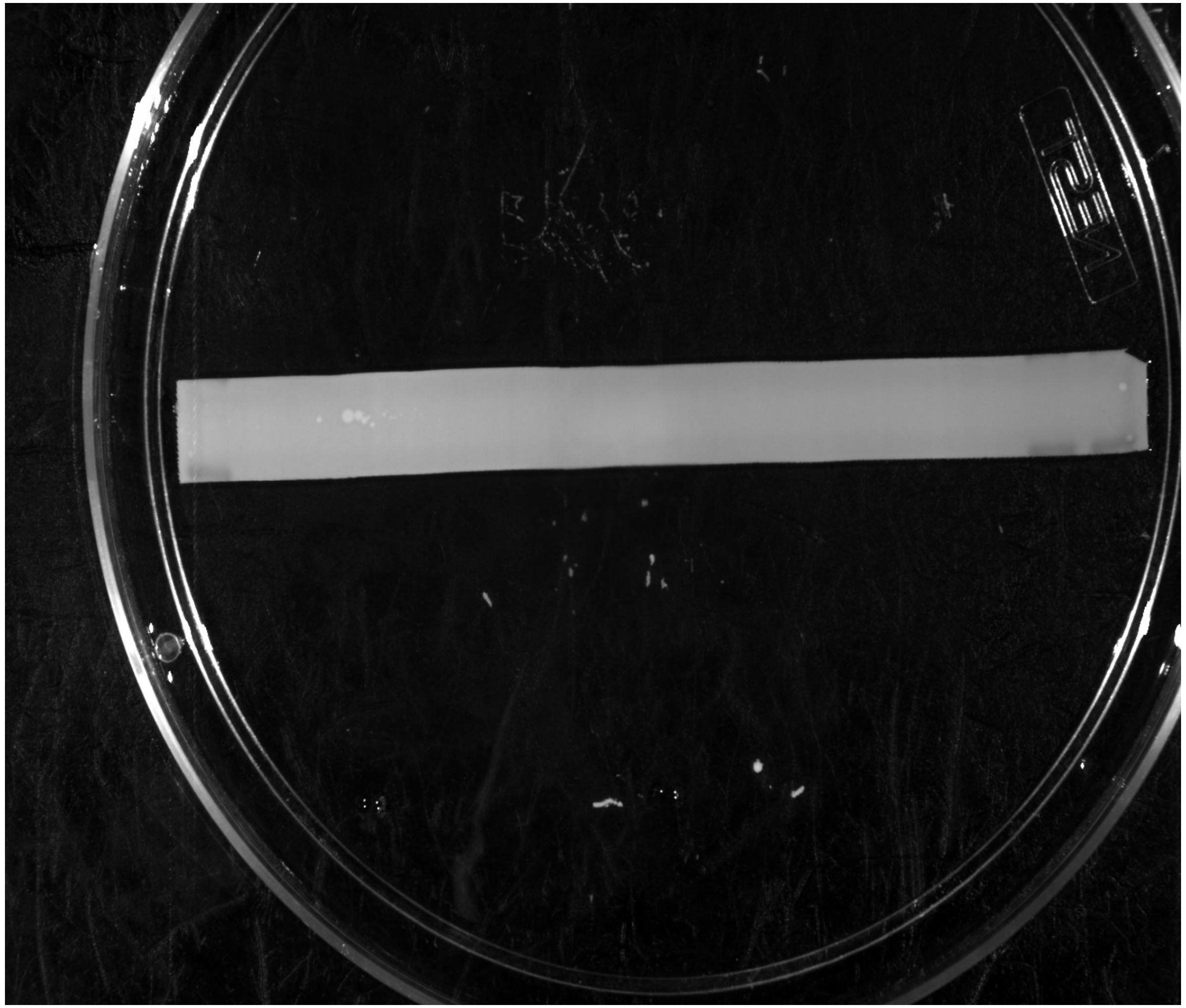

Supplementary Figure-8A 3D Iba-1

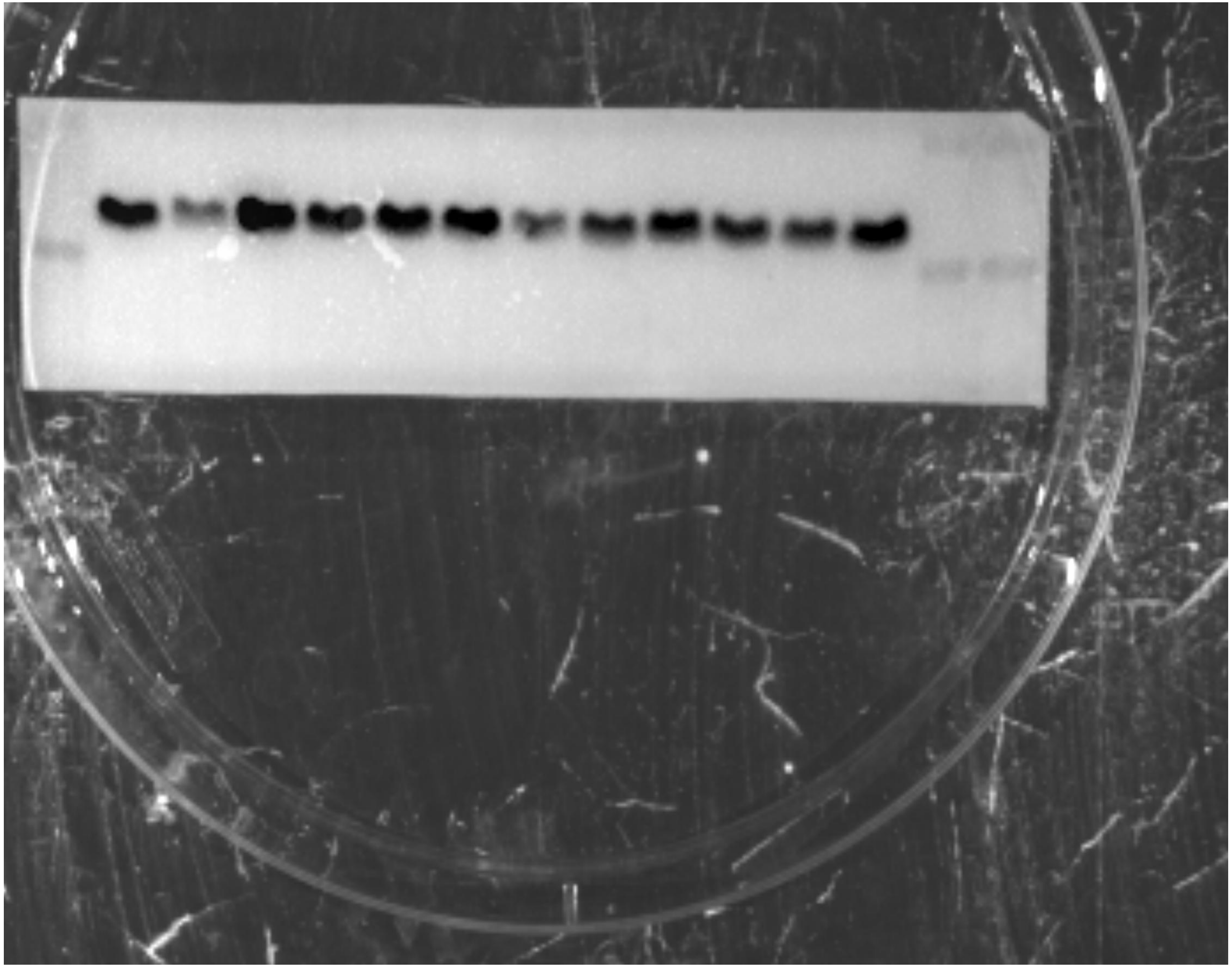

Supplementary Figure-8A 3D CD31

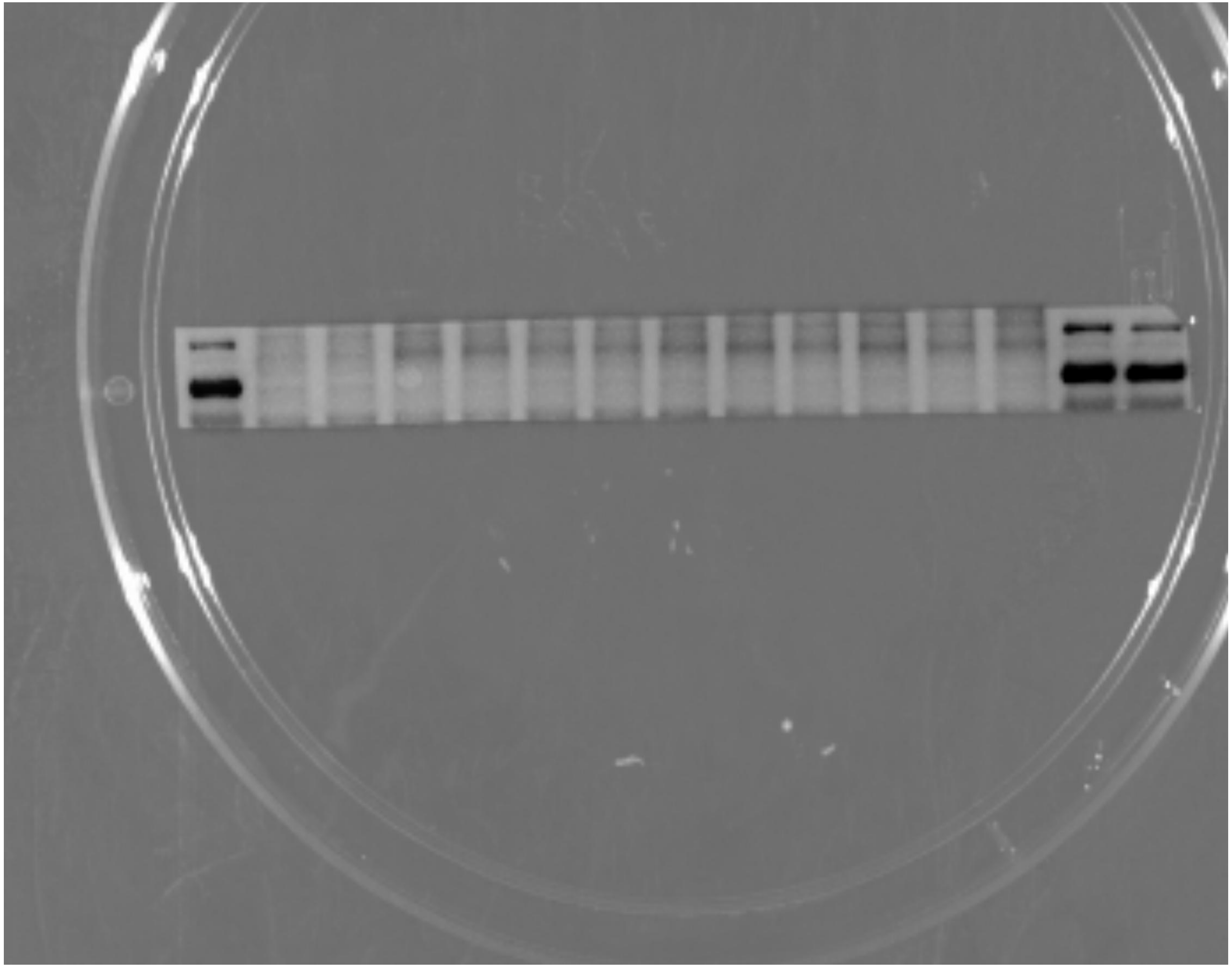

Supplementary Figure-8A 3D VEGF

Merge mode

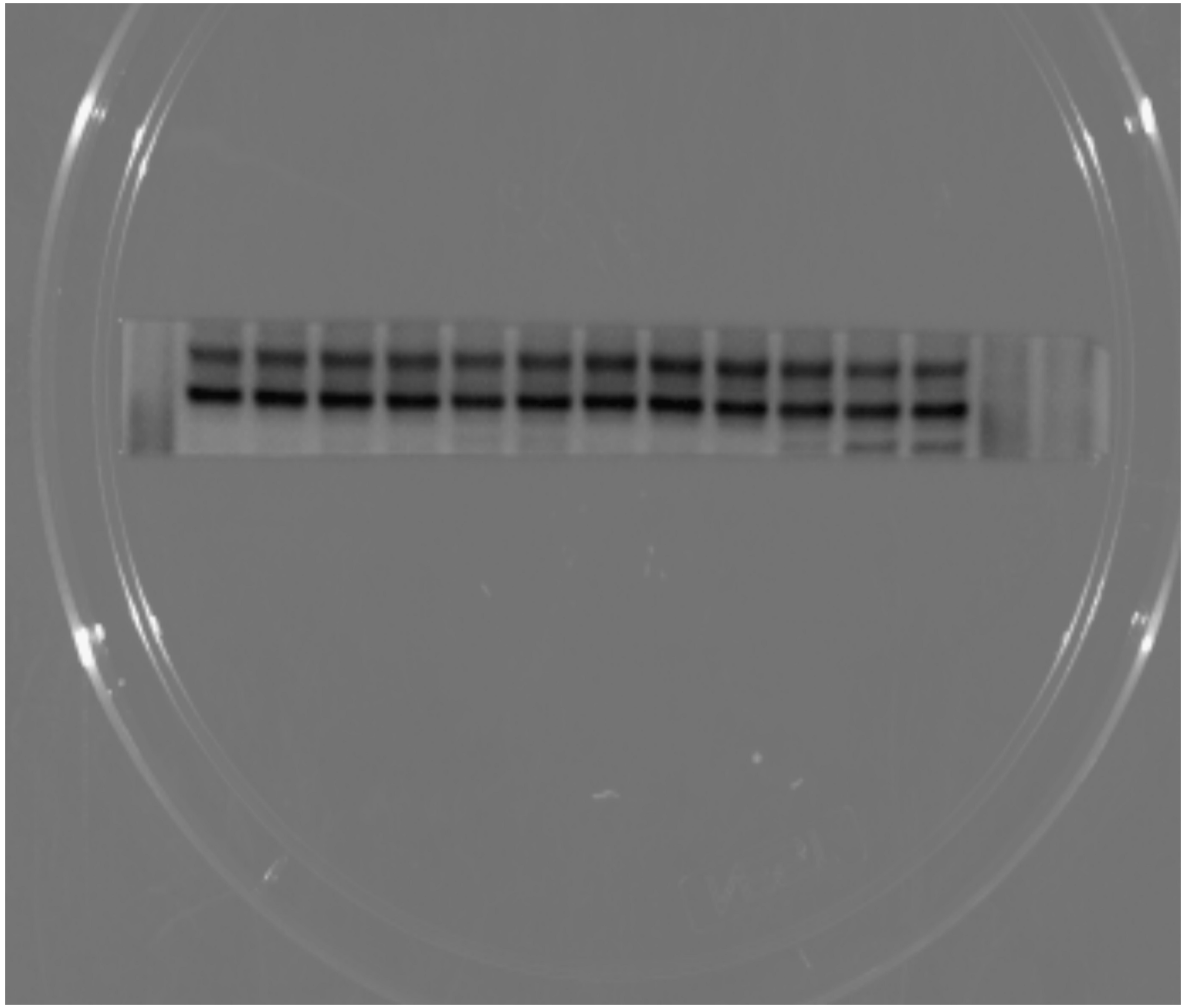

Colormeritric mode

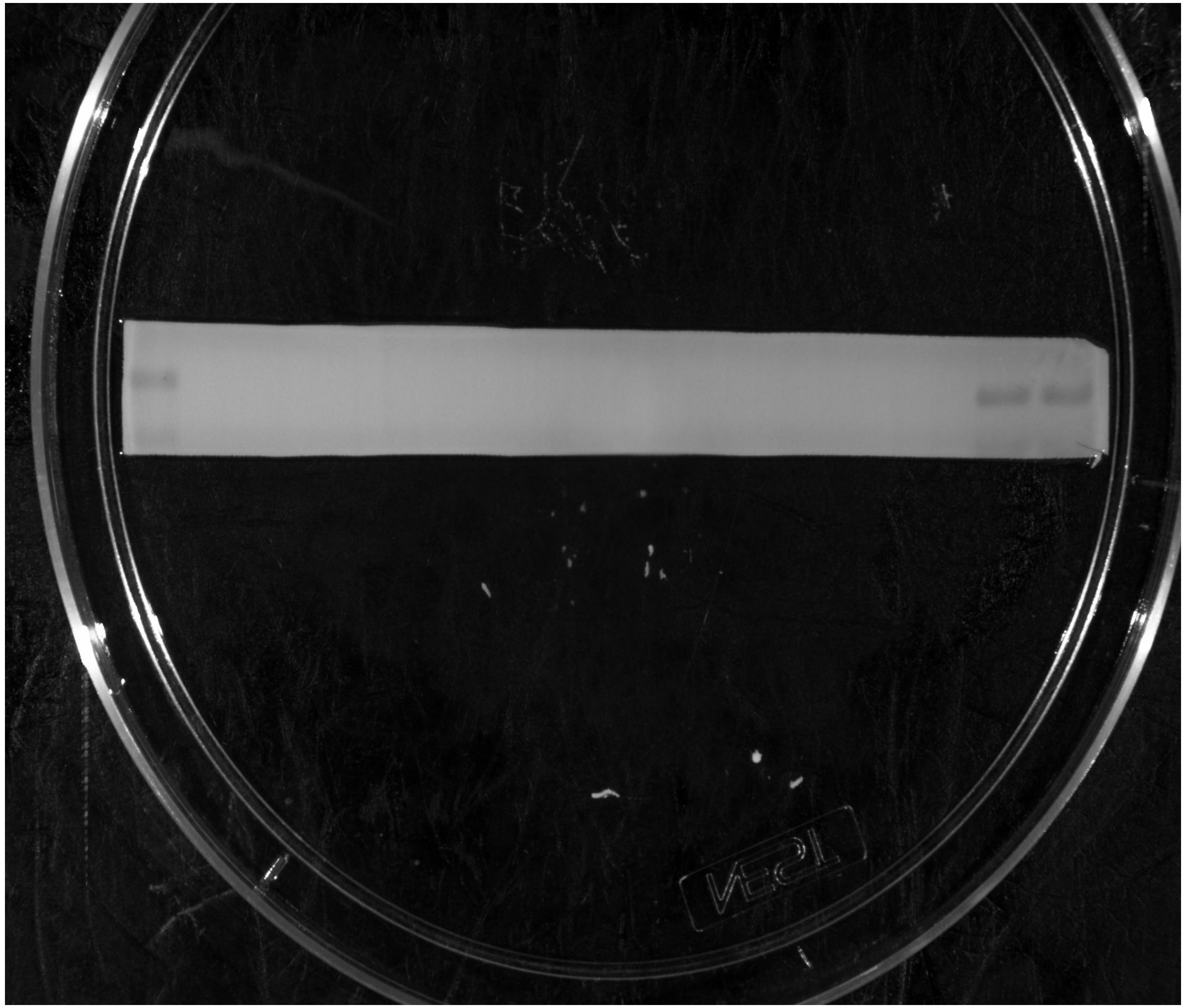

Supplementary Figure-8A 3D GAPDH

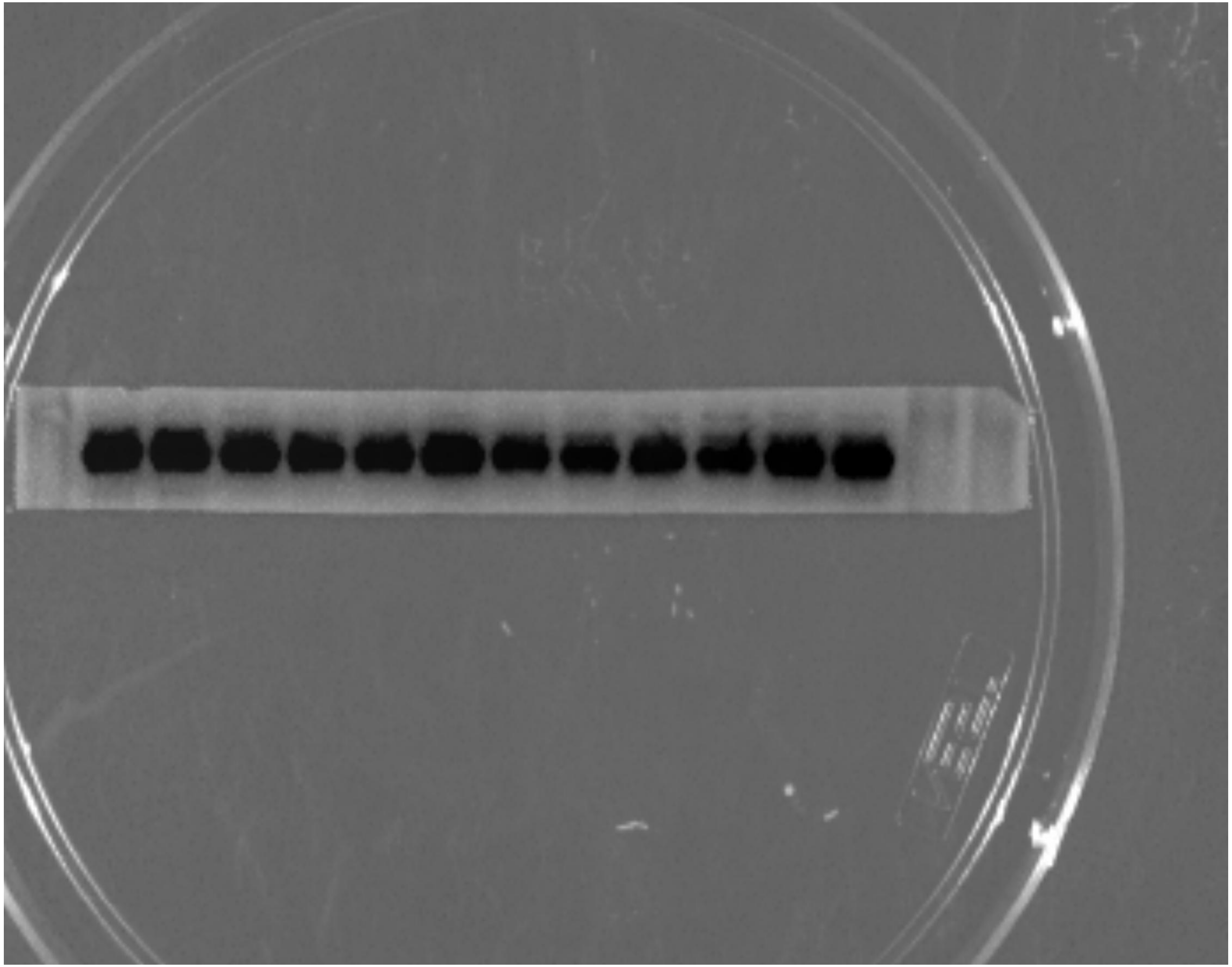

Supplementary Figure-8A 7D GFAP

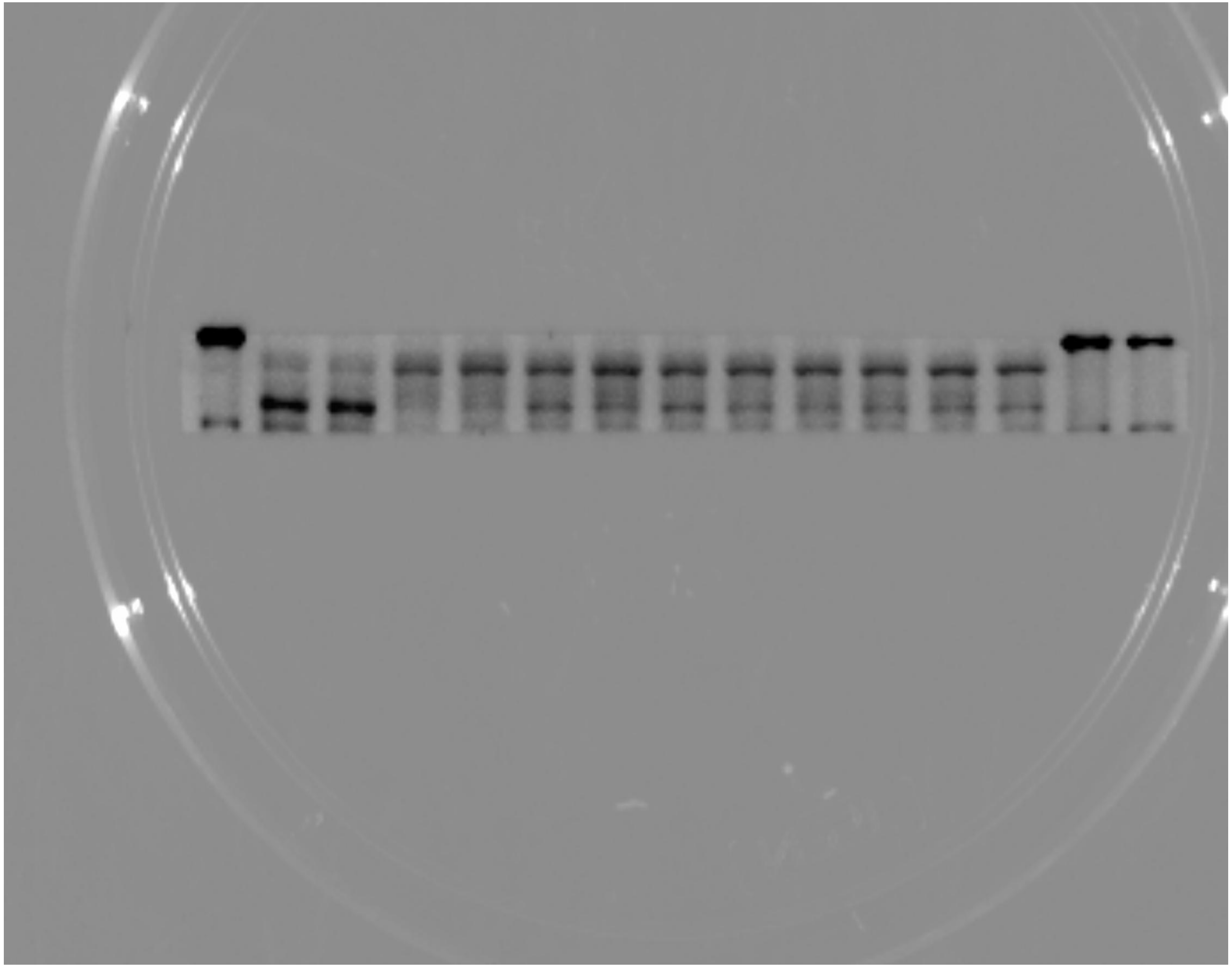

Supplementary Figure-8A 7D Iba-1

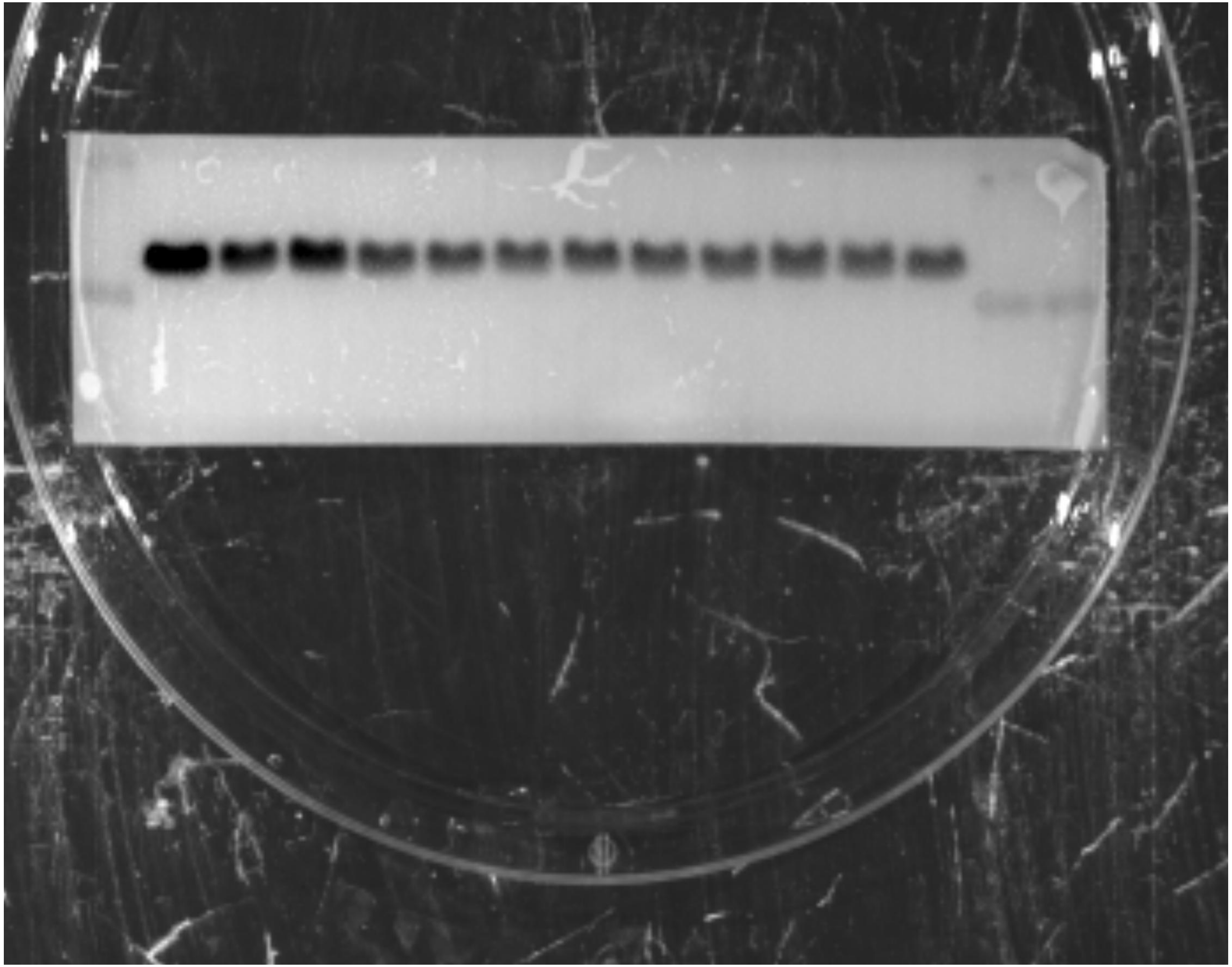

Supplementary Figure-8A 7D CD31

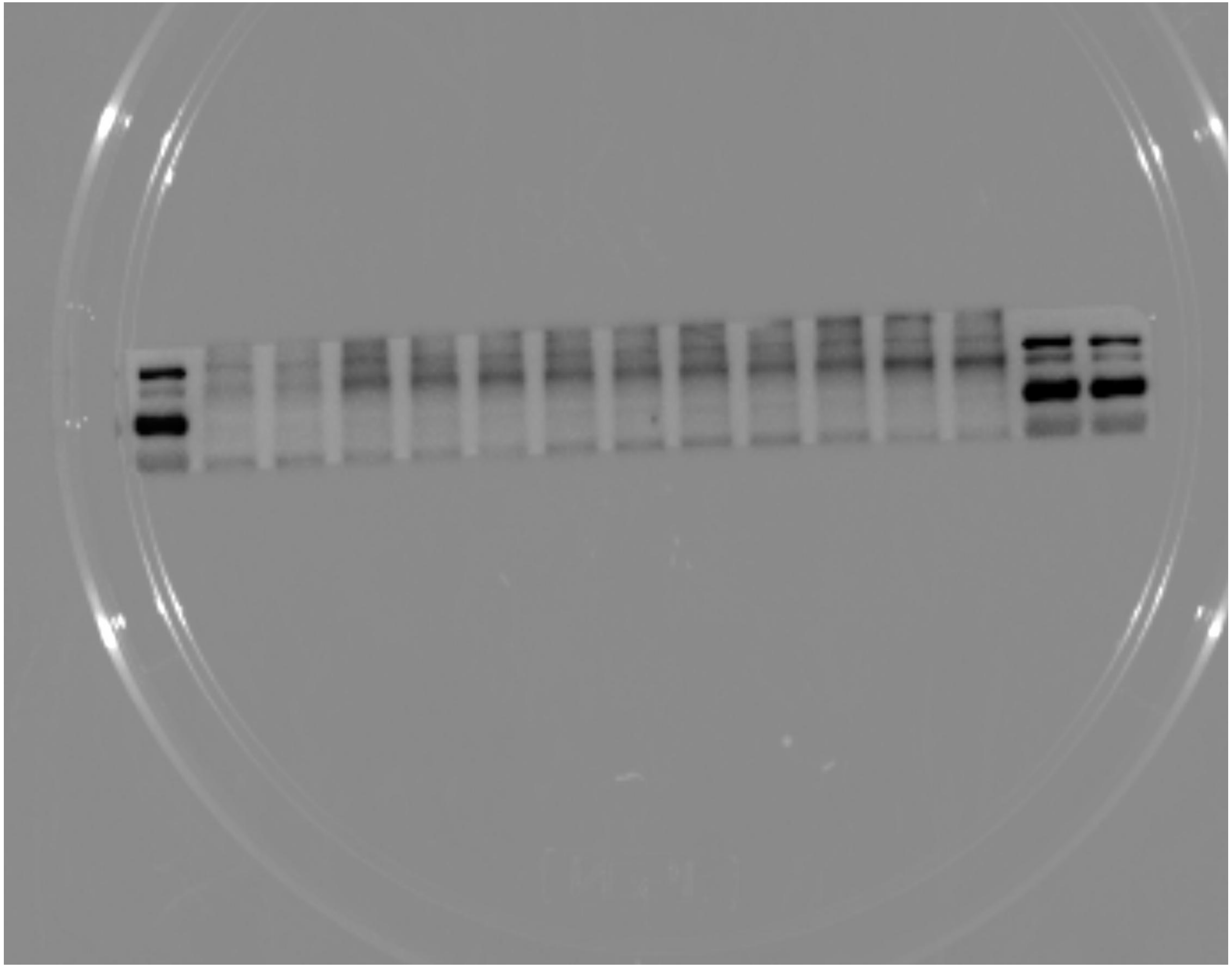

Supplementary Figure-8A 7D VEGF

Merge mode

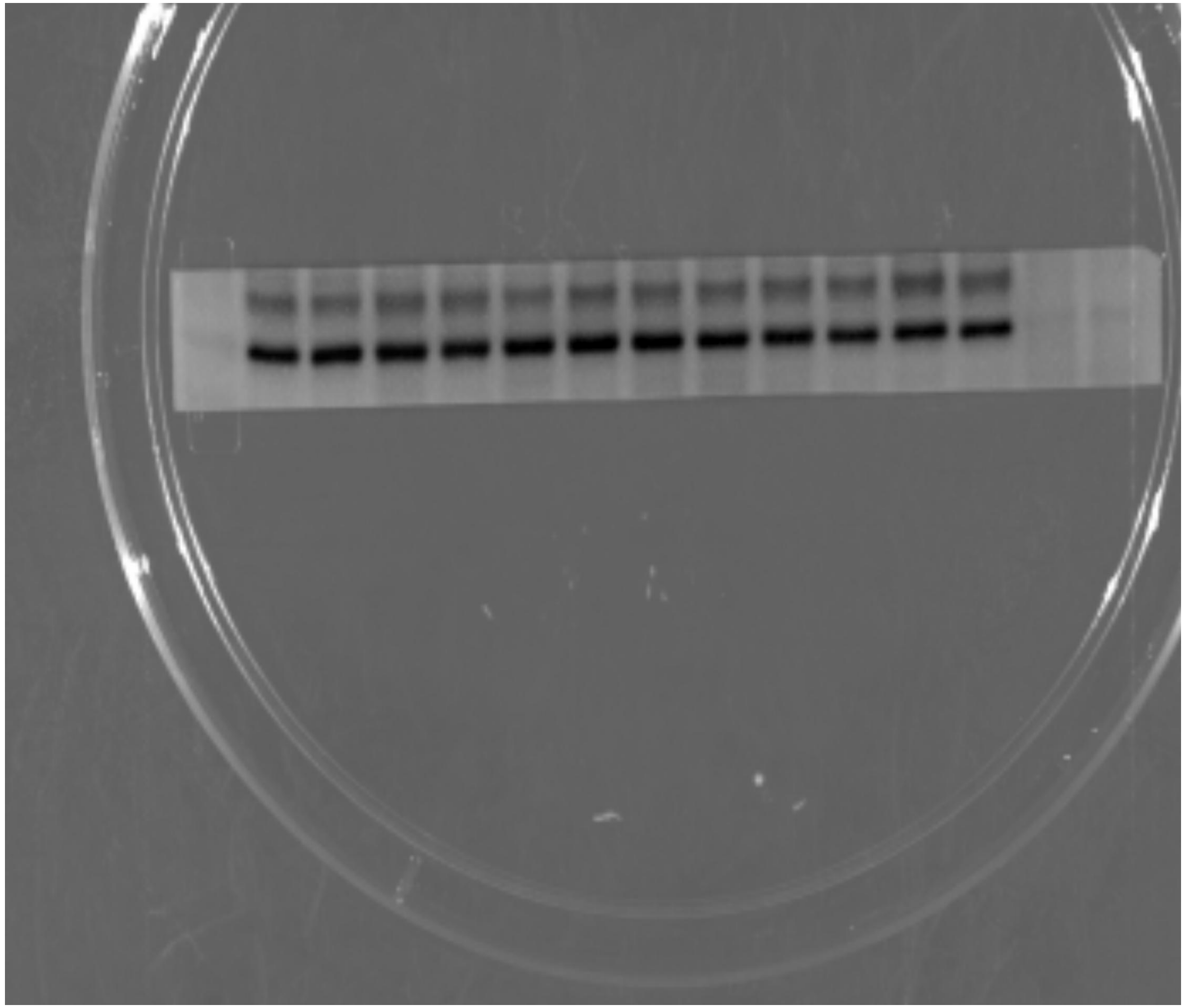

Colormeritric mode

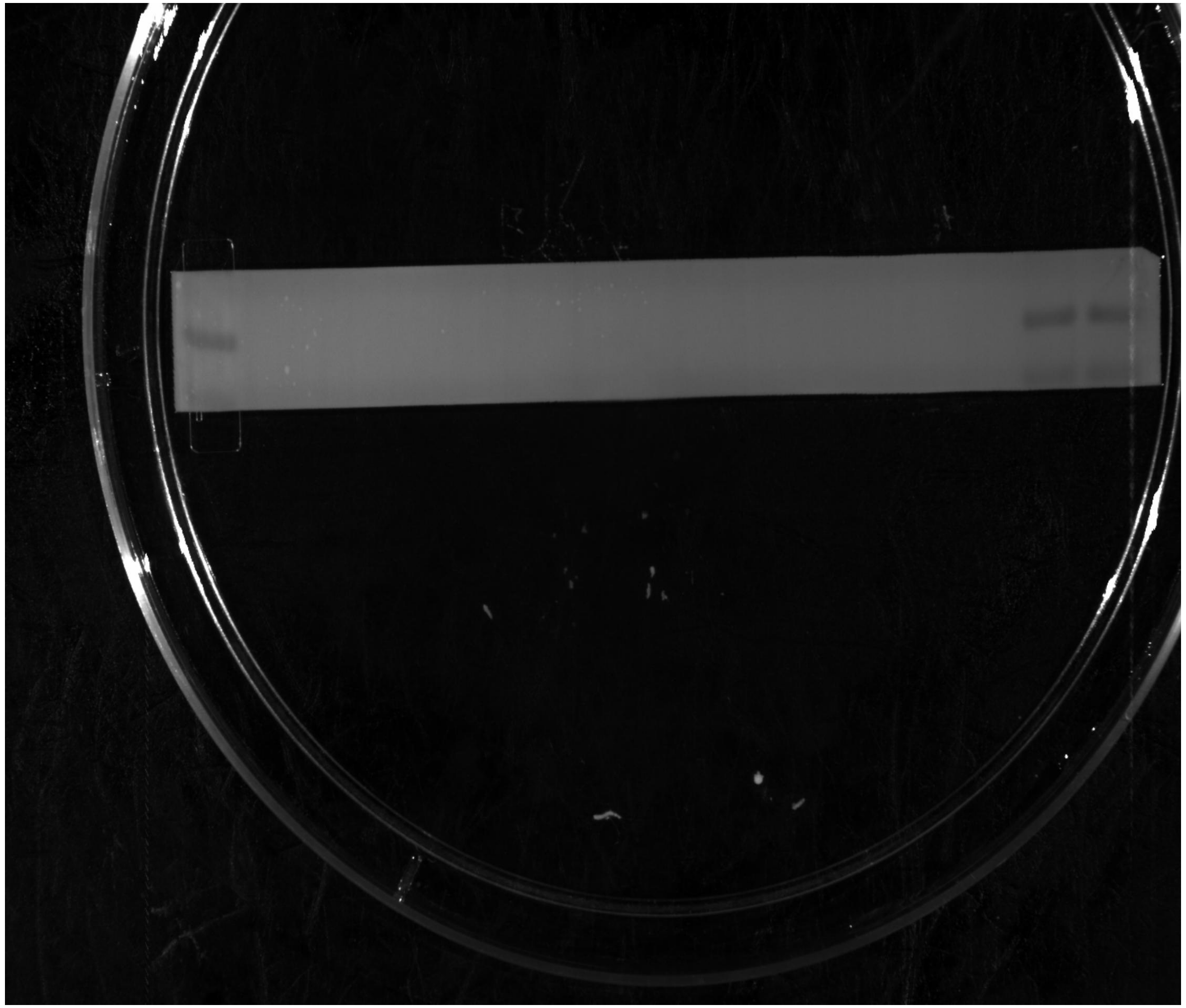

Supplementary Figure-8A 7D GAPDH

Merge mode

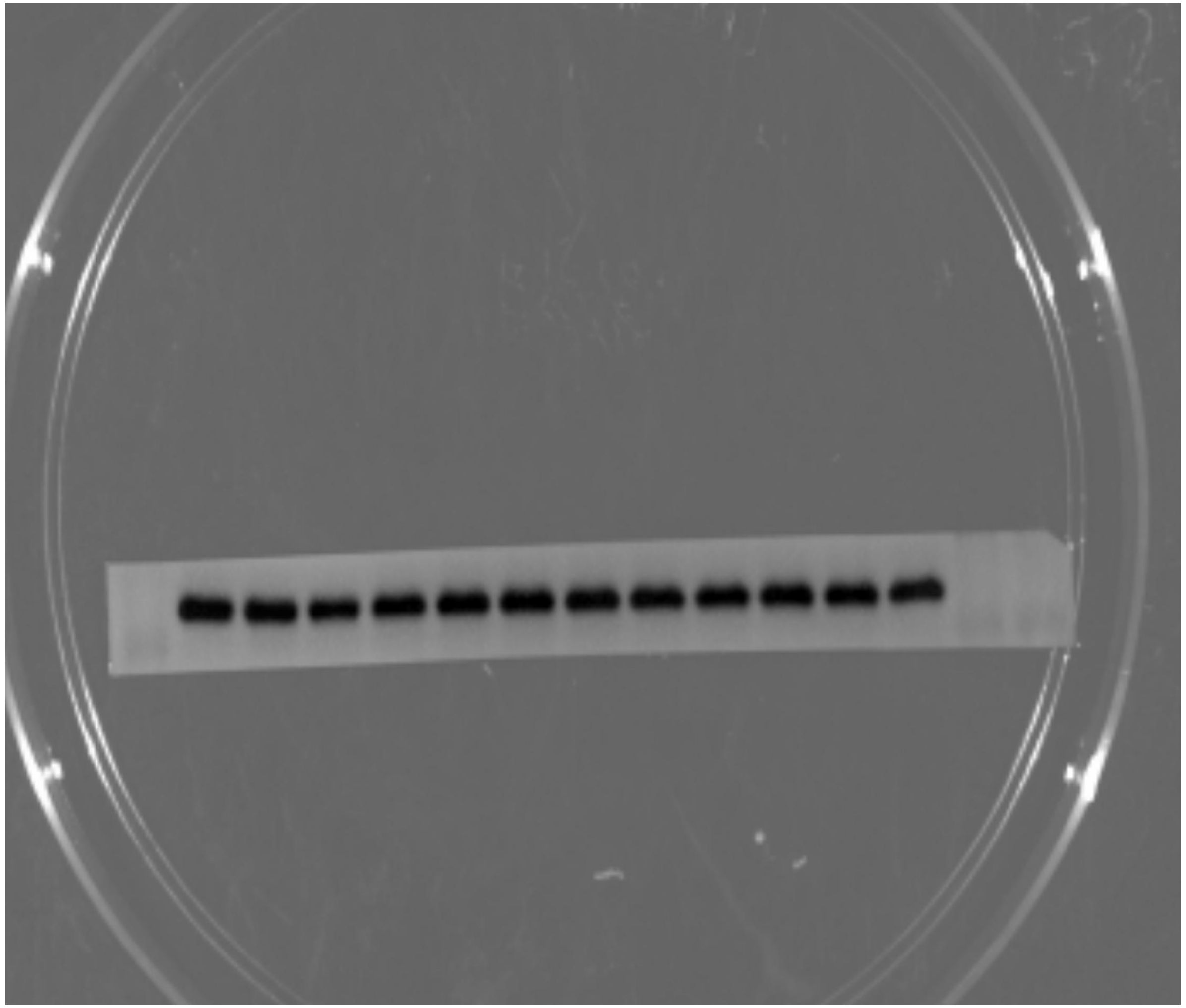

Colormeritric mode

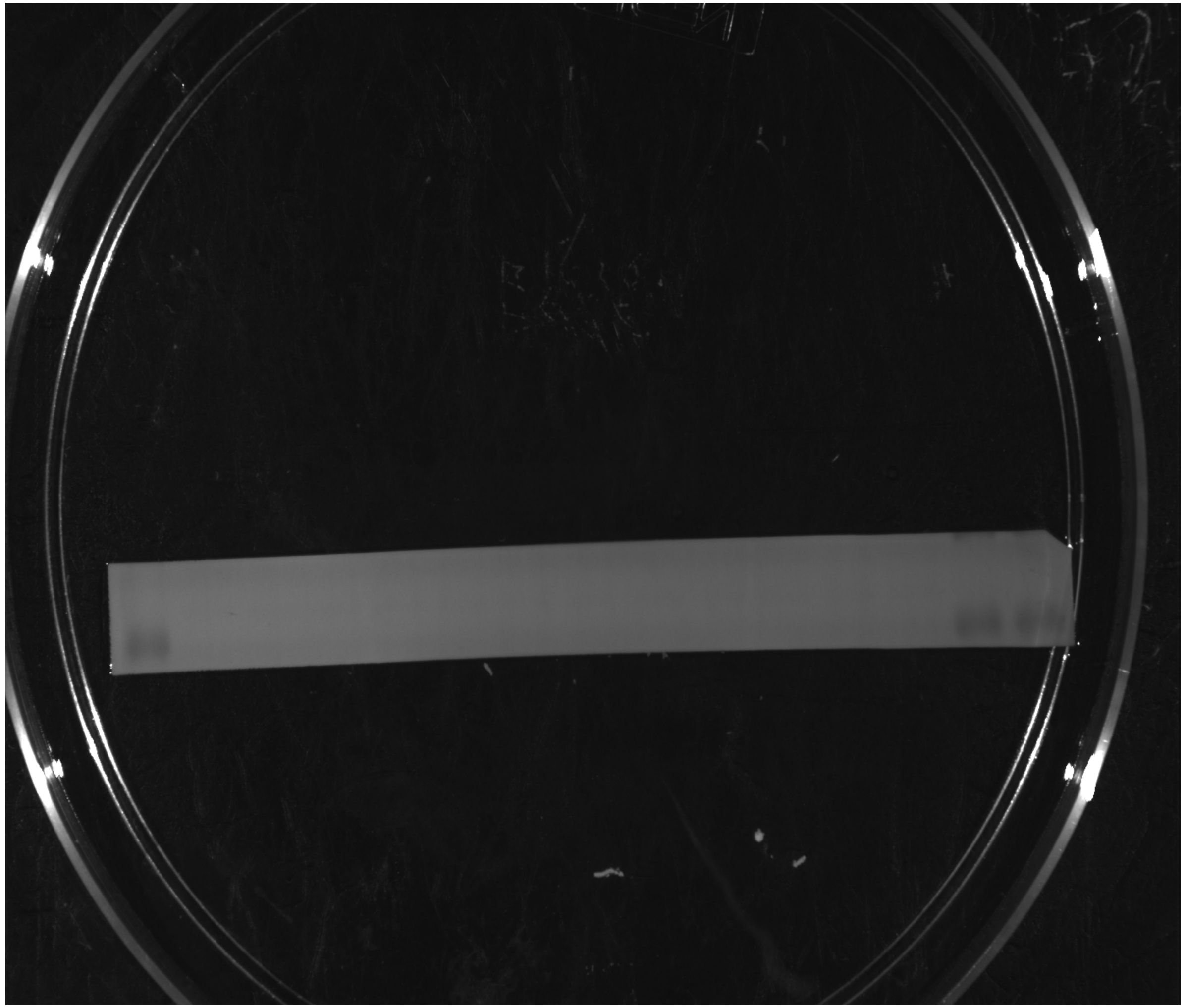

Supplement: Supplementary file 2 — Original Data File [file 41419_2022_4911_MOESM2_ESM.pdf]
